# Supplementary material for: A population‐based temporal logic gate for timing and recording chemical events
Source: Mol Syst Biol. 2016 May 18;12(5):869. doi: 10.15252/msb.20156663 (PMC5289221; doi:10.15252/msb.20156663)
Supplement: Supplementary file 1 — Appendix [file MSB-12-869-s001.pdf]

A population-based temporal logic gate for timing and recording chemical events
APPENDIX

Victoria Hsiao[1,*], Yutaka Hori[2], Paul W. K. Rothemund[3], Richard M. Murray[1]

April 6, 2016

1. Biology and Biological Engineering, California Institute of Technology, Pasadena, CA 91125.
2. Applied Physics and Physico-Informatics, Keio University, Yokohama, Kanagawa 223-8522, Japan.
3. Computation & Neural Systems, California Institute of Technology, Pasadena, CA 91125.

* Corresponding Author: Victoria Hsiao, vhsiao at caltech dot edu.

Subject categories: Synthetic Biology, DNA memory, Stochastic models

Keywords: DNA memory / event detectors / integrases / stochastic biomolecular models / whole cell sensors

Running Title: Two-input integrase event detector

Contents

1 A Markov Model for integrase recombination **4**

2 Characterization of inducer separation time **7**

3 Experimental results for varying inducer separation time **8**

4 Comparing plate reader fluorescence readings with flow cytometry **14**

5 Varying model parameters for integrase activity and leaky basal expression **15**

6 Deducing inducer pulse width: simulations **18**

7 Deducing inducer pulse width: experimental **20**

8 Single colony analysis of pulse modulated populations **26**

9 Model exploration of S_a dependence of Δt **28**

10 Practical use and calibration **30**

11 Fitting equations and reference tables **32**

12 Derivation: A Markov model for integrase-based temporal logic gates **34**
12.1 Mathematical model 34
12.2 Tetramerization of integrases 36
12.3 DNA state S_a is independent of inducer separation time Δt 37

13 List of plasmids and cell strains used **39**

14 Plasmid maps **40**

List of Figures

S1 Nonlinear term for integrase tetramerization 5
S2 Individual cell trajectories from stochastic simulation 6
S3 Effect of varying Δt on number of S_a, S_b, and S_o state cells in simulation 7
S4 RFP expression *in vivo* experiments with increasing Δt 8
S5 *In vivo* GFP expression curves aligned by Δt 9
S6 Color-separated figures for Figure 4 10
S7 OD growth curves for *in vivo* experiments with increasing Δt 11
S8 Flow cytometry populations, RFP vs GFP (Fig. 4) 11
S9 Flow cytometry GFP histograms (Fig. 4) 11
S10 Flow cytometry RFP histograms (Fig. 4) 12
S11 Single colony analysis of non-fluorescent colonies (Fig. 4) 13
S12 Comparison of plate reader with flow cytometry 14
S13 Fitting model parameters for Figure 5C 15
S14 Varying protein production rates to tune Δt_{90} limit 16
S15 Varying protein production rates, timecourse 17
S16 Deducing pulse width, additional states with varying Δt, PW_b (Fig. 6) 18
S17 Unique populations for different combinations of Δt and PW_b (Fig.7) 19
S18 Selected flow cytometry GFP histograms for Fig.7BC 20
S19 Selected flow cytometry RFP histograms for Fig.7BC 21
S20 Population quadrants for Fig.7BC 22
S21 Complete flow cytometry data for Fig.7BC, RFP vs GFP 23
S22 Complete flow cytometry data for Fig.7BC, GFP 24
S23 Complete flow cytometry data for Fig.7BC, RFP 25
S24 Single colony analysis of non-fluorescent colonies in pulse experiments (Fig.7) 27
S25 Simulations with unequal intB transition rates. 29
S26 Fitting experimental data to PW_b, Δt (Fig.8) 30
S27 Resolution for determining PW_b, Δt from population distributions (Fig.8) 31
S28 Plasmid maps of temporal logic gate system 40

List of Tables

S1 Initial Markov transition rates and parameters 4
S2 Table of revised parameters for uneven leakiness and flipping (Fig5) 15
S3 Fitted parameters for $PW_b(R)$, $\Delta t(G, PW_b)$ 32
S4 Generated table of PW_b and Δt based on fitted curves. 33
S5 Markov transitions of the states 34

1 A Markov Model for integrase recombination

Parameters used for simulations

The parameters were chosen to be in biological orders of magnitude. Tetrameterization of the integrase is represented with the expression

$$\alpha_i(\mathrm{Int}_*) := k_{\mathrm{flip}*}\left(\frac{\mathrm{Int}_*(\mathrm{Int}_*-1)(\mathrm{Int}_*-2)(\mathrm{Int}_*-3)}{K_{\mathrm{d}*}^4+K_{\mathrm{d}*}^3\mathrm{Int}_*+K_{\mathrm{d}*}^2\mathrm{Int}_*(\mathrm{Int}_*-1)+K_{\mathrm{d}*}\mathrm{Int}_*(\mathrm{Int}_*-1)(\mathrm{Int}_*-2)+\mathrm{Int}_*(\mathrm{Int}_*-1)(\mathrm{Int}_*-2)(\mathrm{Int}_*-3)}\right) \quad (1)$$

for $i = 1, 2, 3$ and $* = A, B$ (see **Tetramerization of integrase** in Appendix Section 12.2 for the derivation, and Fig. S1).

Parameter	Value	Units
α_1	See equation (15)	
α_2	See equation (15)	
α_3	See equation (15)	
δ_A	$k_{\mathrm{deg}}(\mathrm{IntA})$	
δ_B	$k_{\mathrm{deg}}(\mathrm{IntB})$	
γ_A	$\begin{cases} k_{\mathrm{prodA}} + k_{\mathrm{leakA}}, & \text{if inducer a exists} \\ k_{\mathrm{leak}A}, & \text{if no inducer present} \end{cases}$	
γ_B	$\begin{cases} k_{\mathrm{prod}B} + k_{\mathrm{leak}B}, & \text{if inducer b exists} \\ k_{\mathrm{leak}B}, & \text{if no inducer present} \end{cases}$	
$k_{\mathrm{prod}A,B}$	50	$(\mu\mathrm{m}^3\cdot \mathrm{hr})^{-1}$
k_{deg}	0.3	hr^{-1}
$k_{\mathrm{flip}A}$	0.4	hr^{-1}
$k_{\mathrm{flip}B}$	0.4	hr^{-1}
$k_{\mathrm{leak}A}$	0	$(\mu\mathrm{m}^3\cdot \mathrm{hr})^{-1}$
$k_{\mathrm{leak}B}$	0	$(\mu\mathrm{m}^3\cdot \mathrm{hr})^{-1}$
$K_{\mathrm{d}A}$	10	molecules
$K_{\mathrm{d}B}$	10	molecules

Table S1: Initial Markov transition rates and parameters. We define the rate of DNA state transitions as α_1, α_2 and α_3 using the rate of DNA flipping for a unit concentration of $\mathrm{IntA}(k_{\mathrm{filpA}})$ and $\mathrm{IntB}(k_{\mathrm{flip}B})$. The notations IntA and IntB denote the copy number of each integrase, and $[S_o] = 1([S_a] = 1)$ if the DNA state is $S_o(S_a)$ and $[S_o] = 0([S_a] = 0)$ otherwise. The production and degradation rates of the integrases are defined by γ and δ, respectively. $k_{\mathrm{prod}A}$ and $k_{\mathrm{prod}B}$ are the protein production rate constants, and $k_{\mathrm{leak}A}$ and $k_{\mathrm{leak}B}$ are the basal leaky expression rate constants. We assume the plasmid copy number is proportional to the volume of a cell. In this paper, we use 1 $\mu\mathrm{m}^3$ (1 femtoliter) as the estimated volume of a single *E. coli* cell. The integrase degradation/dilution rate constant $k_{\mathrm{deg}} = 0.3\ \mathrm{hr}^{-1}$ sets the protein half-life to approximately 2.3 hours. The binding constant, $K_{\mathrm{d}*}$ was estimated based on Bxb1 K_d binding constants 70 nM (Singh et al, 2013). When converted into molecules in 1 femtoliter volume, this translated to 7 molecules $(70\mathrm{nM} \times \frac{10^{23}\mathrm{molecules}}{\mathrm{L}} \times 10^{-9} \times \frac{1\mathrm{L}}{10^{15}\mu\mathrm{m}^3} = 7$ copies.)

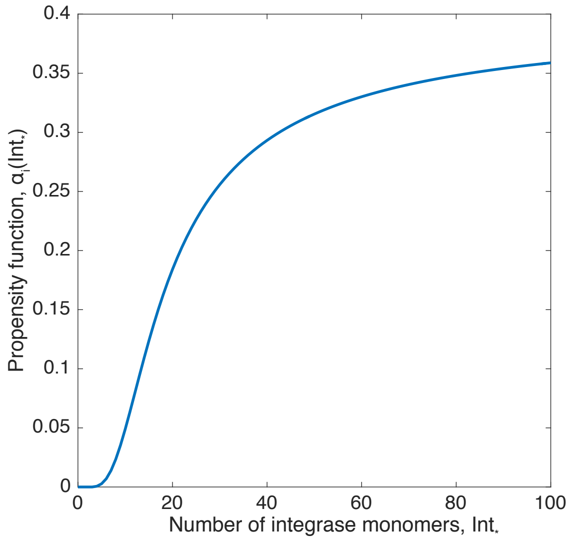

Figure S1: Visualizing the nonlinear term for integrase tetramerization (Eq. 1). The propensity function, $\alpha_i(\mathrm{Int}_*)$, as a function of integrase monomers, Int_*, is zero until at least four monomers are present. Parameters for flipping and dissociation constant are $k_{\mathrm{flip}*}$ = $0.4\ \mathrm{hr}^{-1}$, and $K_{\mathrm{d}*} = 10$ molecules.

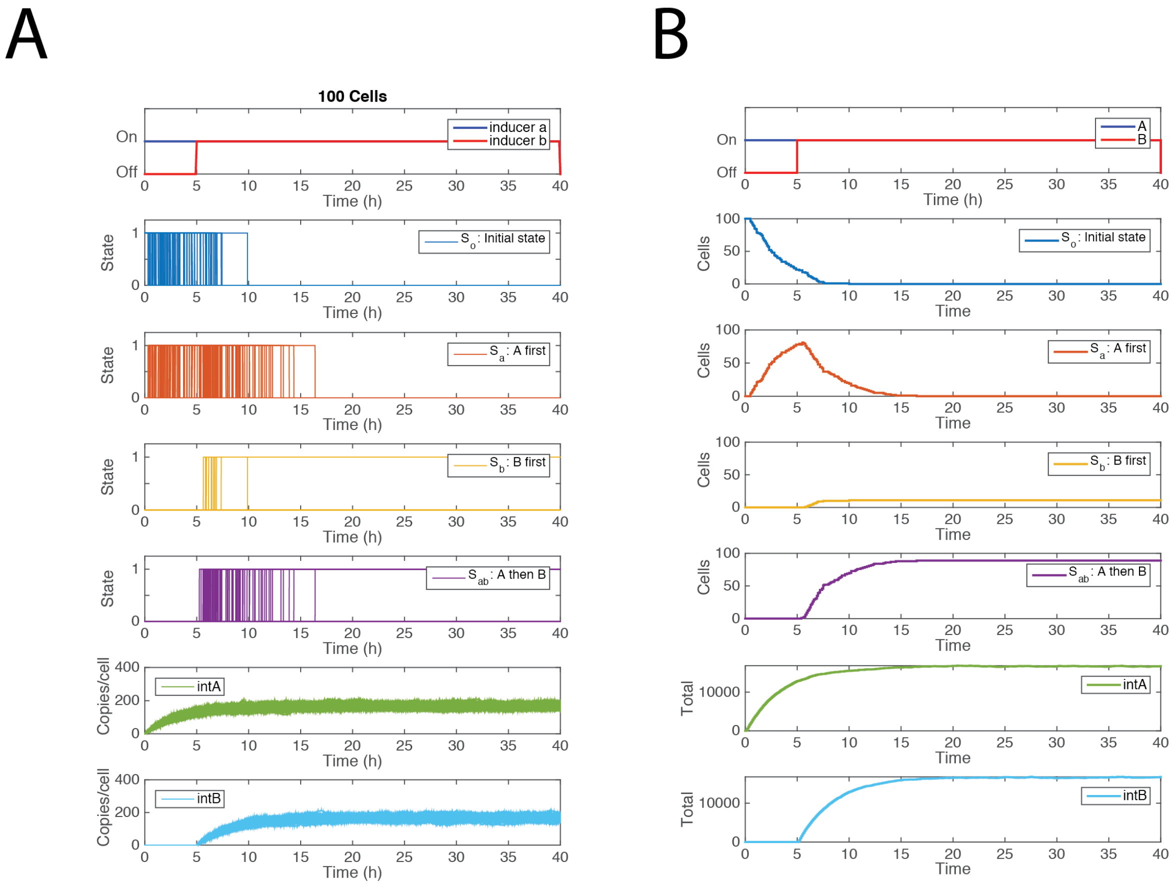

Figure S2: Example of individual cell trajectories and total summed population from stochastic simulations (Δt = 5h). A) Individual simulated cell trajectories for the possible cell states. A sample of 100 cells out of the population of 5000 has been shown here for clarity. The panels, from top to bottom, show time and duration of induction, cells in state S_o (blue), cells in state S_a (red), cells in state S_b (yellow), cells in state S_{ab} (purple), copies per cell of integrase A (green), and copies per cell of integrase B (sky blue). B) Summed totals of all possible DNA and protein states for all 5000 cells.

2 Characterization of inducer separation time

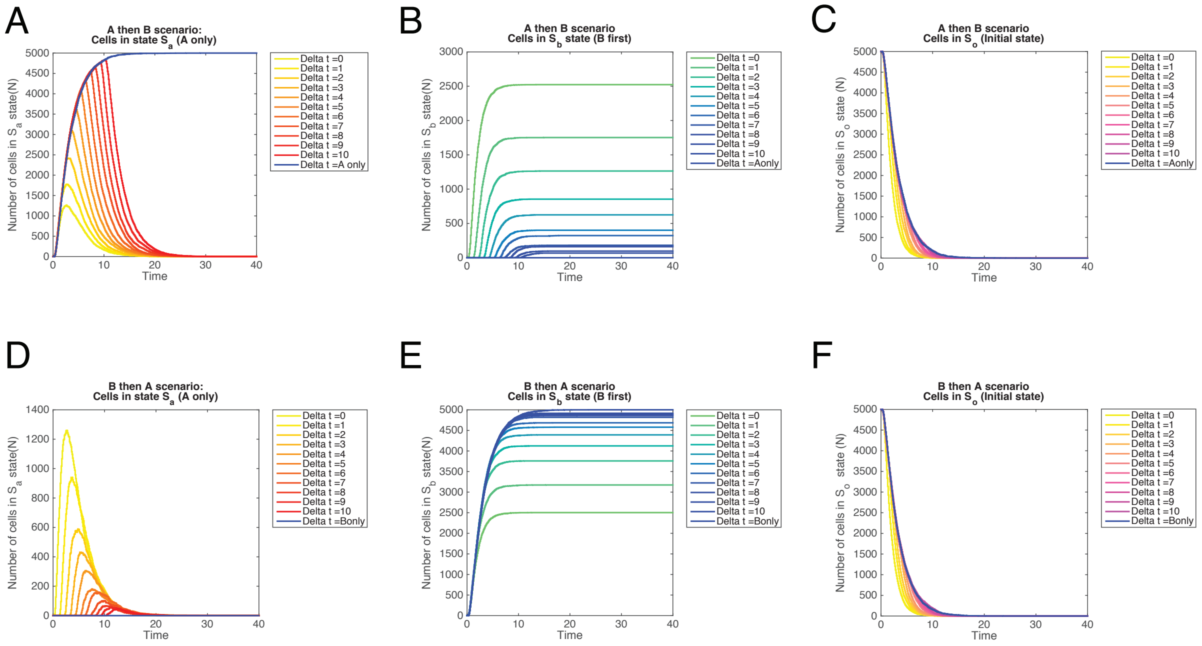

Figure S3: Effect of varying Δt on number of S_a, S_b, and S_o state cells in simulation (Supplementary to Figure 3 in the main text). For each value of Δt, a population of 5000 individual cell trajectories was generated and summed. A) S_a cell counts for E_{ab} event. In the case of *a only*, 100% of the cells become S_a. For the other states, the cell count drops off at time Δt as S_a transition into S_{ab}. B) S_b state cells for E_{ab}. The number of S_b cells that transition is a function of available S_o cells left at time Δt. With high Δt, the most cells are already in S_a. C) S_o state cells for E_{ab} decrease exponentially with time as they convert into either S_a or S_o. D) S_a state cell count with an E_{ba} event are inversely proportional to Δt. E) S_b state cells gain fractional dominance with increasing Δt during with an E_{ba} event. In the case of *b only*, 100% of the cells become S_b. F) S_o state cells decrease exponentially with E_{ba} event as well.

3 Experimental results for varying inducer separation time

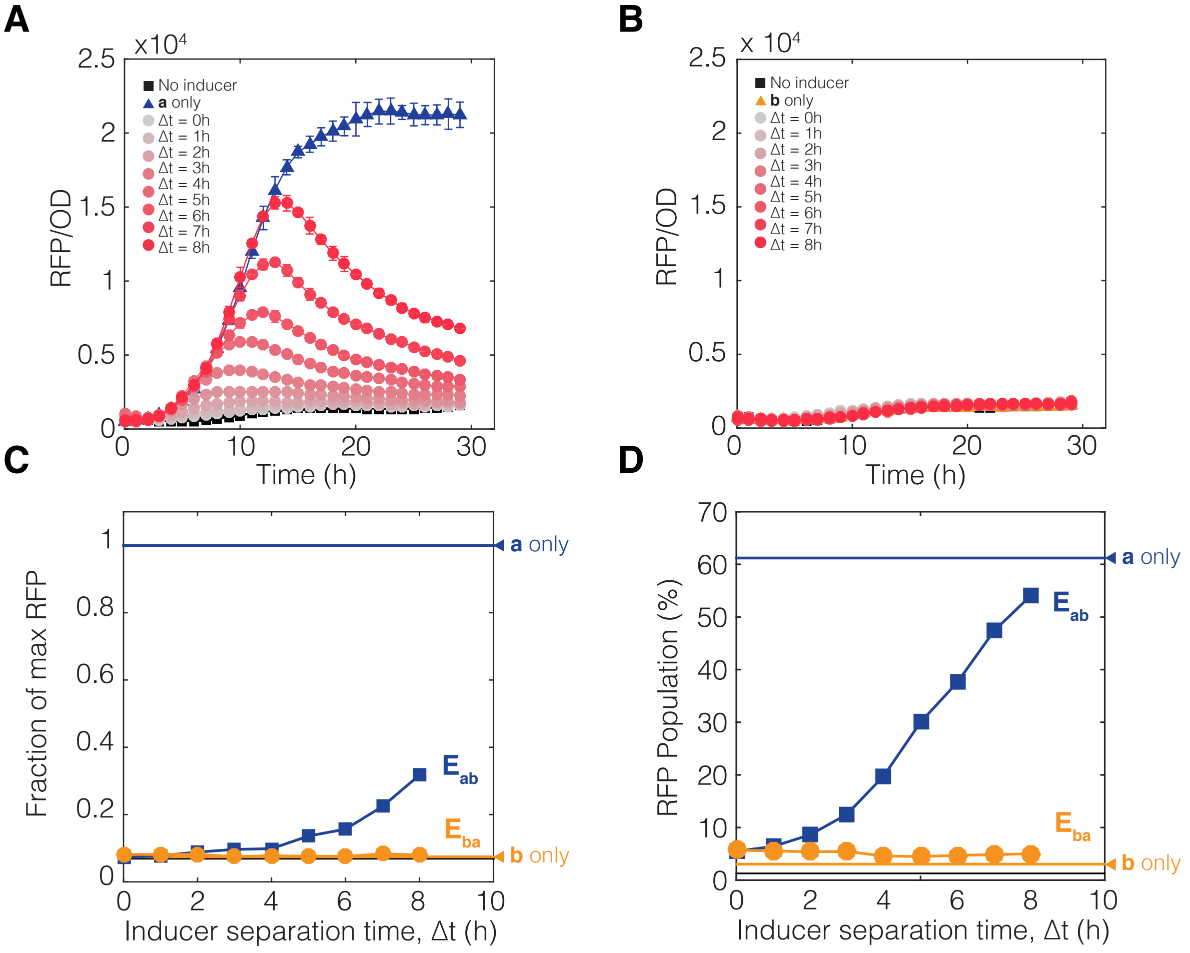

Figure S4: RFP expression for *in vivo* experiments with increasing Δt (Supplementary to Figure 4 in the main text). A) RFP expression as a proxy for S_a state cells when population is exposed to E_{ab}. B) RFP expression when the inverse E_{ba} event occurs. C) Endpoint RFP bulk fluorescence measurement of cultures as a function of Δt. In an infinite step induction experiment, we expect no cells to be expressing RFP since all S_a cells become S_{ab}. However, cultures with later Δt values spend up to 8 hours in S_a and build up a lot of RFP that does not completely dilute even upon switching to S_{ab}. D) Flow cytometry counts of RFP population. The flow cytometry shows that a high percentage of cells are expressing a low amount of RFP. Quadrant analysis of RFP vs GFP populations shows that these RFP-expressing cells are all in Q2, the transitory quadrant in which cells have switched to S_{ab} but still have undiluted RFP molecules (Figure EV1). See also Appendix Figure S12 for conversion between flow populations and bulk intensity measurements.

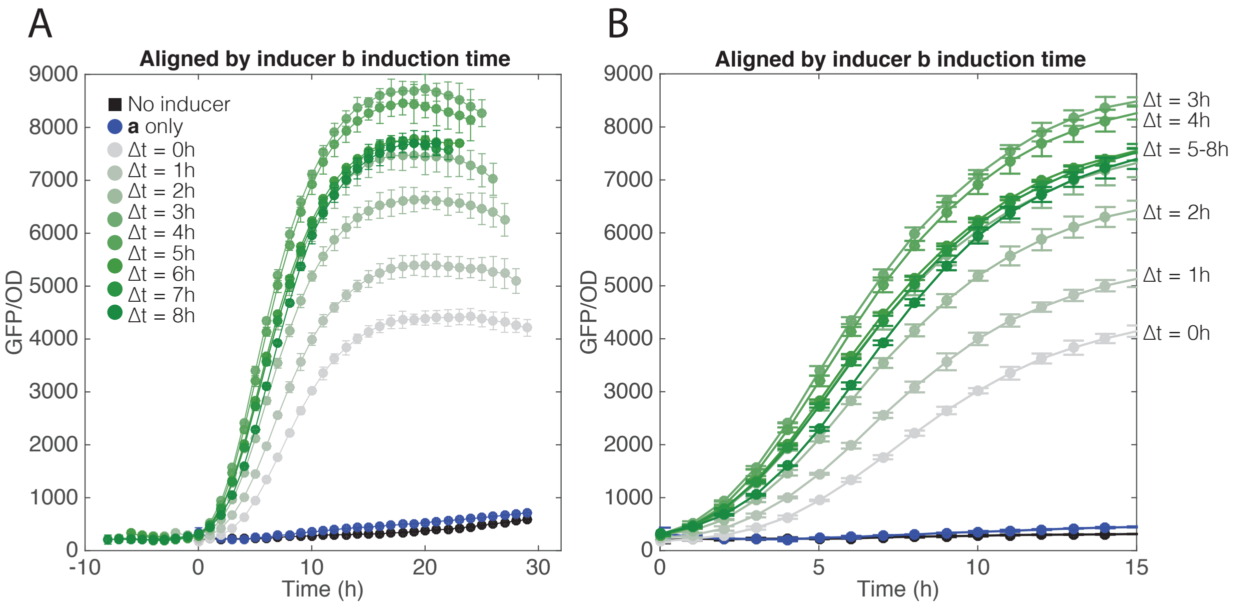

Figure S5: *In vivo* GFP expression curves aligned by Δt (Supplementary to Figure 4 in the main text). A) Curves have been aligned by Δt such that cell switching to S_{ab} and GFP expression all starts at time 0. B) Zoomed in panel shows different slopes of the various Δt curves.

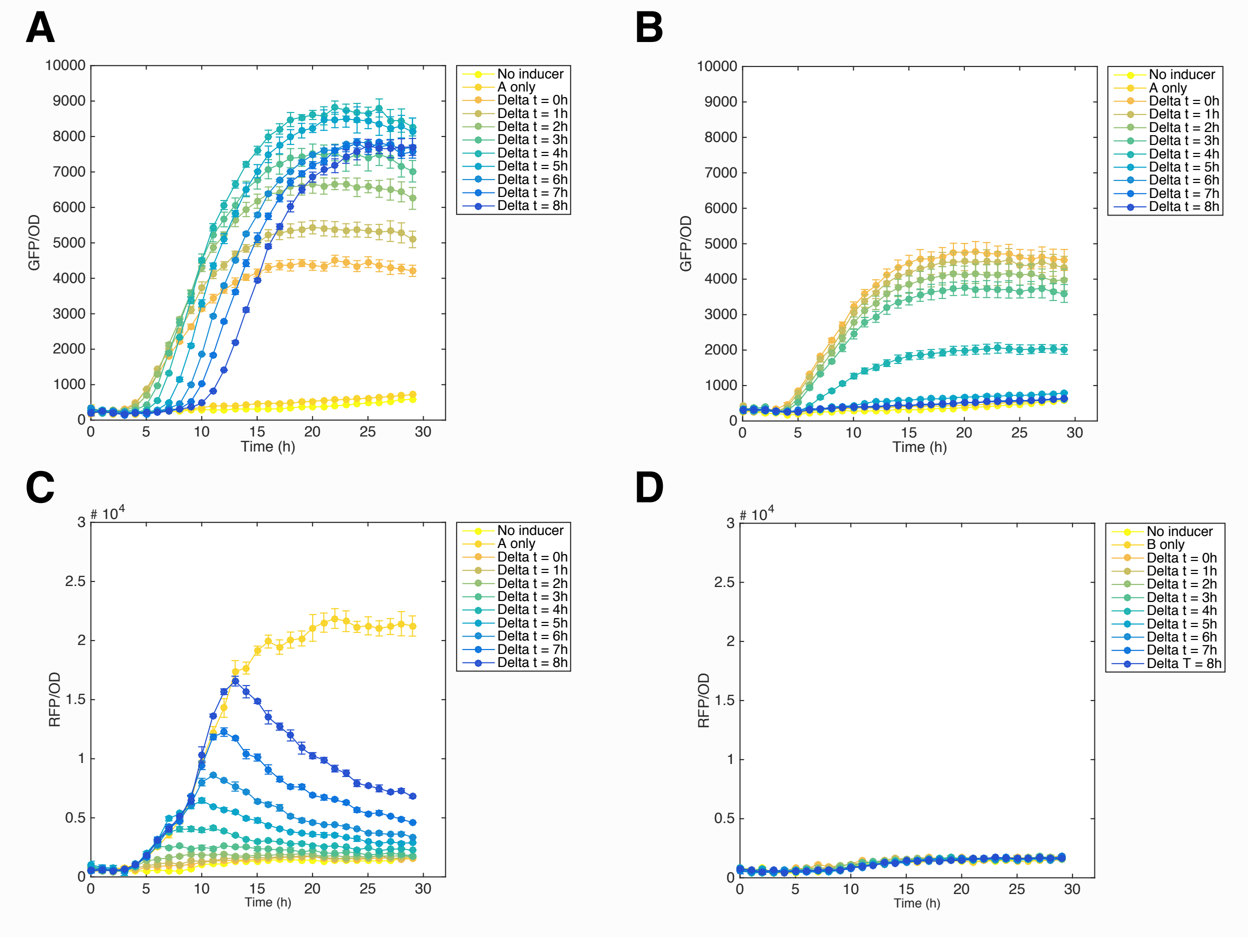

Figure S6: Time-course data for Figure 4 with more separated color scheme. The color gradient used in Figure 4 can make it difficult to distinguish individual curves, and so here we have more color-separated plots. A) GFP fluorescence with event E_{ab}. B) GFP fluorescence with event E_{ba}. C) RFP fluorescence with E_{ab}. D) RFP fluorescence with E_{ba}.

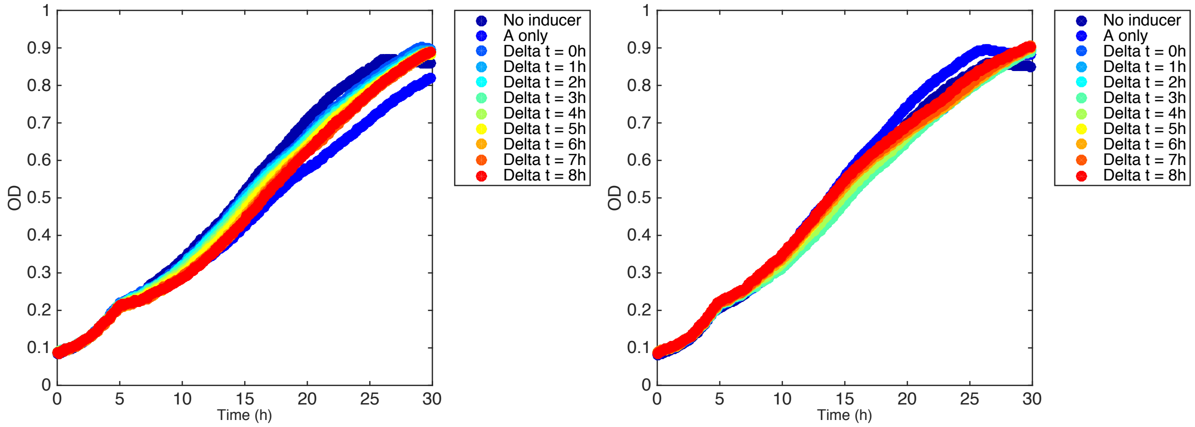

Figure S7: OD growth curves for *in vivo* experiments with increasing Δt (Supplementary to Figure 4 in the main text). Growth curves are fairly linear due to growth in M9CA minimal media at 37C. A) OD growth curves for cells subjected to E_{ab} event. B) OD growth curves for cells subjected to E_{ba} event

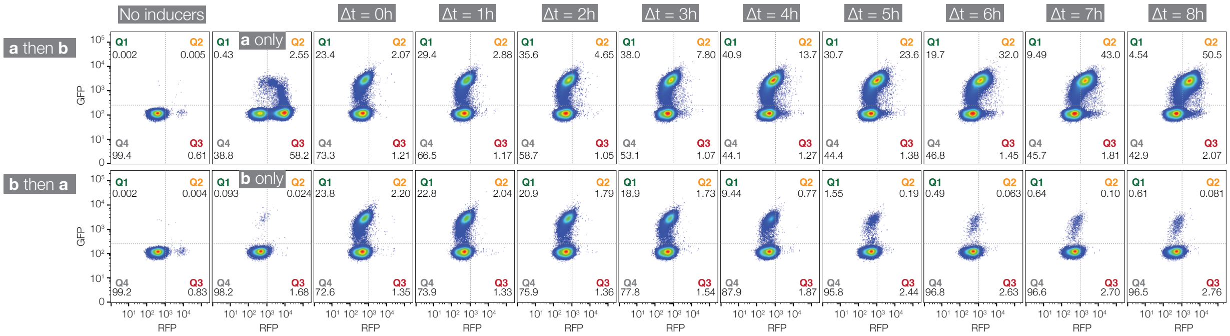

Figure S8: Flow cytometry populations, RFP vs GFP (Fig. 4). Populations are split into quadrants Q1 (GFP only, S_{ab}), Q2 (GFP + RFP, S_{ab}), Q3 (RFP only, S_a), and Q4 (non-fluorescent, $S_o + S_b$.) $\sim$ 100,000 cells were analyzed for each population.

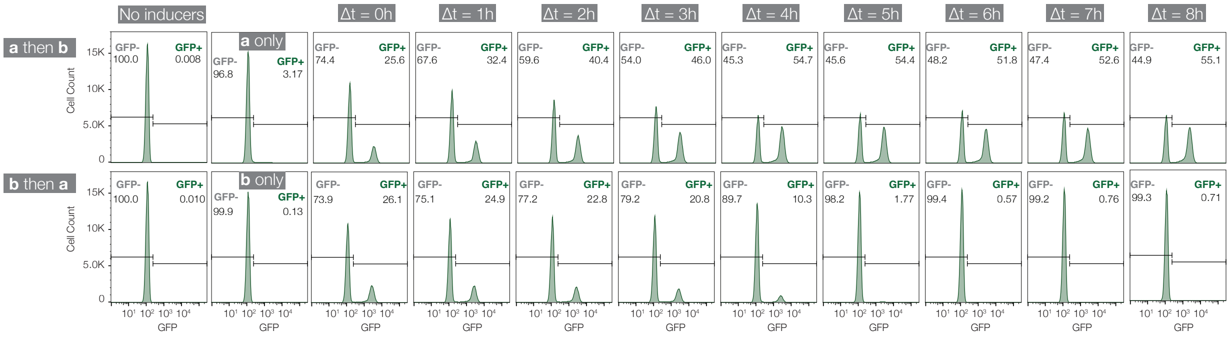

Figure S9: Flow cytometry GFP histograms (Fig. 4). $\sim$ 100,000 cells were analyzed for each population.

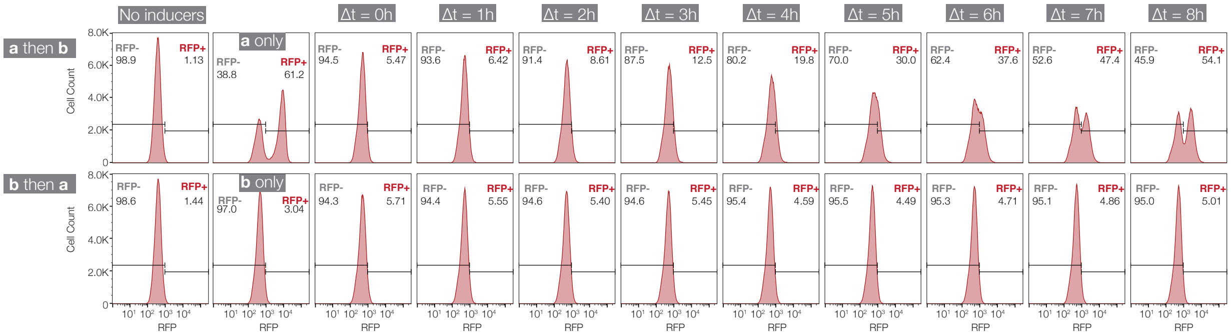

Figure S10: Flow cytometry RFP histograms (Fig. 4). ∼ 100,000 cells were analyzed for each population.

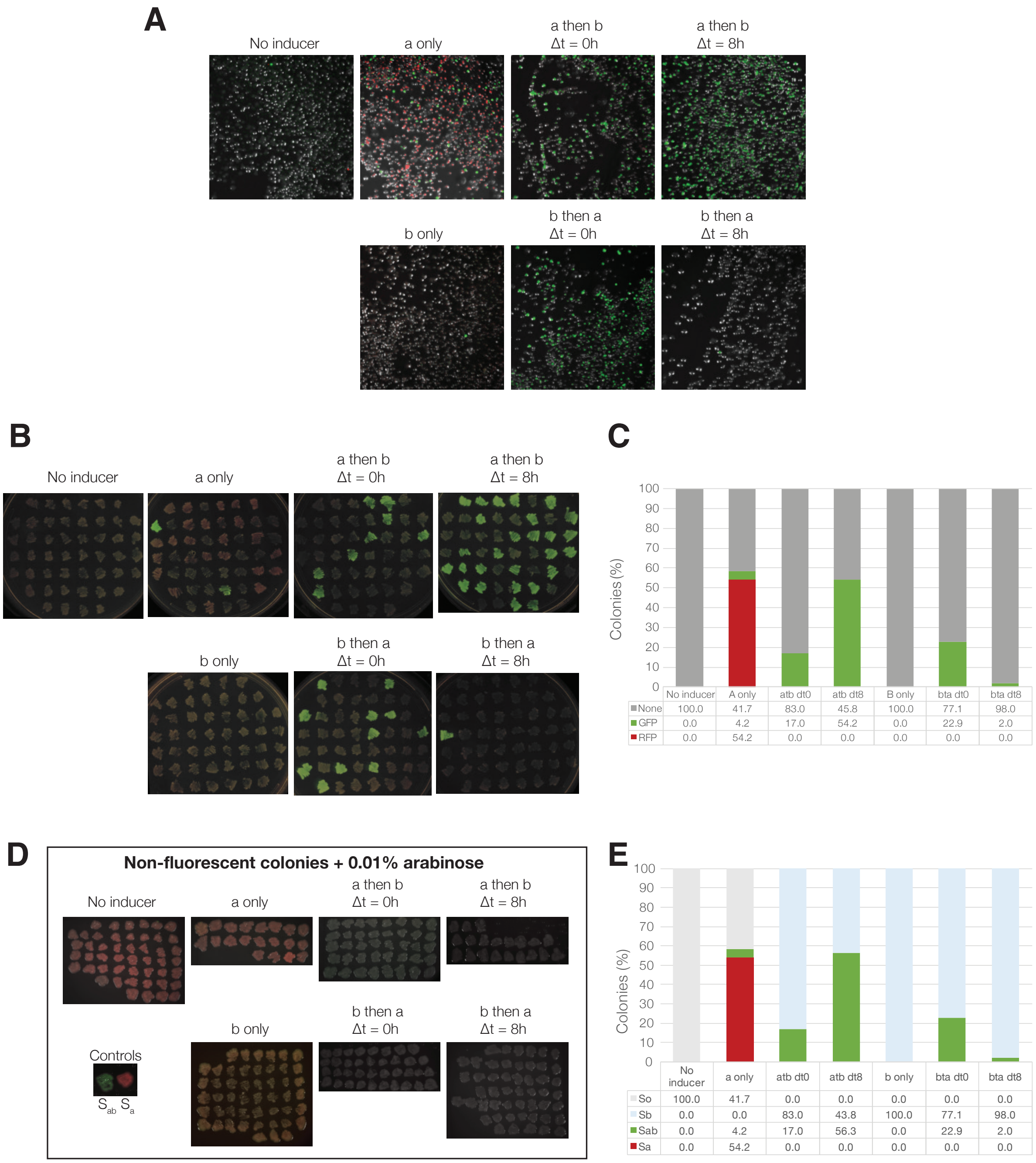

Figure S11: Single colony analysis of non-fluorescent colonies in Δt experiment (Fig. 4) was done to determine genetic state (S_o or S_b). A) Experimental cultures were diluted 1:10,000 after experiment and plated on LB agar plates with no inducer. B) 48 ± 2 single colonies were randomly picked from each plate and re-streaked on a new agar plate. C) Single colonies were counted based on fluorescence and the resulting distributions are similar to those measured via flow cytometry. D) The non-fluorescent colonies from each condition were then re-streaked again onto plates with 0.01% arabinose. Only S_o cells would turn red (S_a), while S_b cells would remain non-fluorescent. We determined that 100% of the *no inducer* and *a only* non-fluorescent colonies were S_o, while the 100% of the non-fluorescent colonies in the other experimental conditions were S_b. E) Revised genetic state distributions based on single colony analysis of non-fluorescent colonies.

4 Comparing plate reader fluorescence readings with flow cytometry

We were interested to know how bulk culture florescence compared with actual single cell expression profiles. With bulk fluorescence, it is possible that bimodal expression of fluorescent molecules result in a few bright cells dominating the overall fluorescence measurement, and so we wanted to ensure that this was not the case with our time-course measurements. Endpoint bulk fluorescence was measured via BioTek Synergy H1F plate reader (BioTek Instruments, Inc, VT, USA) and normalized by the maximum fluorescence. Flow cytometry was done with a MACSQuant VYB flow cytometer (Miltenyi Biotec, Germany), and for both RFP and GFP, cells were counted and their relative fluorescence intensity was measured. Flow cytometry data was gated using FlowJo Version 10.0.8r1 (Flowjo, LLC, Ashland, OR).

In Figure S12, we compare bulk fluorescence (Fig. S12A) with flow cytometry populations (Fig. S12B), then reconstruct the bulk fluorescence measurements by multiplying the cell counts with average measured intensity (Fig. S12C). We find that GFP fluorescence is not disproportionately skewed by bulk fluorescence, indicating that cells that are “on” in state S_{ab} have a relatively tight distribution and are not overly dominated by a minority of bright cells. This can also be seen in the GFP histograms (Figure S9). For this experiment, in which both inducers are present long after Δt induction, we would expect no cells to remain in state S_a. However, we still measure RFP at the endpoint. We find that this RFP is leftover RFP from cultures that spent more time in S_a prior to transitioning to S_{ab} (Figure EV1). These cells have stopped production of RFP, but existing RFP concentrations have not yet diluted completely. Flow cytometry analysis of cell counts find a high number of cells with low RFP fluorescence. This can also be seen in the RFP histograms (Figure S10).

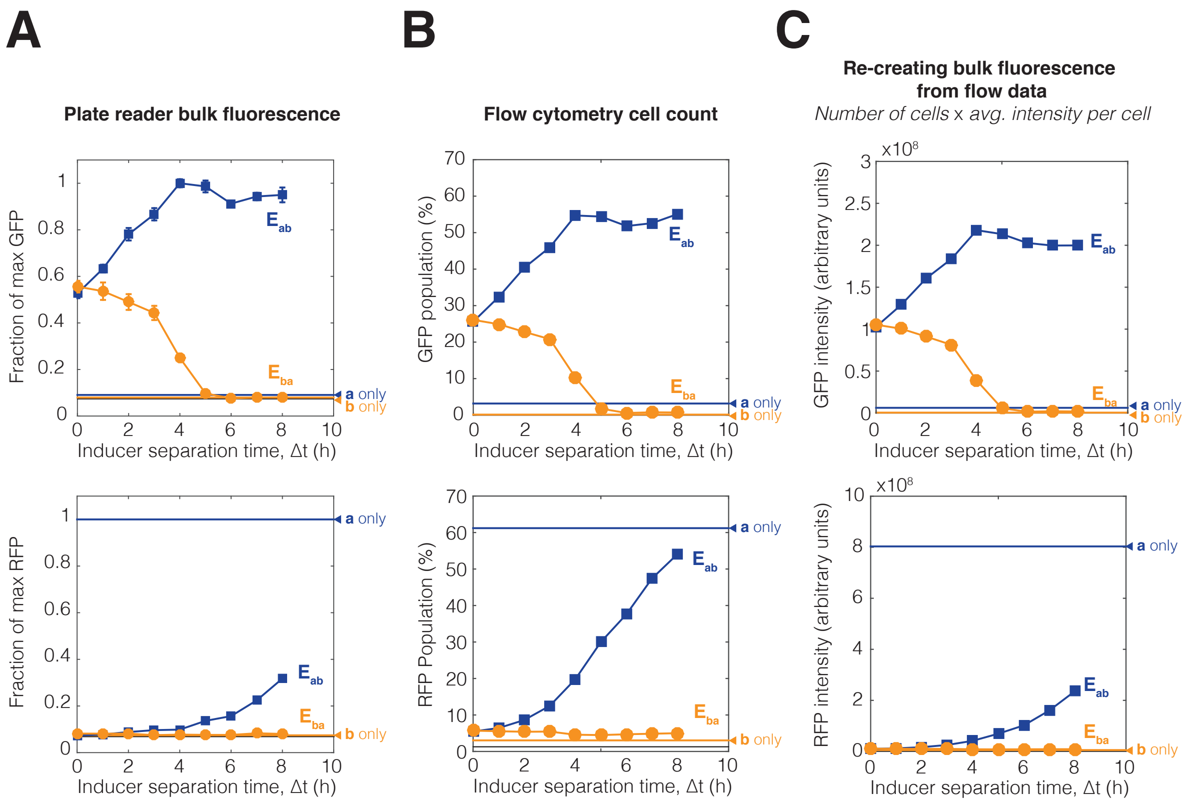

Figure S12: Comparison of plate reader bulk fluorescence readings with flow cytometry cell counts. A) Bulk fluorescence GFP and RFP readings normalized by max GFP and max RFP. B) Flow cytometry counts of cell percentages about GFP and RFP gated thresholds. C) Re-creating bulk fluorescence data from average intensity per cell multipled by number of cells.

5 Varying model parameters for integrase activity and leaky basal expression

Parameter	Value	Units
$k_{\mathrm{prod}A}$	50	$(\mu\mathrm{m}^3 \cdot \mathrm{hr})^{-1}$
$k_{\mathrm{prod}B}$	50	$(\mu\mathrm{m}^3 \cdot \mathrm{hr})^{-1}$
k_{deg}	0.3	hr^{-1}
$k_{\mathrm{flip}A}$	0.2	hr^{-1}
$k_{\mathrm{flip}B}$	0.3	hr^{-1}
$k_{\mathrm{leak}A}$	0.01*$k_{\mathrm{prod}A}$	$(\mu\mathrm{m}^3 \cdot \mathrm{hr})^{-1}$
$k_{\mathrm{leak}B}$	0.02*$k_{\mathrm{prod}B}$	$(\mu\mathrm{m}^3 \cdot \mathrm{hr})^{-1}$

Table S2: Table of revised parameters for uneven flipping to better match experimental data. IntA was set to be less efficient in flipping, and leakiness was added.

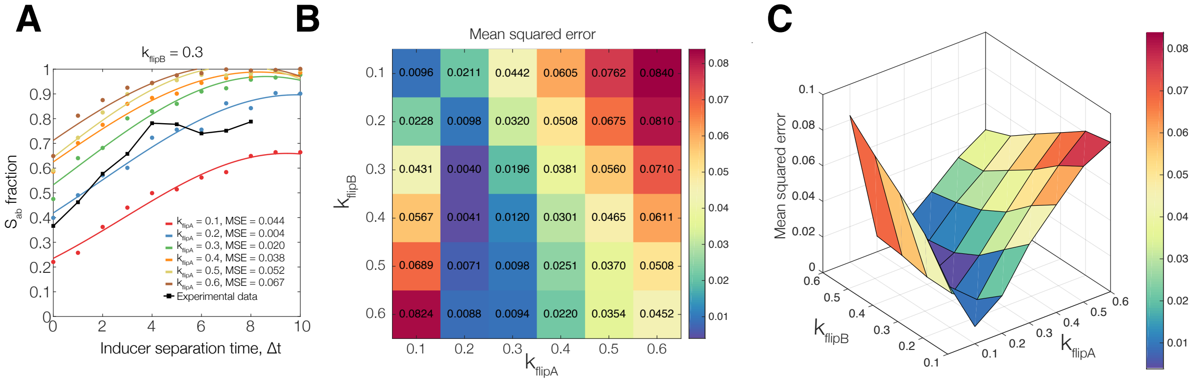

Figure S13: Fitting model parameters for Figure 5C. In stochastic simulations, the flipping efficiency parameters for both integrases, $k_{\mathrm{flip}A,B}$, were varied from 0.1 to 0.6 hr^{-1} for E_{ab} (N = 500 cell trajectories). Leaky basal expression of the integrases were held constant based on experimentally measured values (k_{leak_a} = 1% of $k_{\mathrm{prod}A}$, k_{leak_b} = 2% of $k_{\mathrm{prod}B}$). A) Simulation results for each set of $k_{\mathrm{flip}A,B}$ parameters were fit to a one-term Gaussian function (MATLAB, *fit(x,y,'gauss1')*). Mean squared error (MSE) was calculated by comparing the fitted curves to experimental data from Figure 4C (MATLAB, *goodnessoffit(reference,model)*). This graph shows fits from varying $k_{\mathrm{flip}A}$ for constant $k_{\mathrm{flip}B} = 0.3\mathrm{hr}^{-1}$. Experimental data is shown in black. B) Heatmap showing MSE values for combinations of $k_{\mathrm{flip}A,B}$ parameters. Lower MSE values indicate a better fit. Best fit is for $k_{\mathrm{flip}B} = 0.3\mathrm{hr}^{-1}$, $k_{\mathrm{flip}A} = 0.2\mathrm{hr}^{-1}$. C) Surface plot showing MSE values for combinations of $k_{\mathrm{flip}A,B}$. Lower MSE values indicate a better fit.

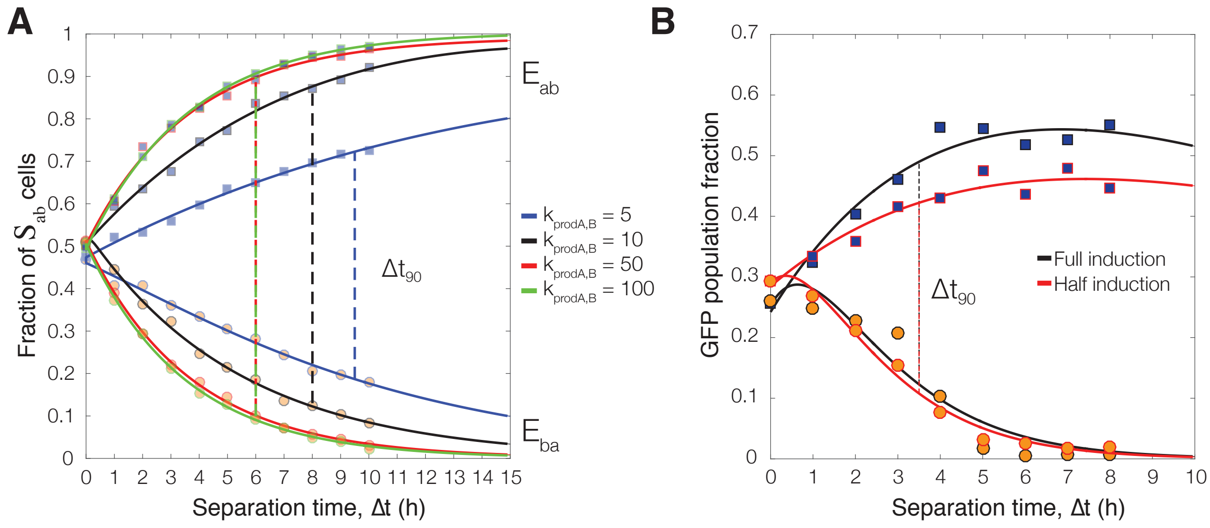

Figure S14: Varying protein production rates to tune the Δt_{90} limit. A) Simulation results for varying $k_{\text{prodA,B}}$ from 5 to 100 $(\mu\text{m}^3 \cdot \text{hr})^{-1}$(N = 2500 per population). Δt_{90} is the limit with which S_{ab} population fractions can be used to resolve unique Δt values, and therefore determines overall system sensitivity to inputs. Based on simulation results, Δt_{90} is inversely proportional to protein production rate. Curve fits were generated for each set of simulated populations (MATLAB, 2-term exponential fit) in order to find Δt_{90}. B) Experimental results show lower Δt_{90} at half induction of integrases. Protein production rate was modulated by reducing the concentrations of the inducers. We compared population-level responses with full inducer concentrations (ara: 0.01%/vol, aTc: 200ng/ml) and half inducer concentrations (ara: 0.005%/vol, aTc: 100ng/ml). The data was fit to a 2-term exponential function (MATLAB, 2-term exponential fit) and the Δt_{90} limit was estimated based on the fitted curve. The Δt values are consistent with being in the saturation regime of integrase production.

Simulation results suggest that the Δt_{90} detection limit can be tuned by increasing or decreasing the overall production rate $k_{\text{prod}*}$ ($*$ = A or B) (Appendix Figure S14). In Figure 4C, the Δt_{90} limit was $\sim$ 4 hours, meaning that within the 0 – 4 hour window, S_{ab} population fraction can be used to uniquely determine Δt. Outside of this window, the only assertion that can be made is that $\Delta t > 5$ hours. *In silico*, we see that the rate of protein production is inversely proportional to the Δt_{90} detection limit (Appendix Figure S14A). When $k_{\text{prod}A,B}$ is high, integrase molecules accumulate faster, increasing the probability of DNA flipping, and thus causing the S_{ab} population fraction to saturate at lower Δt values. However, within that smaller time window, S_{ab} fractions would also be measurably different at much smaller intervals, and so Δt could be resolved with much higher resolution. When protein production is slow, the stochastic DNA recombination events happen less frequently, resulting in a population that is more sensitive to inputs for a longer period of time (high Δt_{90}), but has lower resolution overall since the population fractions are not changing as quickly. These simulation results were compared to some preliminary experimental data in which lower production rates for intA and intB were approximated by halving the inducer concentrations for both **a** and **b** (Appendix Figure S14B, S15). Δt_{90} was estimated by fitting curves to the experimental data to determine maximum S_{ab} (MATLAB, 2-term exponential fit). When inducer concentrations were halved (ara: 0.005%/vol, aTc: 100ng/ml), we see that the Δt_{90} is the same as before, so even with half induction, we are still in the saturation regime of integrase production.

Varying protein production rates more accurately is something we would like to pursue further. We limited the scope of this study to a single concentration of inducer and Δt_{90} such that we could fully understand the information that can be gained from other states in the system.

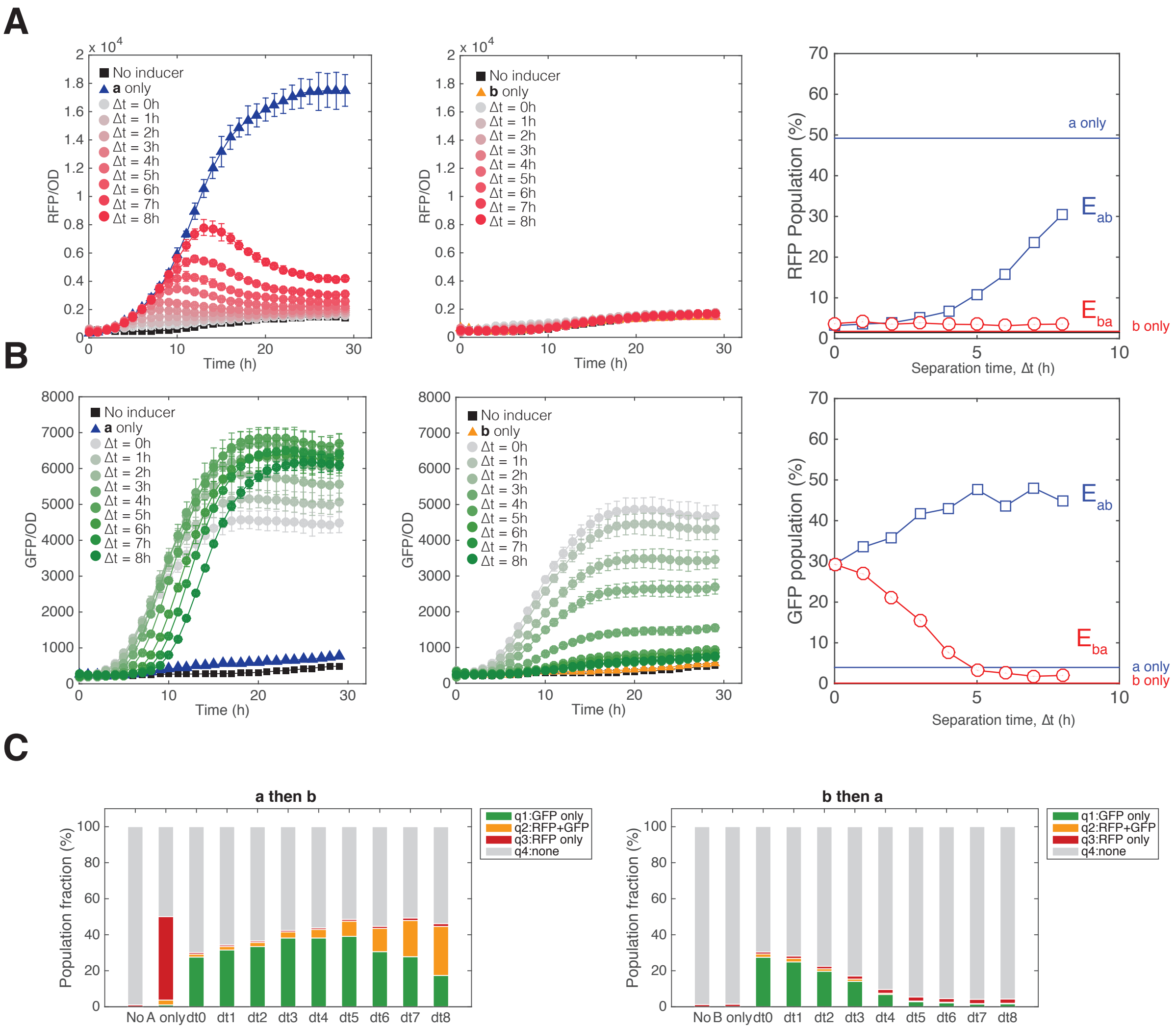

Figure S15: Varying protein production rates, timecourse. Here, we have used half the normal inducer concentrations to test the effects of lower protein production rates. Concentrations of **a** and **b** are 0.005%/vol arabinose and 100ng/ml aTc. A) RFP fluorescence over time for E_{ab}(left), E_{ba}(center), and endpoint population fractions as measured by flow cytometry (right). B) GFP fluorescence over time for E_{ab}(left), E_{ba}(center), and endpoint population fractions as measured by flow cytometry (right). C) Population distributions gated by quadrants. Overall population behavior was the same as full inducers, but response was more graded with increasing Δt.

6 Deducing inducer pulse width: simulations

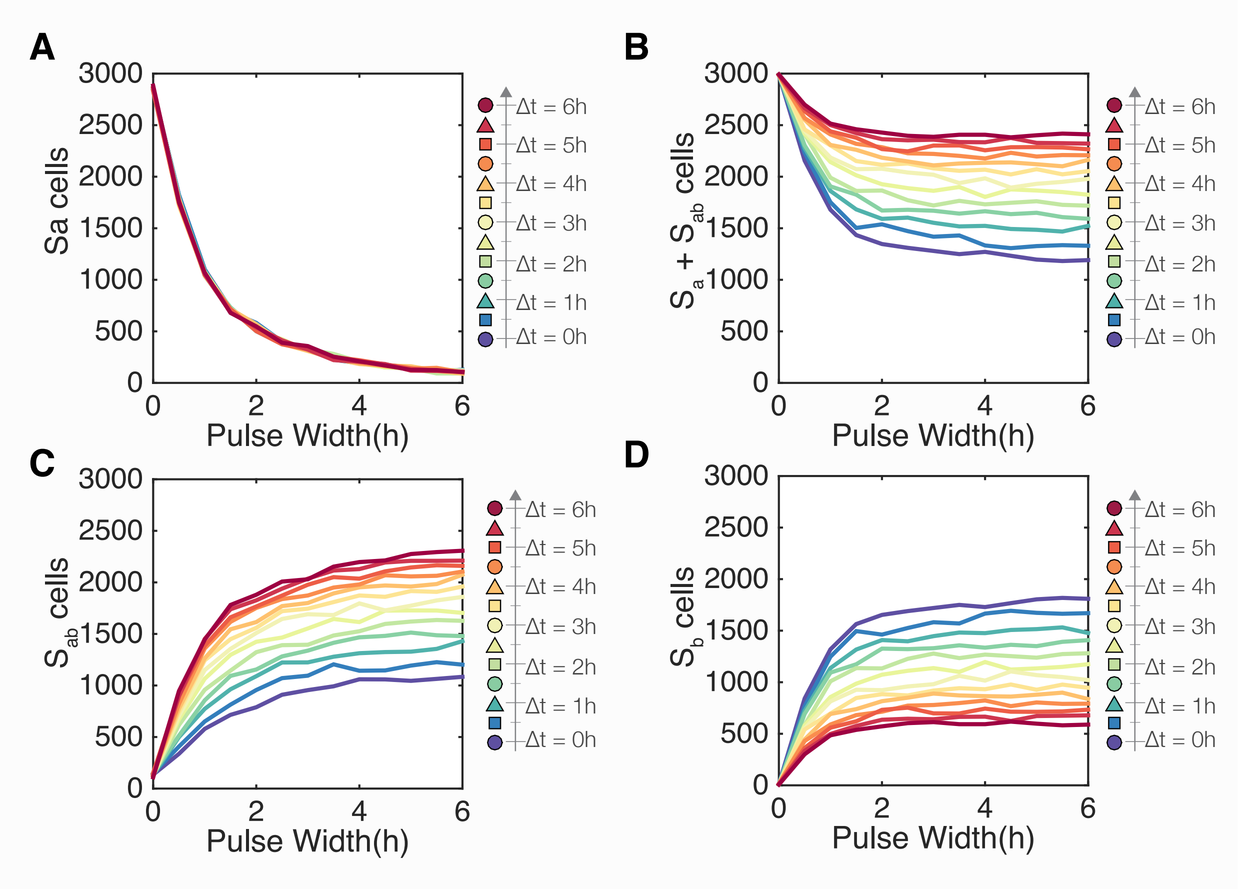

Figure S16: Deducing pulse width, additional states with varying Δt, PW_b, N = 3000 cells. A) S_a cells decrease as function of PW_b. S_a fraction is independent of Δt. B) The sum of S_a + S_{ab} is the fraction of cells that see ***a*** *first*, and this increases with Δt and PW_b. C) The number of S_{ab} cells increases with Δt and PW_b. D) The number of S_b cells decreases with Δt but increases with PW_b.

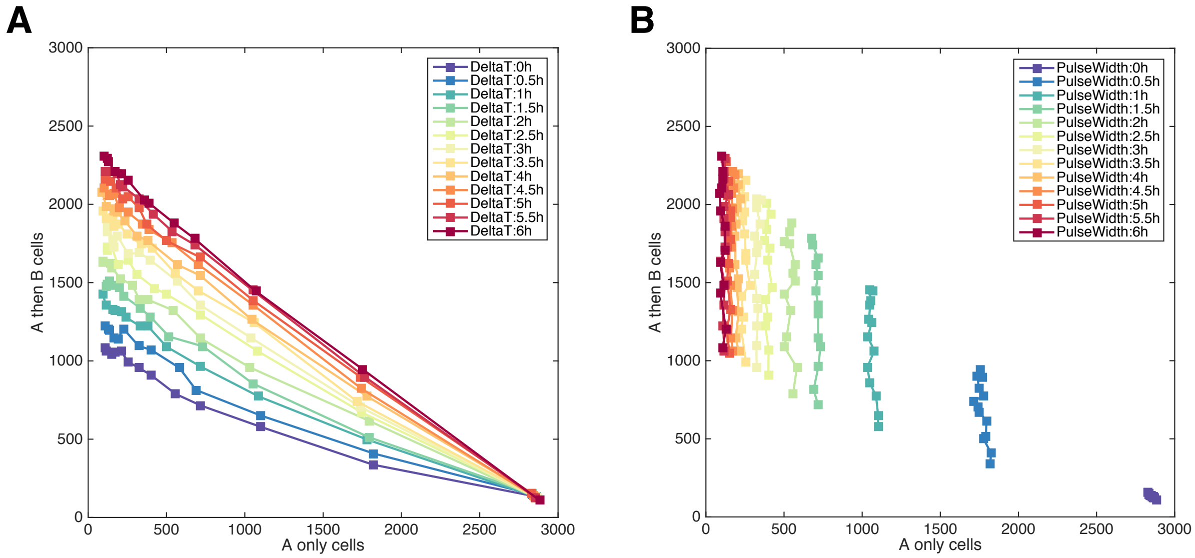

Figure S17: Unique populations for different combinations of Δt and PW_b (Fig.7). Each point represents a simulation with 3000 cells. A) Lines represent increasing Δt values. B) Lines represent increasing PW_b values.

7 Deducing inducer pulse width: experimental

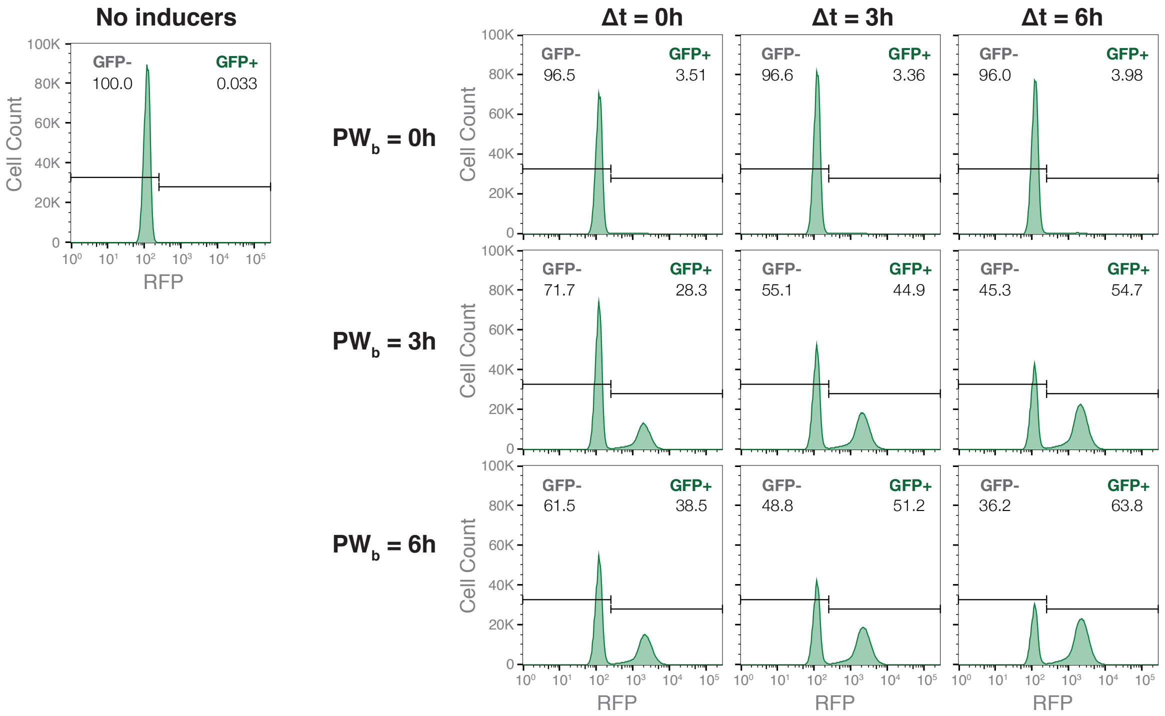

Figure S18: Flow cytometry data for Figure 7BC. GFP histograms, selected panels. ~ 1 million cells were measured for each population.

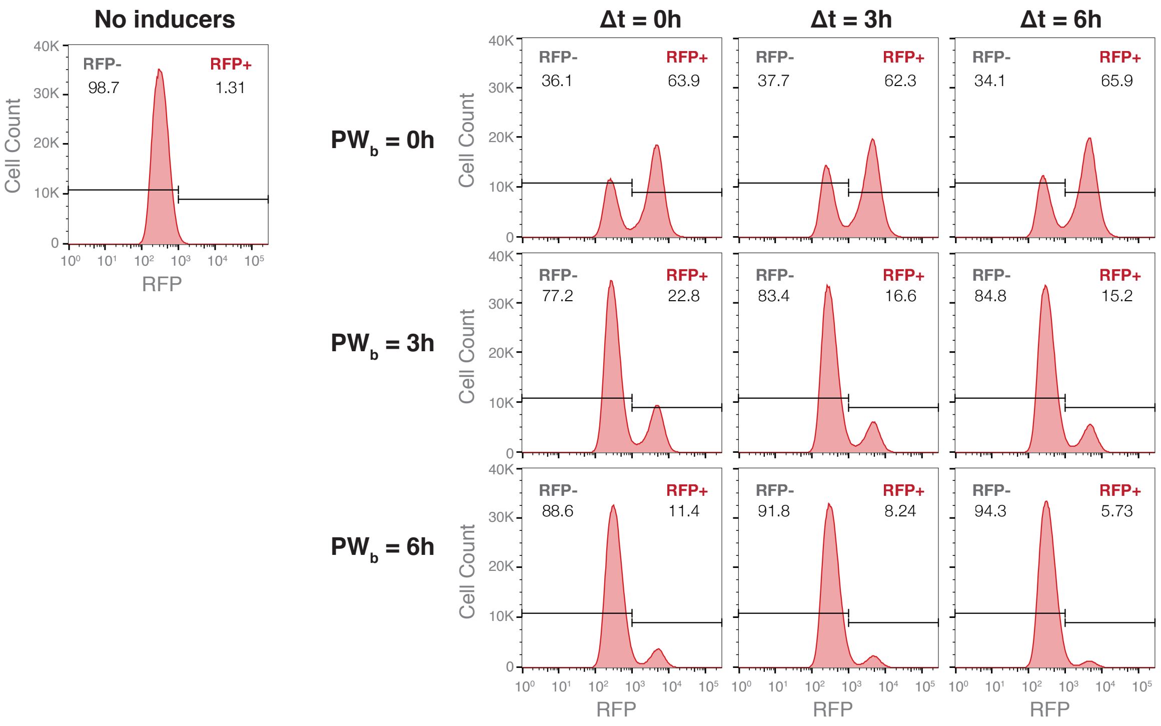

Figure S19: Flow cytometry data for Figure 7BC. RFP histograms, selected panels. $\sim$ 1 million cells were measured for each population.

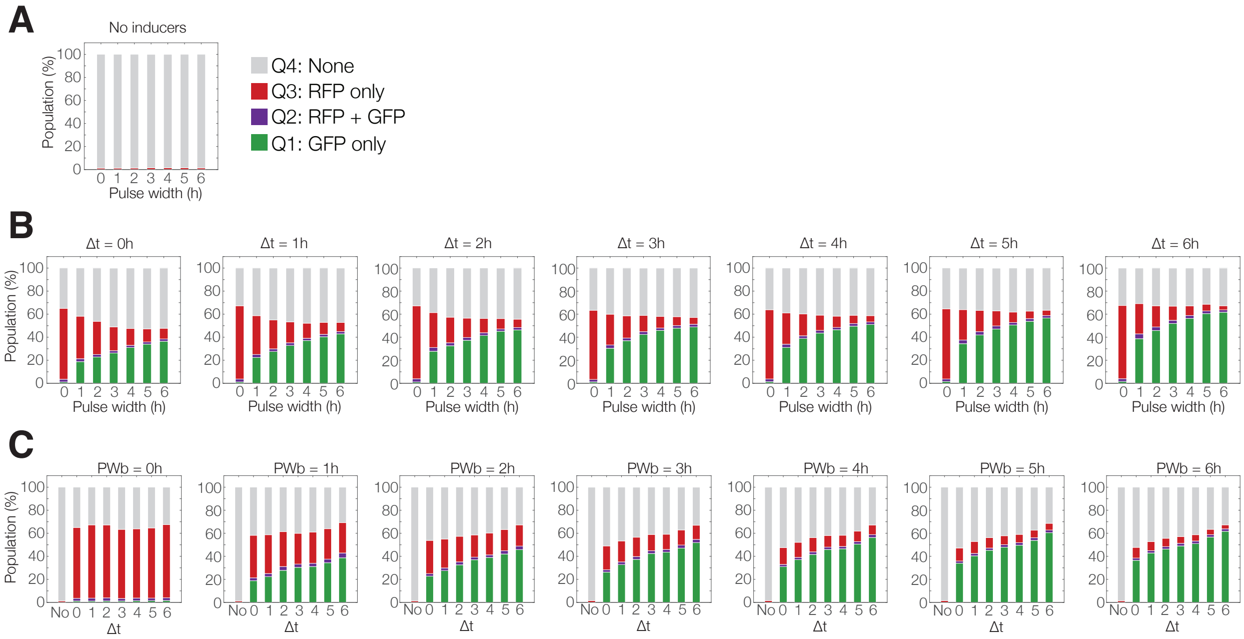

Figure S20: Population quadrants for Figure 7BC. $\sim 10^6$ cells were measured for each population. Populations are split into quadrants Q1 (GFP only, S_{ab}), Q2 (GFP + RFP, S_{ab}), Q3 (RFP only, S_a), and Q4 (non-fluorescent, $S_o + S_b$.) The Q2 population is $< 3\%$ for all conditions. A) Cultures that were incubated without any inducer exposure remained non-fluorescent. B) Population distributions as they changed with increasing PW_b. Individual subplots (left to right) are increasing Δt. C) Population distributions as they changed with increasing Δt. Individual subplots (left to right) are increasing PW_b.

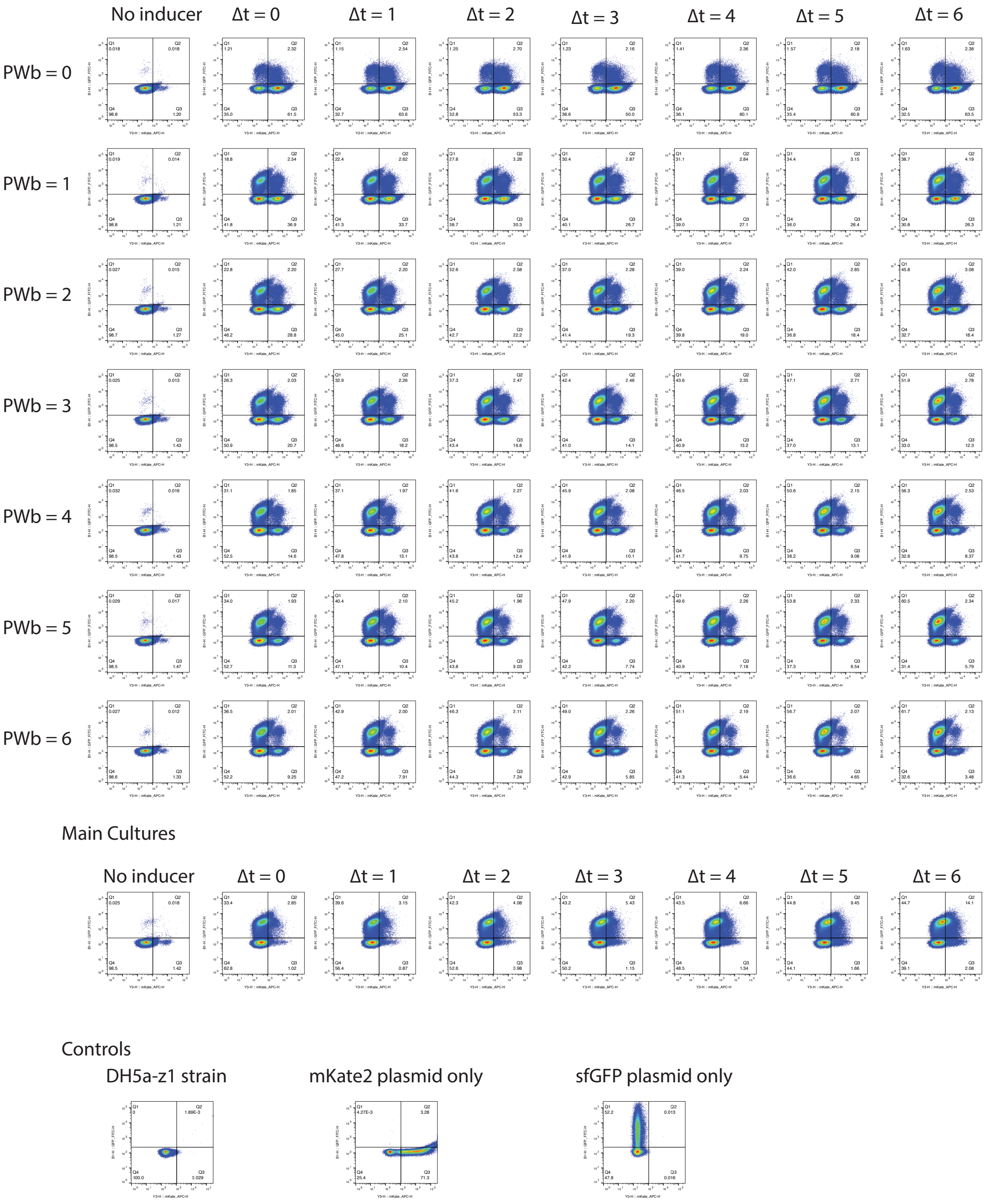

Figure S21: Complete flow cytometry data for Figure 7BC, RFP vs GFP. ∼ 1 million cells per population.

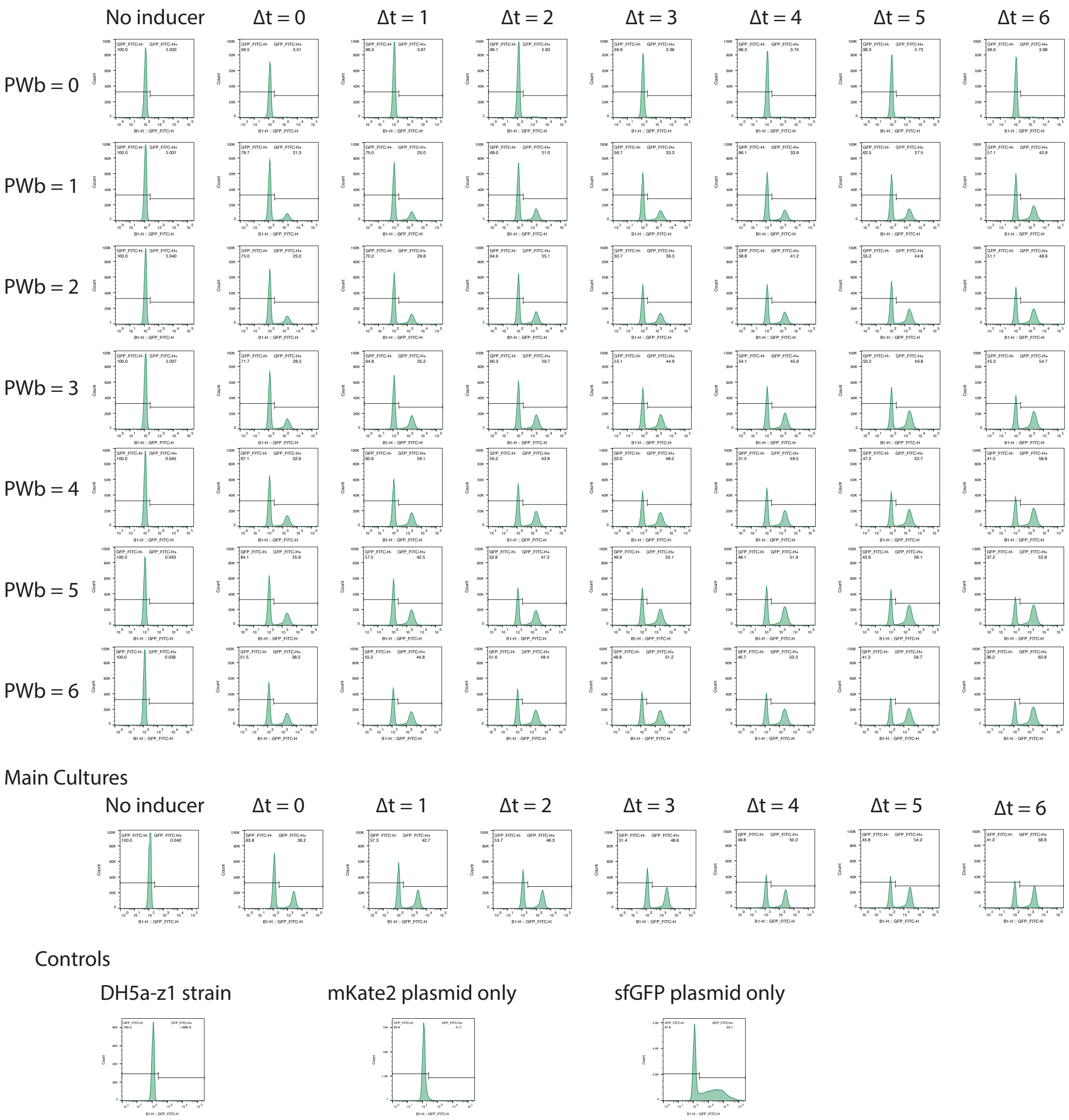

Figure S22: Complete flow cytometry data for Figure 7BC, GFP histograms. ~ 1 million cells per population.

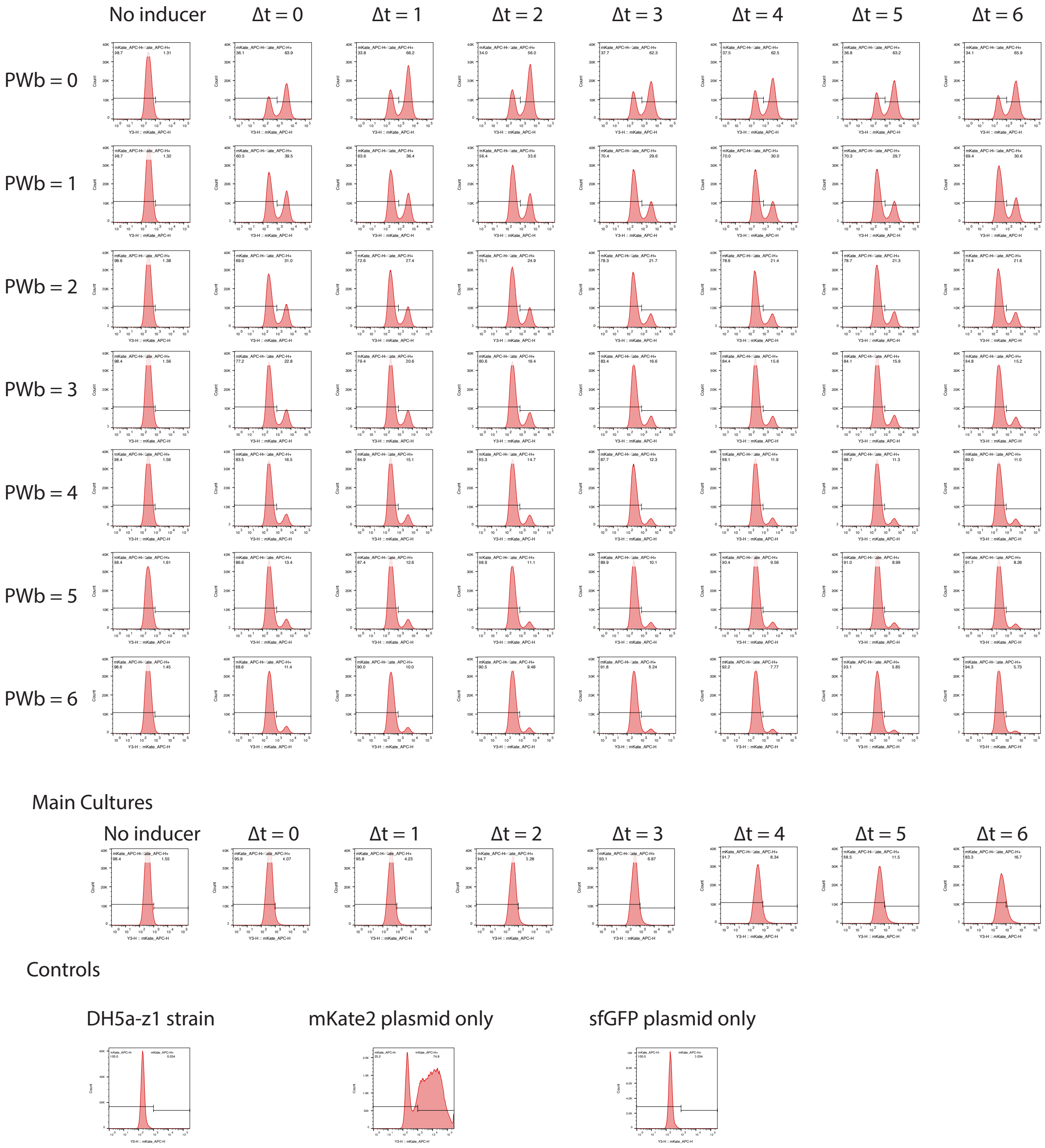

Figure S23: Complete flow cytometry data for Figure 7BC, RFP histograms. ~ 1 million cells per population.

8 Single colony analysis of pulse modulated populations

Since a significant fraction of all experimental populations from pulse experiments consisted of at least 30% non-fluorescent cells, we wanted to determine whether these colonies were S_o or S_b state cells.

Five experimental populations from the same experimental cultures as Fig. 7BC were diluted 1:10,000 and plated onto LB agar plates with no inducers (Figure S24A). We selected populations from the corners of the experimental matrix to get the widest range of results ($\Delta t = 0, 6$, $PW_b = 0, 6$). 60 ± 10 individual colonies were re-streaked onto a new agar plate with no inducers (Figure S24B). The number of RFP (S_a), green(S_{ab}), and non-fluorescent (S_o, S_b) colonies were counted. In Figure S24C, we see that population distributions from single cell counts closely matched overall flow cytometry data for entire population.

We then chose the first eight non-fluorescent colonies from each population for detailed analysis (Figure S24D). We used colony PCR to amplify the genomically-integrated DNA memory cassette from each colony (S_o,S_a,S_{ab} = 404bp, S_b = 220bp). We also included controls from the original strain (S_o), a highly RFP fluorescent colony (S_a) and a highly GFP fluorescent colony (S_{ab}). We then purified each PCR-amplified product and sequence confirmed all products (Sequencing primers, ED_seq_1F/ED_seq_1R). The eight non-fluorescent colonies were also re-streaked on LB agar + 0.01% arabinose plate to separate S_o versus S_b cells (Figure S24E). When exposed to fresh arabinose, only S_o state cells should turn red. The results from re-streaking onto inducer **a** matched PCR and sequencing results exactly.

Using S_o:S_b ratios derived from colony counts (Figure S24F), we revised the original non-fluorescent distributions shown in Figure S24C. Surprisingly, our random sample for the *no inducer* population revealed no leaky expression, though flow analysis revealed about 1–2% leaky fluorescent expression. For the $PW_b = 0$h populations, these populations never encountered inducer **b**, and so have similar S_a population fractions. Of the remaining cells for the two $PW_b = 0$h cases, we see some intB leaky expression, resulting in non-zero S_{ab} and S_b fractions for both. While it is not surprising that higher exposure to inducer **b** ($PW_b = 6$h) would result in mostly S_b cells, it was surprising that some fraction of S_o persisted over the entire 40 hour experiment. We conclude that the integrase controller plasmid has minimal leaky expression, and that over-representation of non-fluorescent states is likely due to a growth advantage over fluorescent states. Furthermore, these data show that overall integrase flipping ($S_a + S_{ab} + S_b$) is about 90% efficient with about 10% persistent S_o population which can be utilized for future responses.

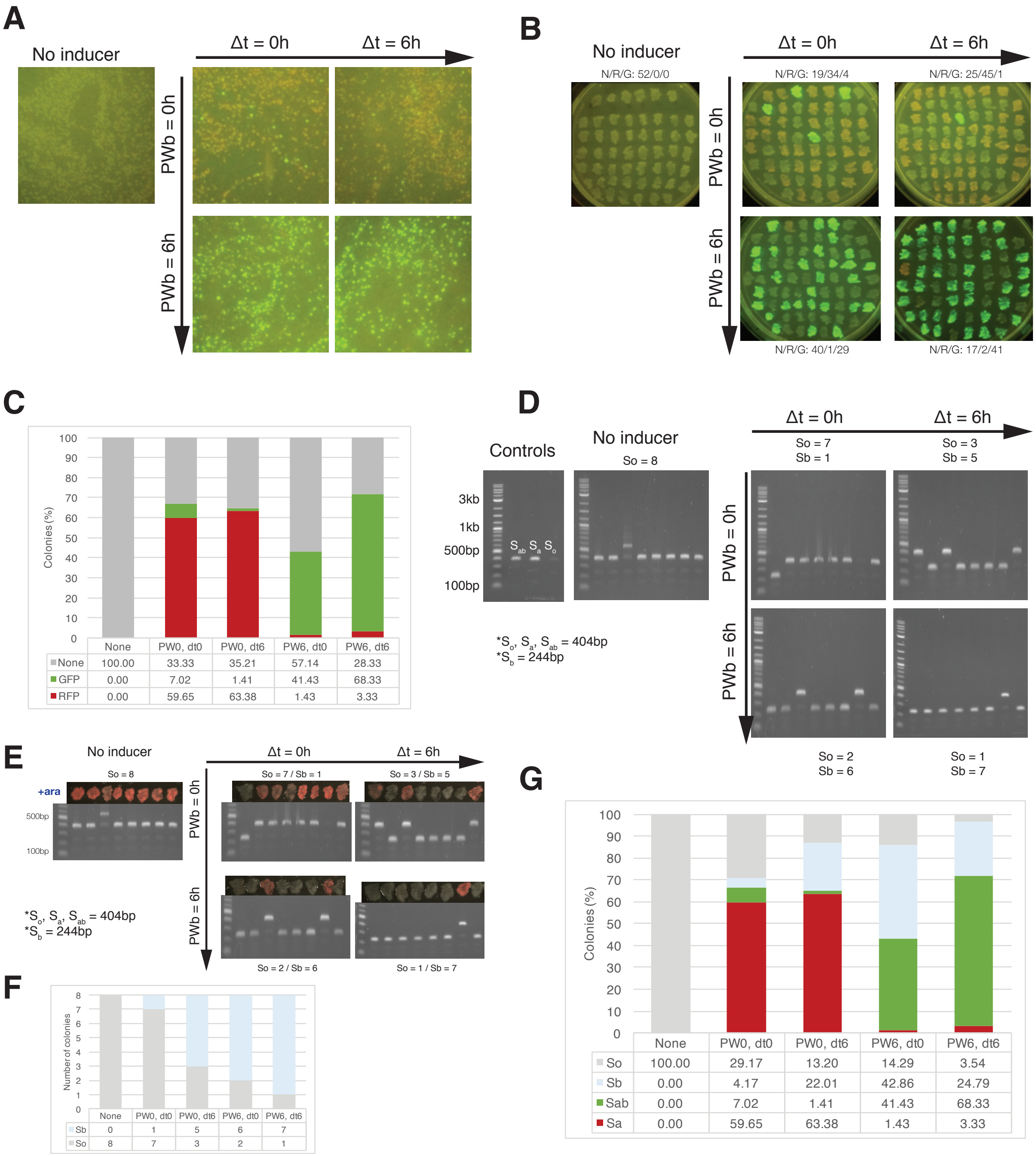

Figure S24: Single colony analysis of pulse modulated populations to determine genetic state. A) Five experimental populations from the same experimental cultures as Fig. 7BC were diluted 1:10,000 and plated onto LB agar plates with no inducers. B) 60 ± 10 individual colonies were re-streaked onto a new agar plate with no inducers. The number of RFP (S_a), green(S_{ab}), and non-fluorescent (S_o, S_b) colonies were counted. C) Population distributions from single cell counts closely matched overall flow cytometry data for entire population. D) We used colony PCR to amplify the genomically-integrated DNA memory cassette from 8 non-fluorescent colonies for each population (S_o,S_a,S_{ab} = 404bp, S_b = 220bp). Controls are from the original strain (S_o), a highly RFP fluorescent colony (S_a) and a highly GFP fluorescent colony (S_{ab}). E) The 8 non-fluorescent colonies were also re-streaked on LB agar + 0.01% ara plate to test whether only S_o state cells would turn red. Colonies matched PCR and sequencing results exactly. F) Colony counts of S_o versus S_b cells for the non-fluorescent fraction of each population. G) Revised distributions based on S_o versus S_b population ratios derived from panel F.

9 Model exploration of S_a dependence of Δt

Though our model predicted complete independence of S_a state from Δt separation times (Appendix Figure S25A), our experimental outcome showed a small linear dependence (Figure 7B,top), where lower Δt values resulted in higher S_a population fractions.

This dependence on Δt resulted in a right-to-left slant in RFP population fractions for any given PW_b value that was not predicted by our model (Figure 7A versus 7C). Upon examination of our model, we believe this is the result of unequal reaction rates during the $\mathrm{S_o} \xrightarrow{\alpha_1} \mathrm{S_b}$ transition compared to $\mathrm{S_a} \xrightarrow{\alpha_3} \mathrm{S_{ab}}$. In our model we had assumed that these rates were equal, since both are mediated by intB:

$$\alpha_1 = k_{\mathrm{flip}B} f(\mathrm{Int_B}), \tag{2}$$

$$\alpha_3 = k_{\mathrm{flip}B} f(\mathrm{Int_B}), \tag{3}$$

where $f(\mathrm{Int_B})$ is the tetramerization term:

$$f(\mathrm{Int_B}) := k_{\mathrm{flip}B} \left(\frac{\mathrm{Int_B}(\mathrm{Int_B}{-}1)(\mathrm{Int_B}{-}2)(\mathrm{Int_B}{-}3)}{K_{\mathrm{d}B}^4 + K_{\mathrm{d}B}^3 \mathrm{Int_B} + K_{\mathrm{d}B}^2 \mathrm{Int_B}(\mathrm{Int_B}{-}1) + K_{\mathrm{d}B} \mathrm{Int_B}(\mathrm{Int_B}{-}1)(\mathrm{Int_B}{-}2) + \mathrm{Int_B}(\mathrm{Int_B}{-}1)(\mathrm{Int_B}{-}2)(\mathrm{Int_B}{-}3)} \right) \tag{4}$$

where $\mathrm{Int_B}$ is integrase B concentration, $K_{\mathrm{d}B}$ is the dissociation constant, and $k_{\mathrm{flip}B}$ is the rate of flipping if the tetramer is formed.

We had made this assumption because the DNA attachment sites attB and attP are the same for both transitions, and so binding kinetics should be the same. Changing other parameters such as $K_{\mathrm{d}B}$ or $K_{\mathrm{flip}B}$ did not regenerate slanting behavior since these parameters were universal for both α_1 and α_3. However, $\mathrm{S_o} \longrightarrow \mathrm{S_b}$ is an excision reaction rather than recombination, and so the physical looping of the DNA could have different kinetics (Appendix Figure S25D).

Only when we consider the excision reaction to be slower than the other two recombination reactions,

$$\alpha_1 < \alpha_2 = \alpha_3$$

were we able to see this effect of RFP drifting with increasing Δt.

In Appendix Figure S25E and F, simulation results for $\alpha_1 = 0.6\alpha_2 = 0.6\alpha_3$ show the separation of S_a curve by Δt, and nonlinear RFP with increasing Δt in the RFP vs GFP plot. When the transition rate is decreased even more ($\alpha_1 = 0.4\alpha_2 = 0.6\alpha_3$, Appendix Figure S25G, H), the slant increases even more.

Intuitively, the reason slower $S_o \rightarrow S_b$ transition rates would cause this effect is because at lower separation times the dominating cell state is S_o, and so the predominant reactions are $\mathrm{S_o} \xrightarrow{\alpha_1} \mathrm{S_b}$ versus $\mathrm{S_o} \xrightarrow{\alpha_2} \mathrm{S_a}$. In the case of equal reaction rates, 50% goes to S_b, and 50% goes to S_a. $\mathrm{S_a} \xrightarrow{\alpha_3} \mathrm{S_{ab}}$ can only occur after S_a cells appear, and so cannot occur until after some delay. If $\alpha_1 < \alpha_2$, however, then the population split will be unequal as S_o cells are more likely to transition to S_a over S_b.

For large Δt, the dominating cell state is S_a, and so the predominant reactions are $\mathrm{S_a} \xrightarrow{\alpha_3} \mathrm{S_{ab}}$. In this case, few S_o remain, so α_1 and α_2 become less relevant as α_3 converts S_a cells into S_{ab} in a pulse width dependent manner.

So, if we consider the $\mathrm{S_a} \xrightarrow{\alpha_3} \mathrm{S_{ab}}$ conversion rate to be the baseline, then $\mathrm{S_o} \xrightarrow{\alpha_2} \mathrm{S_a}$ is generating a *higher* proportion of S_a cells than predicted at low Δt because $\alpha_2 > \alpha 1$.

Uneven transition rates are not unsurprising for experimental systems, however, changing PW_b is still the dominating determinant of cell fractions. When designing future systems it may be relevant characterize switching rates. Despite unequal intB transition rates however, each combination of PW_b and Δt still maps to unique (S_a, S_{ab}) fractional coordinates, even though S_a values are not unique for higher PW_b.

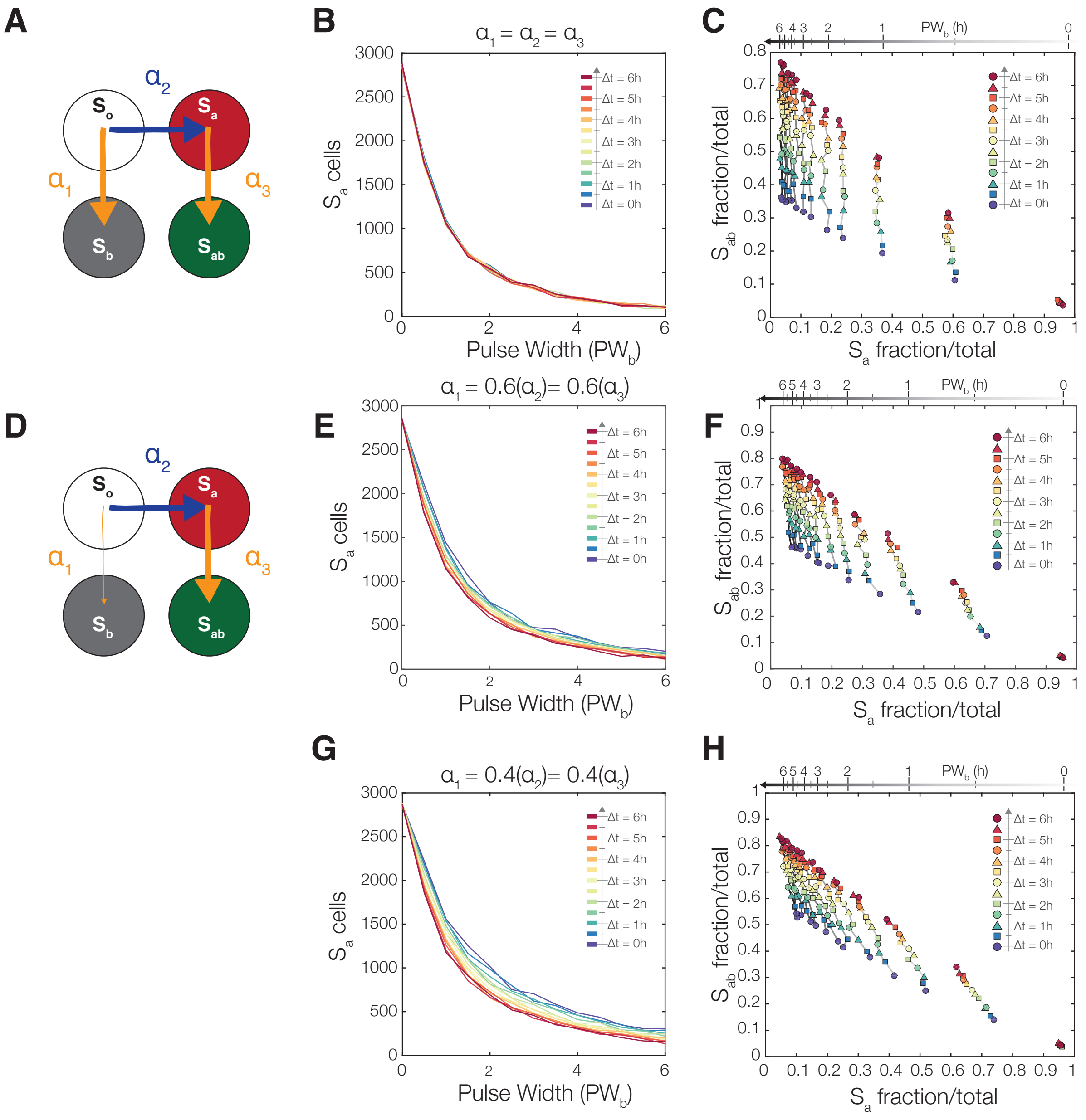

Figure S25: Simulations with unequal intB transition rates. A) Initial model parameters assume equal transition probabilities for α_1, α_2, and α_3. B) Model simulations showing total independence of S_a from Δt values. C) S_a versus S_b populations with constant S_a fractions for any given PW_b. D) We hypothesized that the excision reaction from $S_o \rightarrow S_b$ maybe be slower than $S_a \rightarrow S_{ab}$. E) S_a cell count as a function of pulse width (PW_b) with $\alpha_1 = 0.6\alpha_2 = 0.6\alpha_3$. Δt curves no longer completely overlap and low Δt values result in higher S_a fractions. F) S_a fraction versus S_{ab} fraction shows right to left slanting behavior observed in experimental results (Figure 7B,7C). G) S_a cell count as a function of pulse width (PW_b) with even slower $S_o \rightarrow S_b$ transition rate ($\alpha_1 = 0.4\alpha_2 = 0.6\alpha_3$). H) S_a fraction versus S_{ab} fraction shows right to left slanting behavior.

10 Practical use and calibration

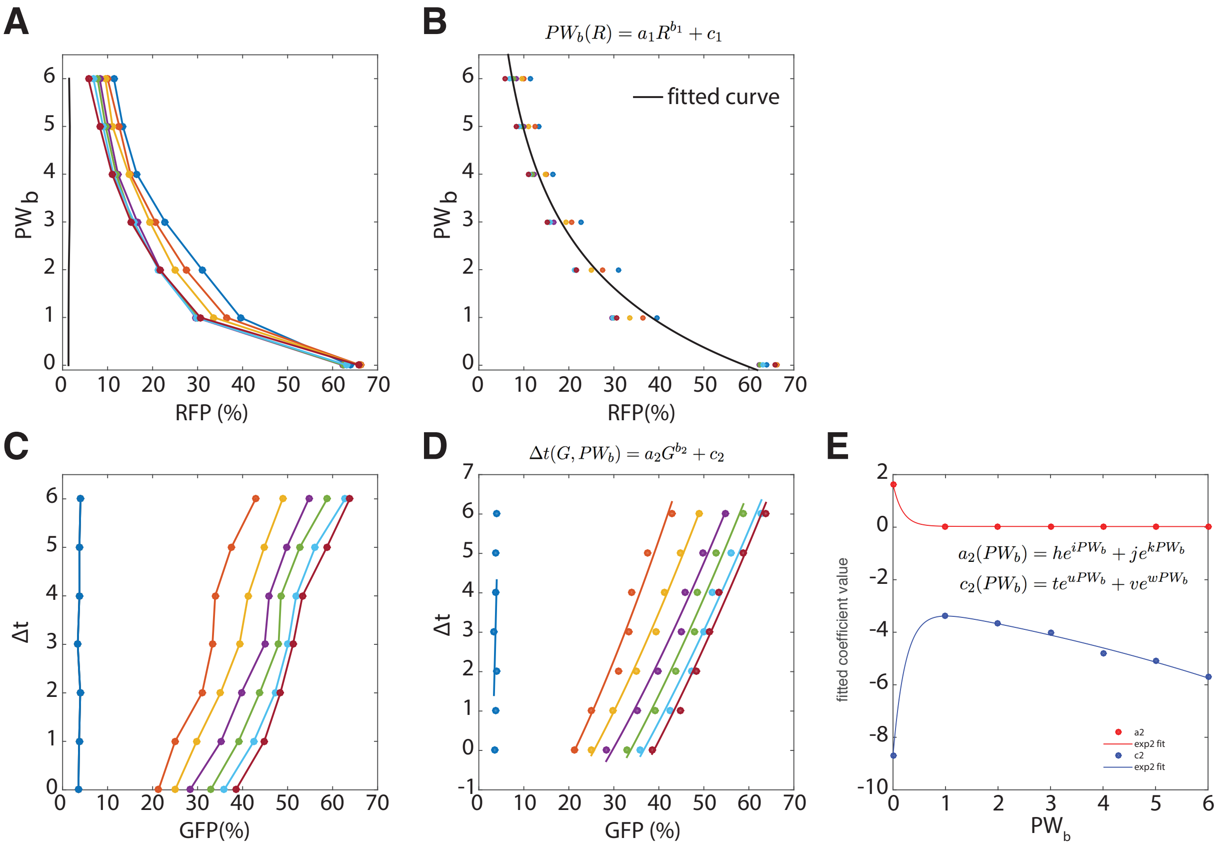

F

Parameter	Fitted Value
a_1	24.3
b_1	-0.258
c_1	-8.483
b_2	1.5
h	1.608
i	-5.587
j	0.029
k	-0.046
t	-5.742
u	-4.115
v	-2.944
w	0.1113

Figure S26: Fitting experimental data for PW_b, Δt. A) RFP population fractions from experimental data plotted with known pulse widths on the y-axis. B) Curve fit to determine PW_b dependence on RFP. A power fit (general form $PW_b(R) = a_1R^{b_1} + c_1$) to the data generates parameters $a_1 = 24.3, b_1 = -0.25, c_1 = -8.4$. C) GFP measurements from experimental data plotted with known Δt values. D) Curve fits to determine Δt dependence on GFP and RFP population fractions. Data from each value of PW_b is fitted to a different curve of general form $\Delta t(G, PW_b) = a_2G^{b_2} + c_2$. Parameter b_2 was separately fit to be 1.5. Parameters a_2 and c_2 are functions of PW_b. E) Parameters a_2 and c_2 are then fitted to their own exponential curves (general forms $a_2(PW_b) = he^{iPW_b} + je^{kPW_b}$ and $c_2(PW_b) = te^{uPW_b} + ve^{wPW_b}$) to determine dependence on PW_b. Fitted parameters are: h = 1.6, i = -5.5, j = 0.029, k = -0.046, t = -5.7, u = -4.1, v = -2.9, w = 0.1. F) A table of all the fitted parameters.

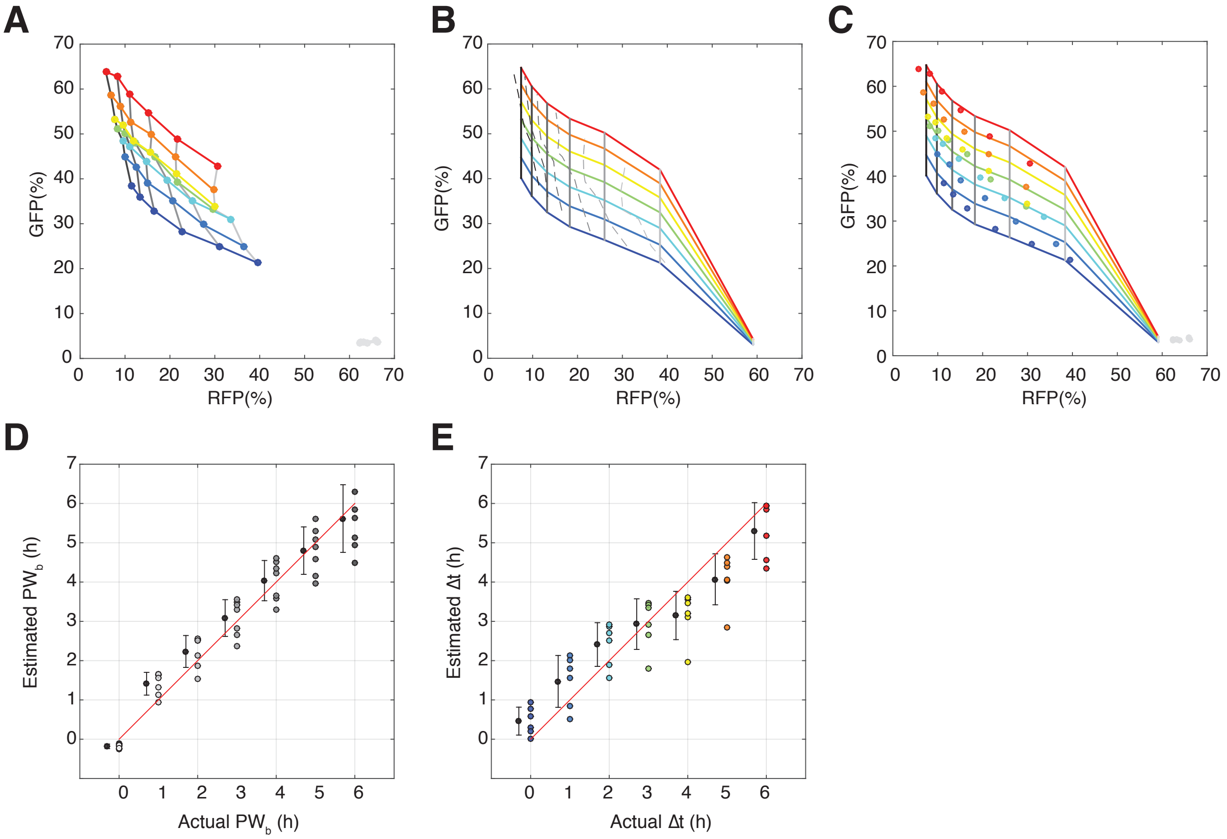

Figure S27: Resolution for determining PW_b, Δt from population distributions. A) Connected experimental values create a mesh. Area between meshlines represent the accuracy with which values of PW_b and Δt can be determined from experimentally derived RFP and GFP population fractions. RFP fractions for pulse widths from 0 – 6 hours are well separated but with decreasing resolution as PW_b increases. GFP fraction is dependent on both RFP and GFP and is also well separated, with the exception of Δt = 2, 3 hours. B) Mesh generated from the curve fits for $PW_b(R), \Delta t(G)$ discussed in Figure S26. Experimental values are colored by Δt value to show fit. C) Mesh generated from the curve fits for $PW_b(R), \Delta t(G)$ discussed in Figure S26. Experimental values are colored by PW_b value to show fit. D) Estimated PW_b values were generated from experimental RFP population fraction (%) using the fitted equation for $PW_b(R)$. The estimated values were plotted against the actual PW_b values of the experiment. The vertical gray bars show approximate spread in estimated values, the numbers above the bars indicate length of the bars (in hours). The variance in estimated values increases with higher PW_b. If there is no pulse, the resolution with which we can deduce that based on fluorescence is ± 0.25 hours. If the pulse width is 3 hours or greater, our prediction capabilities decrease to a window of ± 1 hour. For each actual PW_b values, estimated PW_b averages with $\pm$ 1 standard deviation are slightly offset on the x-axis for better comparison. E) Estimated versus actual values for Δt. Estimated Δt values are generated using the fitted curve for $\Delta t(G, PW_b)$. Variance in Δt predictions is more consistent than that for PW_b, with a resolution of ± 0.25 hours for actual Δt from 0 – 3 hours, and an estimation window of ± 0.5 hours for actual values between 4 – 6 hours. For each actual Δt values, predicted Δt averages with $\pm$ 1 standard deviation are slightly offset on the x-axis for better comparison.

11 Fitting equations and reference tables

Fitting of curves was done with experimental data from Figure 7C using the MATLAB curve fitting toolbox. Curves for $PW_b(R)$ and $\Delta t(G, PW_b)$ were fit to two-term power functions. Curves for the Δt coefficients $a_2(PW_b)$ and $c_2(PW_b)$ were fit to two-term exponential functions.

$$R = \text{RFP population (\%)} \tag{5}$$

$$G = \text{GFP population (\%)} \tag{6}$$

$$PW_b(R) = a_1 R^{b_1} + c_1 \tag{7}$$

$$\Delta t(G, PW_b) = a_2 G^{b_2} + c_2 \tag{8}$$

$$a_2(PW_b) = h e^{iPW_b} + j e^{kPW_b} \tag{9}$$

$$c_2(PW_b) = t e^{uPW_b} + v e^{wPW_b} \tag{10}$$

Parameter	Fitted value
a_1	24.3
b_1	-0.258
c_1	-8.483
b_2	1.5
h	1.608
i	-5.587
j	0.029
k	-0.046
t	-5.742
u	-4.115
v	-2.944
w	0.1113

Table S3: Fitted parameters for $PW_b(R)$, $\Delta t(G, PW_b)$

Δt **Prediction table**							
	GFP						
RFP	**0%**	**10%**	**20%**	**30%**	**40%**	**50%**	**60%**
**0%**	–	–	–	–	–	–	–
**1%**	0	0	0	0	0	0	0
**10%**	0	0	0	0	0.7	3.0	5.6
**20%**	0	0	0	0.2	2.5	5.0	7.9
**30%**	0	0	0	0.9	3.3	6.0	9.1
**40%**	0	0	0	2.9	6.3	10.2	14.5
**50%**	0	2.9	16.1	33.1	53.3	76.2	101.6
**60%**	0	52.6	∞	∞	∞	∞	∞

PW_b **Prediction table**							
	GFP						
RFP	**0%**	**10%**	**20%**	**30%**	**40%**	**50%**	**60%**
**0%**	∞	∞	∞	∞	∞	∞	∞
**1%**	15.8	15.8	15.8	15.8	15.8	15.8	15.8
**10%**	4.9	4.9	4.9	4.9	4.9	4.9	4.9
**20%**	2.7	2.7	2.7	2.7	2.7	2.7	2.7
**30%**	1.6	1.6	1.6	1.6	1.6	1.6	1.6
**40%**	0.9	0.9	0.9	0.9	0.9	0.9	0.9
**50%**	0.4	0.4	0.4	0.4	0.4	0.4	0.4
**60%**	0	0	0	0	0	0	0

Table S4: Generated table of PW_b and Δt based on fitted curves. RFP and GFP are population fractions. Use of this system for event detection requires calibration of the system first in the lab by running experimental conditions for PW_b and Δt from 0 to 6 hours, fitting for the appropriate parameters, and generation of a similar table prior to deployment in the "field".

12 Derivation: A Markov model for integrase-based temporal logic gates

12.1 Mathematical model

The state of a single cell is defined by the DNA state and the copy numbers of integrases A and B. We denote the state of a single cell by $(\mathrm{DNA}, \mathrm{IntA}, \mathrm{IntB})$, where

$$\mathrm{DNA} \in \mathcal{S} := \{S_o, S_a, S_b, S_{ab}\}, \;\; \mathrm{IntA} \in \{0, 1, 2, \cdots\}, \;\; \mathrm{IntB} \in \{0, 1, 2, \cdots\}. \tag{11}$$

For example, if the DNA state of a cell is S_a and there are $n_A := (\mathrm{IntA})$ copies of integrase A and $n_B := (\mathrm{IntB})$ copies of integrase B, the state of the cell is (S_a, n_A, n_B). In order to capture the stochastic nature of the reactions inside a single cell, we model the temporal dynamics of the cell state $(\mathrm{DNA}, \mathrm{IntA}, \mathrm{IntB})$ using a continuous-time Markov process over the state space

$$\Omega := \mathcal{S} \times \{0, 1, 2, \cdots\} \times \{0, 1, 2, \cdots\}. \tag{12}$$

Table S5 illustrates the transition rule and the rate of transitions between states, where

$$\gamma_A(t) := \begin{cases} k_{\mathrm{prodA}} + k_{\mathrm{leakA}}, & \text{if inducer } \mathbf{a} \text{ exists} \\ k_{\mathrm{leakA}} & \text{otherwise,} \end{cases} \tag{13}$$

$$\gamma_B(t) := \begin{cases} k_{\mathrm{prodB}} + k_{\mathrm{leakB}}, & \text{if inducer } \mathbf{b} \text{ exists} \\ k_{\mathrm{leakB}} & \text{otherwise.} \end{cases} \tag{14}$$

and

$$\alpha_i(n_*) := k_{\mathrm{flip}*} \frac{n_*(n_*-1)(n_*-2)(n_*-3)}{K_{\mathrm{d}*}^4 + K_{\mathrm{d}*}^3 n_* + K_{\mathrm{d}*}^2 n_*(n_*-1) + K_{\mathrm{d}*} n_*(n_*-1)(n_*-2) + n_*(n_*-1)(n_*-2)(n_*-3)} \tag{15}$$

for $K_{\mathrm{d}*}$ is the dissociation constant, $i = 1, 2, 3$ and $* = A, B$ (Full derivation, Appendix Section 12.2).

Table S5: Markov transitions of the states

Description	From	To	Intensity
From S_o to S_b	(S_o, n_A, n_B)	(S_b, n_A, n_B)	$\alpha_1(n_B)$
From S_o to S_a	(S_o, n_A, n_B)	(S_a, n_A, n_B)	$\alpha_2(n_A)$
From S_a to S_{ab}	(S_a, n_A, n_B)	(S_{ab}, n_A, n_B)	$\alpha_3(n_B)$
Production of IntA	(S_i, n_A, n_B)	$(S_i, n_A + 1, n_B)$	$\gamma_A(t)$
Degradation of IntA	(S_i, n_A, n_B)	$(S_i, n_A - 1, n_B)$	$\delta_A := k_{\mathrm{deg}} n_A$
Production of IntB	(S_i, n_A, n_B)	$(S_i, n_A, n_B + 1)$	$\gamma_B(t)$
Degradation of IntB	(S_i, n_A, n_B)	$(S_i, n_A, n_B - 1)$	$\delta_B := k_{\mathrm{deg}} n_B$

Let $\mathbb{P}_t(S_i, n_A, n_B)$ denote the probability of a cell being a state $(\mathrm{DNA} = S_i, \mathrm{IntA} = n_A, \mathrm{IntB} = n_B)$ at time t for a given initial state $\Omega_0 \in \Omega$ at time $t = 0$. More formally,

$$\mathbb{P}_t(S_i, n_A, n_B) := \mathrm{Prob}(\mathrm{DNA} = S_i, \mathrm{IntA} = n_A, \mathrm{IntB} = n_B \mid \Omega_0, t), \tag{16}$$

where $S_i \in \mathcal{S}, n_A \in \mathbb{N}_0$ and $n_B \in \mathbb{N}_0$ with the set of all non-negative integers $\mathbb{N}_0$. The dynamics of the joint probability distribution $\mathbb{P}_t(S_i, n_A, n_B)$ can then be captured by the following (infinite dimensional) ordinary differential equations (ODEs).

$$\begin{aligned}
\frac{d}{dt}\mathbb{P}_t(S_o,n_A,n_B) =& -\left\{(\alpha_1(n_B)+\alpha_2(n_A)) + (k_{\text{deg}}n_A+\gamma_A(t)) + (k_{\text{deg}}n_B+\gamma_B(t))\right\}\mathbb{P}_t(S_o,n_A,n_B)\\
&+\gamma_A(t)\mathbb{P}_t(S_o,n_A-1,n_B)+\gamma_B(t)\mathbb{P}_t(S_o,n_A,n_B-1)\\
&+k_{\text{deg}}(n_A+1)\mathbb{P}_t(S_o,n_A+1,n_B)+k_{\text{deg}}(n_B+1)\mathbb{P}_t(S_o,n_A,n_B+1) \qquad (17)\\
\frac{d}{dt}\mathbb{P}_t(S_a,n_A,n_B) =& -\left\{\alpha_3(n_B) + (k_{\text{deg}}n_A+\gamma_A(t)) + (k_{\text{deg}}n_B+\gamma_B(t))\right\}\mathbb{P}_t(S_a,n_A,n_B)\\
&+\gamma_A(t)\mathbb{P}_t(S_a,n_A-1,n_B)+\gamma_B(t)\mathbb{P}_t(S_a,n_A,n_B-1)\\
&+k_{\text{deg}}(n_A+1)\mathbb{P}_t(S_a,n_A+1,n_B)+k_{\text{deg}}(n_B+1)\mathbb{P}_t(S_a,n_A,n_B+1)\\
&+\alpha_2(n_A)\mathbb{P}_t(S_o,n_A,n_B) \qquad (18)\\
\frac{d}{dt}\mathbb{P}_t(S_b,n_A,n_B) =& -\left\{(k_{\text{deg}}n_A+\gamma_A(t)) + (k_{\text{deg}}n_B+\gamma_B(t))\right\}\mathbb{P}_t(S_b,n_A,n_B)\\
&+\gamma_A(t)\mathbb{P}_t(S_b,n_A-1,n_B)+\gamma_B(t)\mathbb{P}_t(S_b,n_A,n_B-1)\\
&+k_{\text{deg}}(n_A+1)\mathbb{P}_t(S_b,n_A+1,n_B)+k_{\text{deg}}(n_B+1)\mathbb{P}_t(S_b,n_A,n_B+1)\\
&+\alpha_1(n_B)\mathbb{P}_t(S_o,n_A,n_B) \qquad (19)\\
\frac{d}{dt}\mathbb{P}_t(S_{ab},n_A,n_B) =& -\left\{(k_{\text{deg}}n_A+\gamma_A(t)) + (k_{\text{deg}}n_B+\gamma_B(t))\right\}\mathbb{P}_t(S_{ab},n_A,n_B)\\
&+\gamma_A(t)\mathbb{P}_t(S_{ab},n_A-1,n_B)+\gamma_B(t)\mathbb{P}_t(S_{ab},n_A,n_B-1)\\
&+k_{\text{deg}}(n_A+1)\mathbb{P}_t(S_{ab},n_A+1,n_B)+k_{\text{deg}}(n_B+1)\mathbb{P}_t(S_{ab},n_A,n_B+1)\\
&+\alpha_3(n_B)\mathbb{P}_t(S_a,n_A,n_B), \qquad (20)
\end{aligned}$$

where $n_A = 0,1,2,\cdots$ and $n_B = 0,1,2,\cdots$, and we define $\mathbb{P}_t(S_i,-1,0) = \mathbb{P}_t(S_i,0,-1) = 0$ for $S_i \in \mathcal{S}$. We consider the case where $\mathbb{P}_0(S_o,0,0) = 1$ and the probability of all other states are zero at $t = 0$. In other words, all cells are at the DNA state S_o and there is no integrase at the initial time.

Since we are interested in the fraction of cells that have a certain DNA state, $\mathbb{P}_t(\text{DNA})$, rather than the joint distribution $\mathbb{P}_t(\text{DNA},\text{IntA},\text{IntB})$, we marginalize out n_A and n_B in the equations (17)–(20) by taking the sum over n_A and n_B. Specifically, for the equation (17), we have

$$\begin{aligned}
\sum_{n_A=0}^{\infty}\sum_{n_B=0}^{\infty}\frac{d}{dt}\mathbb{P}_t(S_o,n_A,n_B) &= \sum_{n_A=0}^{\infty}\sum_{n_B=0}^{\infty}\frac{d}{dt}\mathbb{P}_t(S_o)\\
&= \sum_{n_A=0}^{\infty}\sum_{n_B=0}^{\infty} -\{(\alpha_1(n_B)+\alpha_2(n_A)) + (k_{\text{deg}}n_A+\gamma_A(t))\\
&\qquad\qquad + (k_{\text{deg}}n_B+\gamma_B(t))\}\mathbb{P}_t(S_o,n_A,n_B)\\
&+\sum_{n_A=0}^{\infty}\sum_{n_B=0}^{\infty}\gamma_A(t)\mathbb{P}_t(S_o,n_A,n_B)+\sum_{n_A=0}^{\infty}\sum_{n_B=0}^{\infty}\gamma_B(t)\mathbb{P}_t(S_o,n_A,n_B)\\
&+\sum_{n_A=0}^{\infty}\sum_{n_B=0}^{\infty}k_{\text{deg}}n_A\mathbb{P}_t(S_o,n_A,n_B)+\sum_{n_A=0}^{\infty}\sum_{n_B=0}^{\infty}k_{\text{deg}}n_B\mathbb{P}_t(S_o,n_A,n_B)\\
&= \sum_{n_A=0}^{\infty}\sum_{n_B=0}^{\infty} -(\alpha_1(n_B)+\alpha_2(n_A))\mathbb{P}_t(S_o,n_A,n_B)\\
&= -(\mathbb{E}[\alpha_1(n_B)|S_o]+\mathbb{E}[\alpha_2(n_A)|S_o])\mathbb{P}_t(S_o), \qquad (21)
\end{aligned}$$

where the last equality comes from

$$\sum_{n_A=0}^{\infty}\sum_{n_B=0}^{\infty}\alpha_2(n_A)\mathbb{P}_t(S_o,n_A,n_B) = \sum_{n_A=0}^{\infty}\alpha_2(n_A)\mathbb{P}_t(S_o,n_A) = \sum_{n_A=0}^{\infty}\alpha_2(n_A)\mathbb{P}_t(n_A|S_o)\mathbb{P}_t(S_o) = \mathbb{E}[\alpha_2(n_A)|S_o]\mathbb{P}_t(S_o)$$

and the same argument for $\sum_{n_A=0}^{\infty}\sum_{n_B=0}^{\infty}\alpha_1(n_B)\mathbb{P}_t(S_o,n_A,n_B)$.

In a similar manner, we can derive the following differential equations for $\mathbb{P}_t(S_a), \mathbb{P}_t(S_b), \mathbb{P}_t(S_{ab})$ using (18), (19) and (20), respectively.

$$\frac{d}{dt}\begin{bmatrix}\mathbb{P}_t(S_o)\\ \mathbb{P}_t(S_a)\\ \mathbb{P}_t(S_b)\\ \mathbb{P}_t(S_{ab})\end{bmatrix} = \begin{bmatrix}-\mathbb{E}[\alpha_1(\mathrm{IntB})|S_o] - \mathbb{E}[\alpha_2(\mathrm{IntA})|S_o] & 0 & 0 & 0\\ \mathbb{E}[\alpha_2(\mathrm{IntA})|S_o] & -\mathbb{E}[\alpha_3(\mathrm{IntB})|S_a] & 0 & 0\\ \mathbb{E}[\alpha_1(\mathrm{IntB})|S_o] & 0 & 0 & 0\\ 0 & \mathbb{E}[\alpha_3(\mathrm{IntB})|S_a] & 0 & 0\end{bmatrix}\begin{bmatrix}\mathbb{P}_t(S_o)\\ \mathbb{P}_t(S_a)\\ \mathbb{P}_t(S_b)\\ \mathbb{P}_t(S_{ab})\end{bmatrix}. \tag{22}$$

These equations describe how the fraction of cells with each DNA state evolves over time.

12.2 Tetramerization of integrases

The serine integrases need to tetramerize prior to DNA recombination. Each DNA binding site (attB, attP) is bound by two copies of integrase monomers in an independent manner (independent binding) to form a dimer (Ghosh *et al,* 2008; Singh *et al,* 2013,2014). Once both of the binding sites on both DNA attachment sites are occupied, a dimer of the dimers (tetramer) is formed and recombination can occur. Here we derive the reaction propensity of the tetramerization process.

Let $S_i:\mathrm{Int}^i$ $(i = 1, 2, 3, 4)$ denote the DNA states where i copies of integrase molecule are bound to the DNA, and denote the copy numbers of integrase A and B by n_* $(* = A, B)$. DNA recombination occurs when the DNA state is at $S_i:\mathrm{Int}^4$, which implies the tetramerization of integrases on the DNA. Since the binding of integrases is independent binding, the transition of the DNA states can be modeled by

$$S_i \underset{k_{\mathrm{unbind}}}{\overset{k_{\mathrm{bind}}}{\rightleftharpoons}} S_i:\mathrm{Int}^1 \underset{k_{\mathrm{unbind}}}{\overset{k_{\mathrm{bind}}}{\rightleftharpoons}} S_i:\mathrm{Int}^2 \underset{k_{\mathrm{unbind}}}{\overset{k_{\mathrm{bind}}}{\rightleftharpoons}} S_i:\mathrm{Int}^3 \underset{k_{\mathrm{unbind}}}{\overset{k_{\mathrm{bind}}}{\rightleftharpoons}} S_i:\mathrm{Int}^4$$

The dynamics of the transitions of DNA states can then be modeled by the following ODE.

$$\frac{d}{dt}\mathbb{P}_t(S_i, n_*) = -k_{\mathrm{bind}}n_*\mathbb{P}_t(S_i, n_*) + k_{\mathrm{unbind}}\mathbb{P}_t(S_i:\mathrm{Int}^1, n_*) \tag{23}$$

$$\begin{aligned}\frac{d}{dt}\mathbb{P}_t(S_i:\mathrm{Int}^1, n_*-1) =& -(k_{\mathrm{bind}}(n_*-1) + k_{\mathrm{unbind}})\mathbb{P}_t(S_i:\mathrm{Int}^1, n_*-1) + k_{\mathrm{bind}}n_*\mathbb{P}_t(S_i, n_*)\\ &+ k_{\mathrm{unbind}}\mathbb{P}_t(S_i:\mathrm{Int}^2, n_*-2)\end{aligned} \tag{24}$$

$$\begin{aligned}\frac{d}{dt}\mathbb{P}_t(S_i:\mathrm{Int}^2, n_*-2) =& -(k_{\mathrm{bind}}(n_*-2) + k_{\mathrm{unbind}})\mathbb{P}_t(S_i:\mathrm{Int}^2, n_*-2) + k_{\mathrm{bind}}(n_*-1)\mathbb{P}_t(S_i:\mathrm{Int}^1, n_*-1)\\ &+ k_{\mathrm{unbind}}\mathbb{P}_t(S_i:\mathrm{Int}^3, n_*-3)\end{aligned} \tag{25}$$

$$\begin{aligned}\frac{d}{dt}\mathbb{P}_t(S_i:\mathrm{Int}^3, n_*-3) =& -(k_{\mathrm{bind}}(n_*-3) + k_{\mathrm{unbind}})\mathbb{P}_t(S_i:\mathrm{Int}^3, n_*-3) + k_{\mathrm{bind}}(n_*-2)\mathbb{P}_t(S_i:\mathrm{Int}^2, n_*-2)\\ &+ k_{\mathrm{unbind}}\mathbb{P}_t(S_i:\mathrm{Int}^4, n_*-4)\end{aligned} \tag{26}$$

$$\frac{d}{dt}\mathbb{P}_t(S_i:\mathrm{Int}^4, n_*-4) = -k_{\mathrm{unbind}}\mathbb{P}_t(S_i:\mathrm{Int}^4, n_*-4) + k_{\mathrm{bind}}(n_*-3)\mathbb{P}_t(S_i:\mathrm{Int}^3, n_*-3), \tag{27}$$

where k_{bind} and k_{unbound} are binding and unbinding rate constants, respectively.

We assume that binding and unbinding of integrase molecules to DNA equilibrate fast enough compared to the dynamics of DNA recombination, and the production and degradation of integrases, and hence the equations (23)–(27) converge to an equilibrium while n_* remain constant (equilibrium approximation). Substituting zero to the left-hand side of the ODEs (23)–(27) and solving in terms of $\mathbb{P}_t(S_i:\mathrm{Int}^4, n_*-4)$, we have

$$\mathbb{P}_t(S_i:\mathrm{Int}^4, n_*-4) = \frac{n_*(n_*-1)(n_*-2)(n_*-3)}{K_{\mathrm{d}*}^4 + K_{\mathrm{d}*}^3 n_* + K_{\mathrm{d}*}^2 n_*(n_*-1) + K_{\mathrm{d}*}n_*(n_*-1)(n_*-2) + n_*(n_*-1)(n_*-2)(n_*-3)}, \tag{28}$$

where $K_{\mathrm{d}*} := k_{\mathrm{unbind}}/k_{\mathrm{bind}}$.

This implies that the rate of DNA flipping is given by

$$k_{\mathrm{flip}*}\mathbb{P}_t(S_i:\mathrm{Int}^4, n_*-4) = k_{\mathrm{flip}*}\frac{n_*(n_*-1)(n_*-2)(n_*-3)}{K_{\mathrm{d}*}^4 + K_{\mathrm{d}*}^3 n_* + K_{\mathrm{d}*}^2 n_*(n_*-1) + K_{\mathrm{d}*}n_*(n_*-1)(n_*-2) + n_*(n_*-1)(n_*-2)(n_*-3)}.$$

12.3 DNA state S_a is independent of inducer separation time Δt

In what follows, we analyze the equation (22) to mathematically show that the fraction of cells at the DNA state S_a is independent of the inducer separation time Δt at steady state ($t \to \infty$) when there is no leaky expression of the integrase B. In other words, we will show that the steady state probability $\mathbb{P}_\infty(S_a)$ is independent of Δt when $k_{\text{leak}B} = 0$. In the last paragraph, we discuss the implications when $k_{\text{leak}B} \neq 0$.

We consider the case where the inducer A is present for $t \geq 0$ and the inducer B is present for $\Delta t \leq t \leq \Delta t + \text{PW}_b$, that is,

$$\gamma_A(t) := k_{\text{prod}A} + k_{\text{leak}A} \tag{29}$$

$$\gamma_B(t) := \begin{cases} k_{\text{prod}B}, & \Delta t \leq t \leq \Delta t + \text{PW}_b \\ 0 & \text{otherwise.} \end{cases} \tag{30}$$

It follows by adding the first two and the last two equations of the ODE (22) that

$$\frac{d}{dt}\left(\mathbb{P}_t(S_o) + \mathbb{P}_t(S_a)\right) = \left(\mathbb{E}[\alpha_1(\text{IntB})|S_o]\mathbb{P}_t(S_o) + \mathbb{E}[\alpha_3(\text{IntB})|S_a]\mathbb{P}_t(S_a)\right) \tag{31}$$

$$= -\frac{d}{dt}\left(\mathbb{P}_t(S_b) + \mathbb{P}_t(S_{ab})\right). \tag{32}$$

Since the production rate of the integrase B is $\gamma_B = 0$ when there is no leaky expression, or $k_{\text{leak}B} = 0$ (see the definition (14)), $\mathbb{E}[\alpha_1(\text{IntB})|S_o] = \mathbb{E}[\alpha_3(\text{IntB})|S_a] = 0$ for $0 \leq t \leq \Delta t$. This implies that the right-hand side of the ODE (31) is zero for $0 \leq t \leq \Delta t$, and all cells are at either of S_o or S_a state, or equivalently $\mathbb{P}_t(S_o) + \mathbb{P}_t(S_a) = 1$ for $0 \leq t \leq \Delta t$. Taking the summation over time, we have

$$\int_0^\infty \frac{d}{dt}\left(\mathbb{P}_t(S_o) + \mathbb{P}_t(S_a)\right) = \int_0^\infty \left(\mathbb{E}[\alpha_1(\text{IntB})|S_o]\mathbb{P}_t(S_o) + \mathbb{E}[\alpha_3(\text{IntB})|S_a]\mathbb{P}_t(S_a)\right) dt. \tag{33}$$

The left-hand side can be calculated as

$$\left(\mathbb{P}_\infty(S_o) - \mathbb{P}_0(S_o)\right) + \left(\mathbb{P}_\infty(S_a) - \mathbb{P}_0(S_a)\right) = -1 + \mathbb{P}_\infty(S_a), \tag{34}$$

where we used $\mathbb{P}_\infty(S_o) = 0$, which follows from the first equation of the ODE (22), and the initial conditions $\mathbb{P}_0(S_o) = 1$ and $\mathbb{P}_0(S_a) = 0$. In a similar way, it follows from the equation (32) that

$$\int_0^\infty -\frac{d}{dt}\left(\mathbb{P}_t(S_b) + \mathbb{P}_t(S_{ab})\right) dt = -\mathbb{P}_\infty(S_b) - \mathbb{P}_\infty(S_{ab}). \tag{35}$$

Thus, the steady state probability $\mathbb{P}_\infty(S_a)$ satisfies $\mathbb{P}_\infty(S_a) = 1 - (\mathbb{P}_\infty(S_b) + \mathbb{P}_\infty(S_{ab}))$.

The right-hand side of the equation (31) can be written as

$$\int_0^\infty \left(\mathbb{E}[\alpha_1(\text{IntB})|S_o]\mathbb{P}_t(S_o) + \mathbb{E}[\alpha_3(\text{IntB})|S_a]\mathbb{P}_t(S_a)\right) dt$$
$$= \sum_{n_B = \nu_B}^{\infty} \int_0^\infty \alpha_1(\text{IntB})(\mathbb{P}_t(S_o, n_B) + \mathbb{P}_t(S_a, n_B))dt,$$

where we used the relation $\alpha_1(\text{IntB}) = \alpha_3(\text{IntB})$ from the definition. Then, the following two observations allow us to show that $\mathbb{P}_t(S_o, n_B) + \mathbb{P}_t(S_a, n_B)$ is independent of Δt. First, $\mathbb{P}_{\Delta t}(\text{DNA} = S_o, \text{IntB} = 0) + \mathbb{P}_{\Delta t}(\text{DNA} = S_a, \text{IntB} = 0) = \mathbb{P}_{\Delta t}(\text{DNA} = S_o \cup S_a, \text{IntB} = 0) = 1$, since $n_B = 0$ and the DNA state of any single cells is either of S_o or S_a for $0 \leq t \leq \Delta t$ as already discussed above. Second,

$$\mathbb{P}_t(S_o, n_B|n_A) + \mathbb{P}_t(S_a, n_B|n_A) = \mathbb{P}_t(S_o \cup S_a, n_B|n_A) = \mathbb{P}_t(S_o \cup S_a, n_B) \tag{36}$$

for all $n_A, n_B = 0, 1, 2, \cdots$, since the intensity functions of all transitions $(n_A, n_B, S_o) \to (n_A, n_B \pm 1, S_o)$, $(n_A, n_B, S_a) \to (n_A, n_B \pm 1, S_a)$, $(n_A, n_B, S_o) \to (n_A, n_B, S_b)$ and $(n_A, n_B, S_a) \to (n_A, n_B, S_{ab})$ are defined

without the copy number of n_A. The Markov property then implies that $\mathbb{P}_t(S_o, n_B) + \mathbb{P}_t(S_a, n_B)$ is independent of inducer separation time Δt, since the dynamics of the probability $\mathbb{P}_t(S_o, n_B) + \mathbb{P}_t(S_a, n_B)$ is determined by a Markov process with the same initial condition $\mathbb{P}_{\Delta t}(\mathrm{DNA} = S_o \cup S_a, \mathrm{IntB} = 0)$.

When $k_{\mathrm{leak}B} \neq 0$, the probability $\mathbb{P}_{\Delta t}(\mathrm{DNA} = S_o \cup S_a, \mathrm{IntB} = 0)$ is no longer equal to 1 due to the production of IntB before induction ($0 \leq t \leq \Delta t$). Moreover, in the limit of $t \to \infty$, all cells are turned into S_b or S_{ab} state. Thus, mathematically, it holds that $\mathbb{P}_\infty(S_a) \to 0$ no matter how we take Δt and PW_b. However, if $k_{\mathrm{leak}B}$ is negligibly small compared to other kinetic constants, $k_{\mathrm{flipA}}, k_{\mathrm{flipB}}, k_{\mathrm{deg}}, \gamma_A(t)$ and $k_{\mathrm{prod}B}$, we can expect that $\mathbb{P}_{\Delta t}(\mathrm{DNA} = S_o \cup S_a, \mathrm{IntB} = 0) \simeq 1$ for the practical range of Δt, and $\mathbb{P}_t(S_a)$ is little affected by Δt for $t \geq \Delta t$. In addition, practically speaking, the measurement is taken at a sufficiently large but a finite time after the end of the induction of IntB ($t = \Delta t + PW_b$), thus $\mathbb{P}_t(S_a)$ is finite at the time of measurement. Although not mathematically rigorous, these arguments can also be confirmed with the numerical simulation result (see Figure 6E of main text).

13 List of plasmids and cell strains used

**Plasmids**		
Name	Resistance	Description
pVHed05	Cm	Controller plasmid (slightly modified Dual Recombinase Controller)
pVHed07	Kan	Integration plasmid for temporal logic gate in Phi80 site
pAH123 (Addgene 66077)	Amp (30C)	Helper plasmid needed for chromosomal integration in Phi80 site

**Cell strains**		
Name	Resistance	Description
DH5α-Z1		received from Endy lab
E. coli *pir* 2+		Necessary for cloning integration plasmids (contains the *pir* protein needed for replication of R6K origin of replication)
eVHed07	Kan/Cm	Chromosomally integrated temporal logic gate strain with integrase controller plasmid

**Sequencing primers**		
Name	Sequence	TM
ED_seq_F1	AAGCTTATGCCAACACAATT	59C (with Phusion Hotstart Flex 2x Mastermix)
ED_seq_R1	AGCTTCGTGGTTTGTCTG	59C

14 Plasmid maps

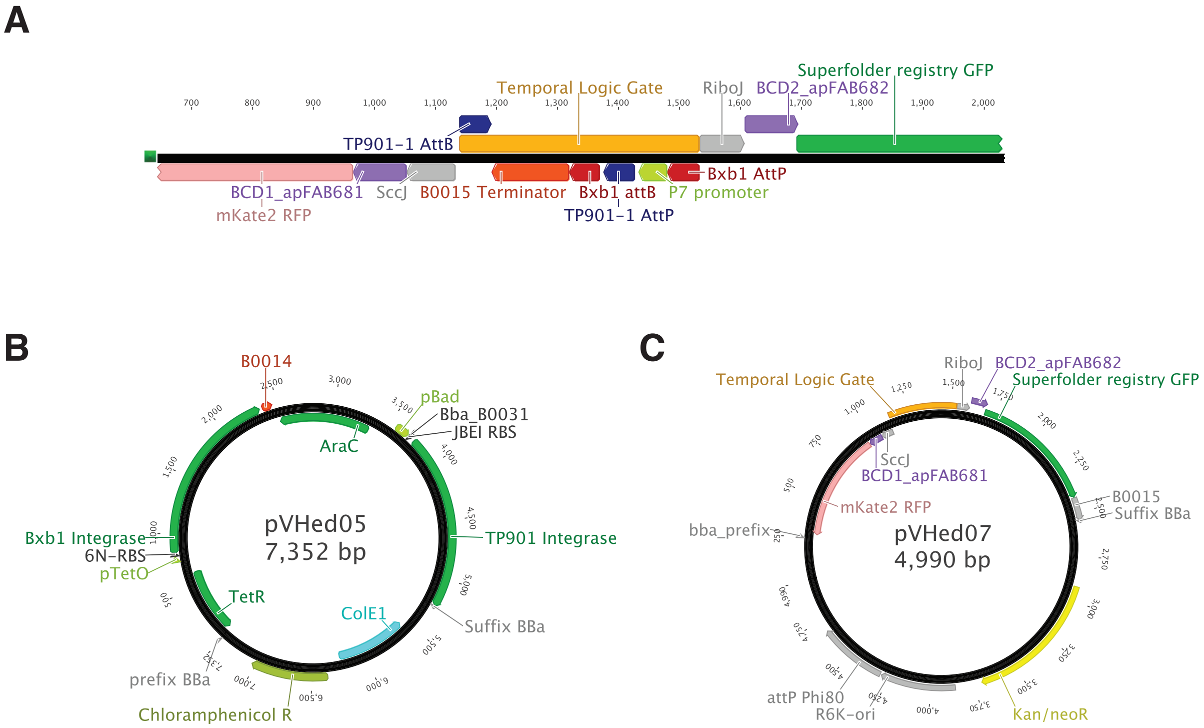

Figure S28: Plasmid maps of temporal logic gate system. A) Design of the temporal logic gate. B) Controller plasmid for integrase A (Ptet-Bxb1) and integrase B (PBAD-TP901-1)
