# Supplementary material for: A population‐based temporal logic gate for timing and recording chemical events
Source: Mol Syst Biol. 2016 May 18;12(5):869. doi: 10.15252/msb.20156663 (PMC5289221; doi:10.15252/msb.20156663)
Supplement: Supplementary file 2 — Expanded View Figures PDF [file MSB-12-869-s002.pdf]

Expanded View Figures

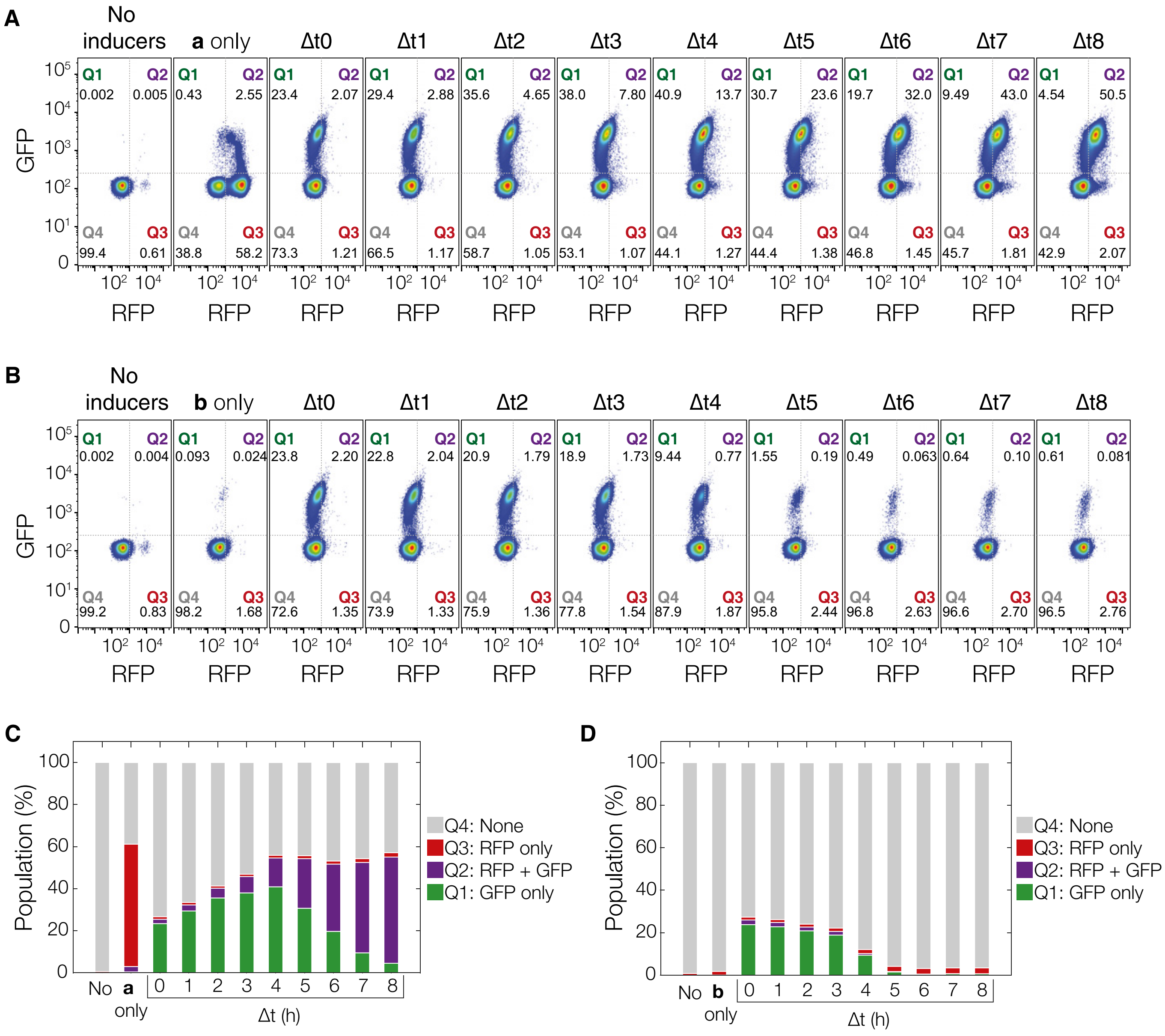

Figure EV1. Flow cytometry data for varying inducer separation time from Δt = 0 to 8 h (~10^6 cells per population).

The populations are gated by fluorescence into Q1 (S_{ab}, GFP only), Q2 (S_{ab}, RFP and GFP), Q3 (S_a, RFP only), and Q4 (S_o, S_b, non-fluorescent). There is a transitory phase (Q2) in which cells contain both GFP and RFP. This is due to slow dilution of RFP through cell division even after cells have switched to S_{ab} and begun production of GFP. Cells in Q1 + Q2 are used for the final GFP population fractions in Fig 4C.

A E_{ab} cell populations plotted by their RFP and GFP expression with increasing Δt. Leaky expression of PBAD-intA can be estimated by looking at Q3 of the *No inducers*, ***b** only* populations (~0.5–2%). Leaky expression of Ptet-intB can be estimated with Q1 + Q2 fractions of the ***a** only* population (~2–3%).

B E_{ba} populations with increasing Δt.

C Population fractions by quadrant for ***a** then **b***, E_{ab}.

D Population fractions by quadrant for ***b** then **a***, E_{ba}. Individual flow cytometry histograms can be found in Appendix Figs S8–S10.

Source data are available online for this figure.

© 2016 The Authors

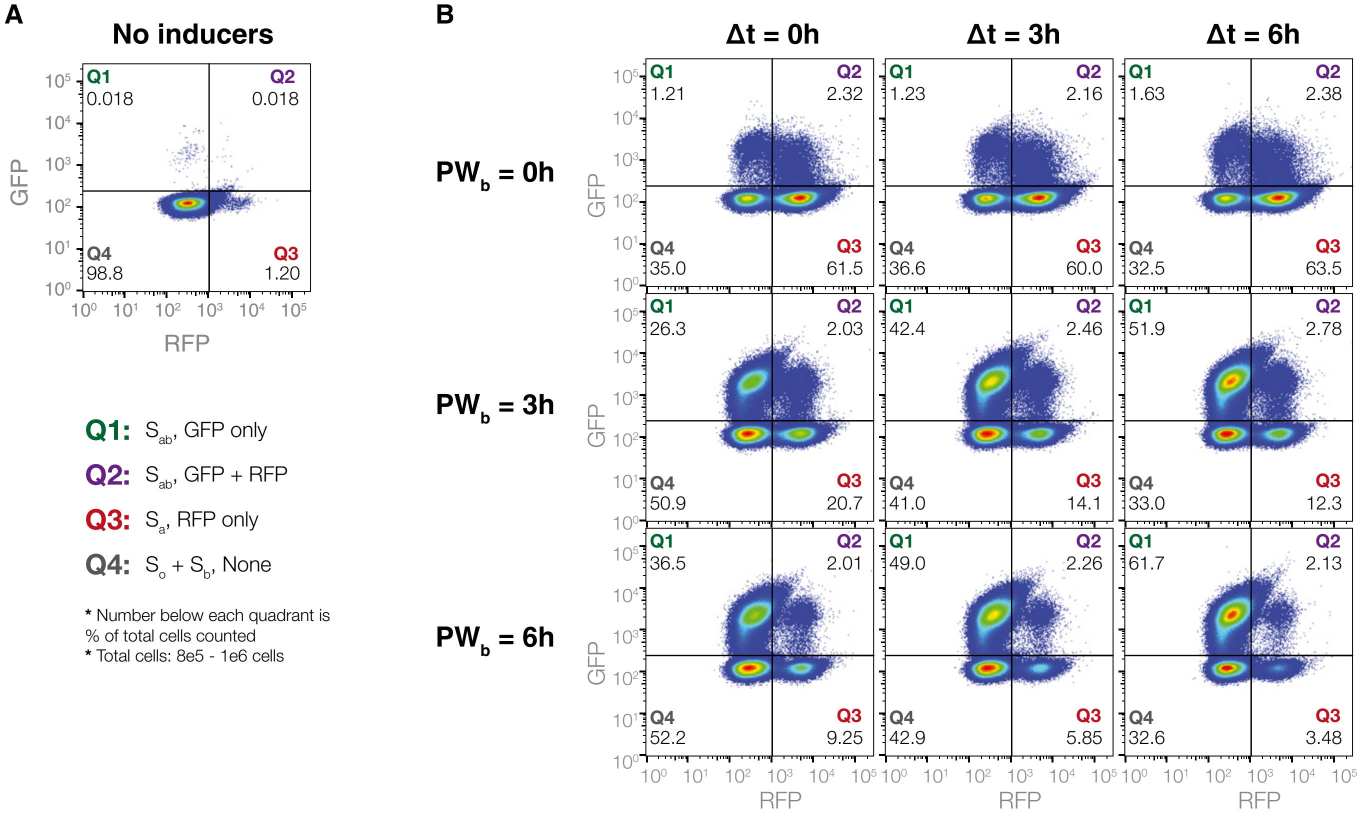

Figure EV2. Selected flow cytometry panels for Fig 7C populations.

Cell populations are gated by RFP and GFP fluorescence into quadrants Q1 (S_{ab}, GFP only), Q2 (S_{ab}, RFP and GFP), Q3 (S_a, RFP only), and Q4 (S_o, S_b, non-fluorescent). Percentage of total cells in each quadrant is shown under the quadrant label in each panel.

- A Control population not exposed to any inducers. There is minimal leaky expression (1–2%) into Q3 after 36 h of growth.
- B Populations for PW_b and Δt values of 0, 3, and 6 h. For PW_b = 0 h populations, ~60% of the cells switch to S_a (Q3), with ~3% intB leaky expression going into Q1 and Q2. As PW_b increases, the S_a fraction drops from 60% (PW_b = 0 h) to 10–20% (PW_b = 3 h) to less than 10% (PW_b = 6 h). As Δt increases, the percentage of cells in S_{ab} (Q1) increases from 20–40% (Δt = 0 h) to 40–50% (Δt = 3 h) to 50–60% (Δt = 6 h). S_a populations (Q3) also drift downwards with increasing Δt, rather than staying constant as predicted in simulation. Lower Δt results in higher S_b populations, which, combined with S_o cells, make up Q4. Critically, the percentage of the population expressing both RFP and GFP simultaneously (Q2) is always < 3%. This ensures that RFP is a reliable determinant of S_a state cells, and subsequently, of PW_b.

Source data are available online for this figure.

 © 2016 The Authors
