# Supplementary material for: A population‐based temporal logic gate for timing and recording chemical events
Source: Mol Syst Biol. 2016 May 18;12(5):869. doi: 10.15252/msb.20156663 (PMC5289221; doi:10.15252/msb.20156663)
Supplement: Supplementary file 3 — Code EV1 [file MSB-12-869-s003.zip › readme.pdf]

How to use the “Hsiao2016_main_runfile.m” MATLAB script

Contents

- Gillespie SSA Simulations for Hsiao et al, 2016
- Simulation Type 1: Fig.2D, Fig3
- Simulation Type 2: Fig.5
- Simulation Type 2: Fig.S12
- Simulation Type 3: Creates Figure 7 %%%%

Gillespie SSA Simulations for Hsiao et al, 2016

Stochastic simulation main run file

```
% Gillespie stochastic simulation algorithm (Gillespie, 1977)
% Hsiao, VH., Hori, Y., Rothemund, WK., Murray, RM. 2016
% "A population-based temporal logic gate for timing and recording chemical events"
% Updated: March 2016

% Create two sub-folders named 'data' and 'processed_data'
% Raw simulation results will automatically be saved in \data\
% Processed results will be saved in \processed_data\
% Only files in \processed_data\ need to be kept
% This script calls the function Hsiao2016_ssa_main(date, type, cellnum)
% Actual simulation parameters are all set in Hsiao2016_ssa_main.m

% Document created by publishing
% publish('Hsiao2016_main_runfile.m','latex')
```

Simulation Type 1: Fig.2D, Fig3

Creates Fig.2D, Fig.3, Fig.S1, and FigS2

Code:

```
clear all; clc; close all;

% Prefix for data files
mydate = 'Hsiao2016_Sim1';

% Set total number of cells for *each* population
% Manuscript cellnum = 5000
cellnum = 1000;

tic
Hsiao2016_ssa_main(mydate, 1, cellnum);
toc

%%%% Options for fignum: 'fig3', 'Sfig3', 'fig2', 'Sfig2D', 'Sfig2_single'
data_plot_main(1, 'fig2', cellnum, mydate)
data_plot_main(1, 'fig3', cellnum, mydate)
data_plot_main(1, 'Sfig3', cellnum, mydate)
data_plot_main(1, 'Sfig2_single', cellnum, mydate)
data_plot_main(1, 'Sfig2D', cellnum, mydate)
```

Command Line Output:

```
Running simulation type 1
V2:0.4  0.4  0  0  50  50  0.3  10  10
Simulations done
Processing Type 1 Data
Data Processing Done
Saving single data
Done saving single data
Data Processing Done
Elapsed time is 5678.277021 seconds.
```

Resulting Figures:

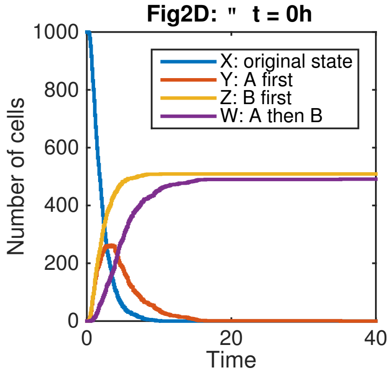

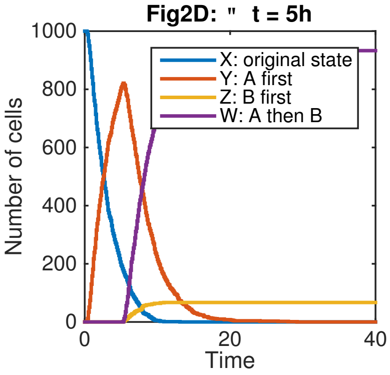

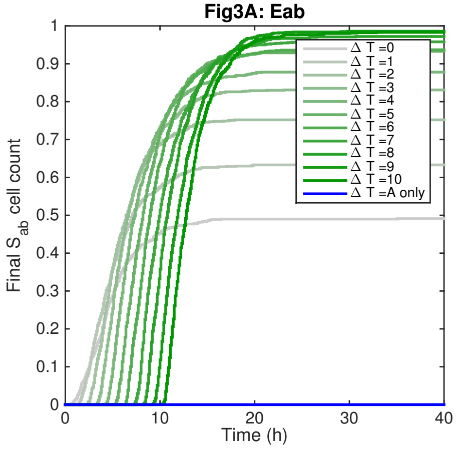

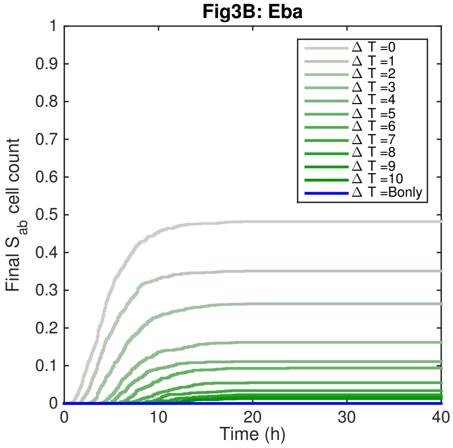

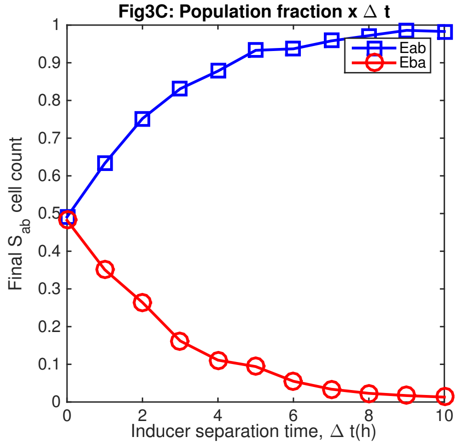

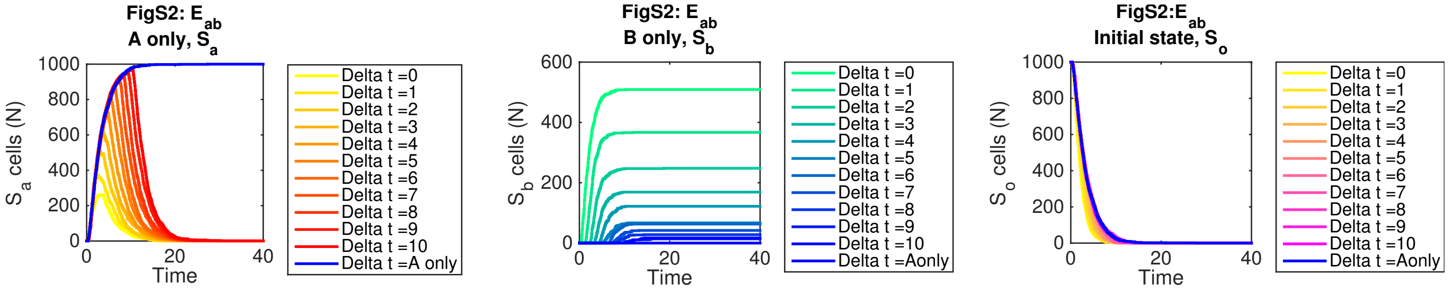
FigS2: E_{ab}
A only, S_a
S_a cells (N)
Time
Delta t =0
Delta t =1
Delta t =2
Delta t =3
Delta t =4
Delta t =5
Delta t =6
Delta t =7
Delta t =8
Delta t =9
Delta t =10
Delta t =A only
FigS2: E_{ab}
B only, S_b
S_b cells (N)
Time
Delta t =Aonly
FigS2:E_{ab}
Initial state, S_o
S_o cells (N)
Time
Delta t =Aonly

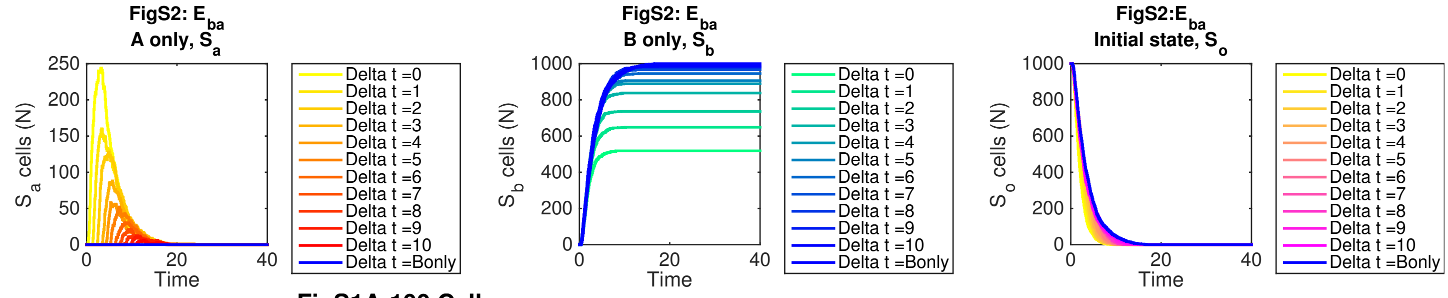
FigS2: E_{ba}
A only, S_a
S_a cells (N)
Time
Delta t =Bonly
FigS2: E_{ba}
B only, S_b
S_b cells (N)
Time
Delta t =Bonly
FigS2:E_{ba}
Initial state, S_o
S_o cells (N)
Time
Delta t =Bonly

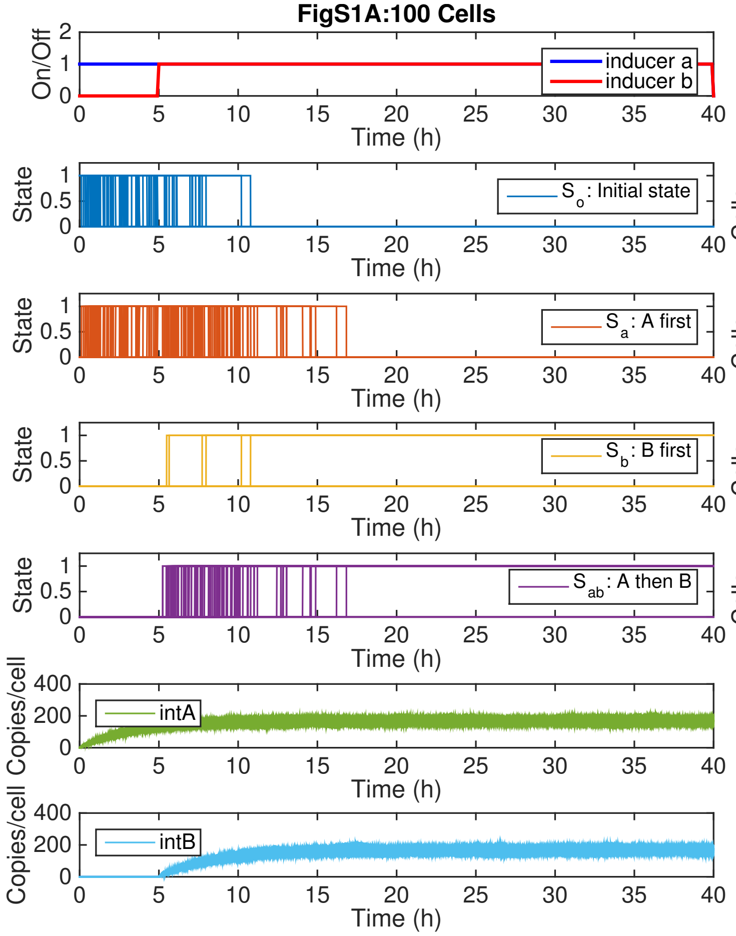
FigS1A:100 Cells
On/Off
inducer a
inducer b
Time (h)
State
S_o: Initial state
S_a: A first
S_b: B first
S_{ab}: A then B
Copies/cell
intA
intB

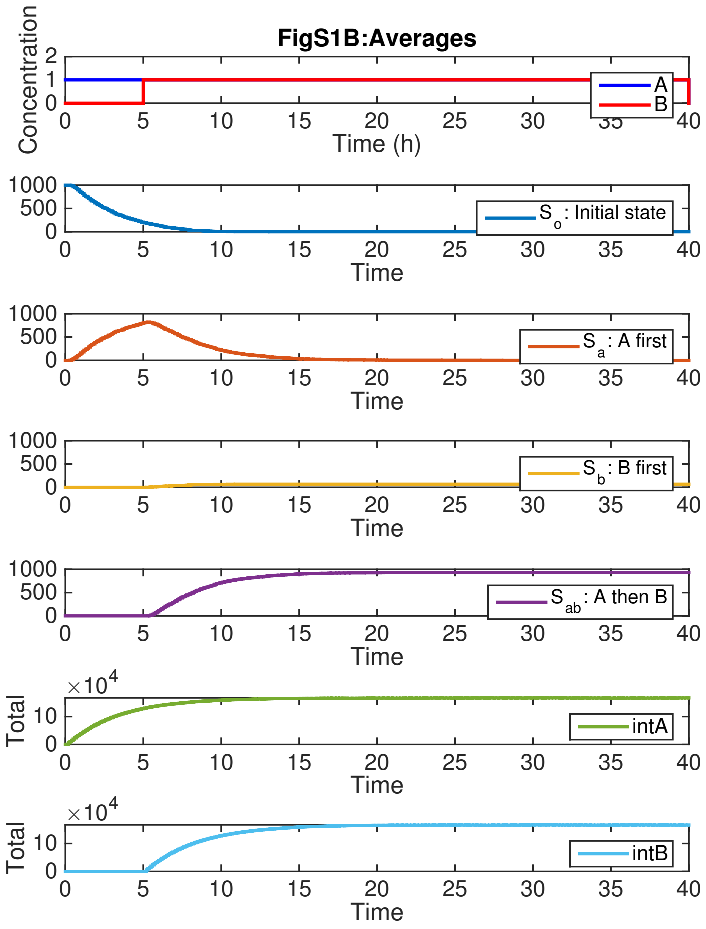
FigS1B:Averages
Concentration
A
B
Time (h)
Cells
S_o: Initial state
S_a: A first
S_b: B first
S_{ab}: A then B
Time
Total
$\times 10^4$
intA
intB

Simulation Type 2: Fig.5

Creates Fig.5, Varying Kflip, Kleak

Code:

```
mydate = 'Hsiao2016_Sim2_Fig5';

% Set total number of cells for *each* population
% Manuscript cellnum = 3000
cellnum = 1000;

tic
Hsiao2016_ssa_main(mydate, 2, cellnum);
toc
data_plot_main(2, 'fig5', cellnum, mydate)
```

Command Line Output:

```
Running simulation type 2
Kflip
Simulations for flipping done1
Data Processing Done
Data Processing Done
Simulations for flipping done2
Data Processing Done
Data Processing Done
Simulations for flipping done3
Data Processing Done
Data Processing Done
Kleak
Simulations for leak done1
Data Processing Done
Data Processing Done
Simulations for leak done2
Data Processing Done
Data Processing Done
Simulations for leak done3
Data Processing Done
Data Processing Done
Kfinal
Simulations 3 done
Data Processing Done
Data Processing Done
Elapsed time is 37455.406030 seconds.
```

Resulting Figures:

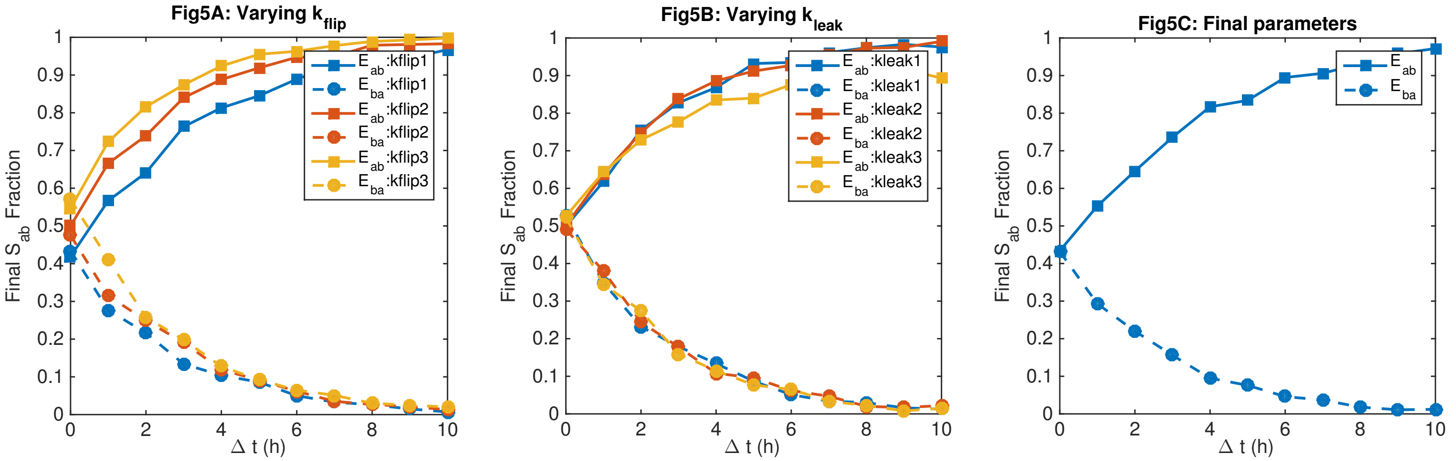

Simulation Type 2: Fig.S12

Creates Fig.S12, Varying Kprod, Kdb, Kdeg

Code:

```
mydate = 'Hsiao2016_Sim2_FigS5';
cellnum = 1000; % Manuscript cellnum = 3000

tic
Hsiao2016_ssa_main(mydate, 4, cellnum);
toc
data_plot_main(4, 'Sfig5', cellnum, mydate)
```

Command Line Output:

```
Running simulation type 4
Kprod:5    10    50   100
Simulations for kprod done1
Data Processing Done
Data Processing Done
Simulations for kprod done2
Data Processing Done
Data Processing Done
Simulations for kprod done3
Data Processing Done
Data Processing Done
Simulations for kprod done4
Data Processing Done
Data Processing Done
Kdb:1    10   100
Simulations for Kdb done1
Data Processing Done
Data Processing Done
Simulations for Kdb done2
Data Processing Done
Data Processing Done
Simulations for Kdb done3
Data Processing Done
Data Processing Done
Kdeg:0         0.3          3
```

```
Simulations for Kdeg done1
Data Processing Done
Data Processing Done
Simulations for Kdeg done2
Data Processing Done
Data Processing Done
Simulations for Kdeg done3
Data Processing Done
Data Processing Done
Elapsed time is 47141.883561 seconds.
```

Resulting Figures:

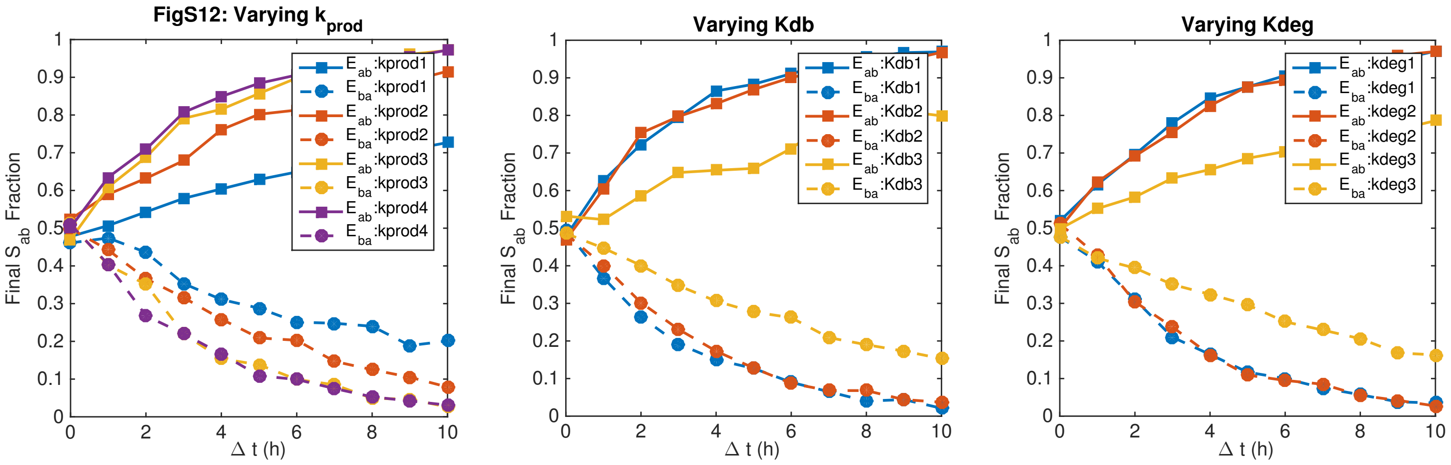

Simulation Type 3: Creates Figure 7 %%%%

Creates Fig.6DEF, Fig.7A, Fig.S14,S15

Code:

```
mydate = 'Hsiao2016_Sim3_Fig7'; % Date to name files

% Set total number of cells for *each* population
% Manuscript cellnum = 3000
cellnum = 1000;

tic
Hsiao2016_ssa_main(mydate, 3, cellnum);
toc
data_plot_main(3, 'fig7', cellnum, mydate)
```

Command Line Output:

```
Running simulations type 3
Elapsed time is 1146.230573 seconds.
Elapsed time is 1145.837409 seconds.
Elapsed time is 1147.090676 seconds.
Elapsed time is 1148.023863 seconds.
Elapsed time is 1145.993834 seconds.
Elapsed time is 1146.532994 seconds.
Elapsed time is 1146.264670 seconds.
Elapsed time is 1146.449894 seconds.
```

```
Elapsed time is 1146.021011 seconds.
Elapsed time is 1144.892608 seconds.
Elapsed time is 1144.795203 seconds.
Elapsed time is 1143.945297 seconds.
Elapsed time is 1145.747752 seconds.
Simulations done
Processing data
Data Processing Done
item number:1
Elapsed time is 711.984393 seconds.
Data Processing Done
item number:2
Elapsed time is 713.511557 seconds.
Data Processing Done
item number:3
Elapsed time is 700.598618 seconds.
Data Processing Done
item number:4
Elapsed time is 700.555967 seconds.
Data Processing Done
item number:5
Elapsed time is 698.845426 seconds.
Data Processing Done
item number:6
Elapsed time is 706.033660 seconds.
Data Processing Done
item number:7
Elapsed time is 700.265251 seconds.
Data Processing Done
item number:8
Elapsed time is 700.030997 seconds.
Data Processing Done
item number:9
Elapsed time is 718.108941 seconds.
Data Processing Done
item number:10
Elapsed time is 701.260479 seconds.
Data Processing Done
item number:11
Elapsed time is 700.598988 seconds.
Data Processing Done
item number:12
Elapsed time is 704.868422 seconds.
Data Processing Done
item number:13
Elapsed time is 701.969828 seconds.
Data saved
Elapsed time is 702.649222 seconds.
```

Resulting Figures:

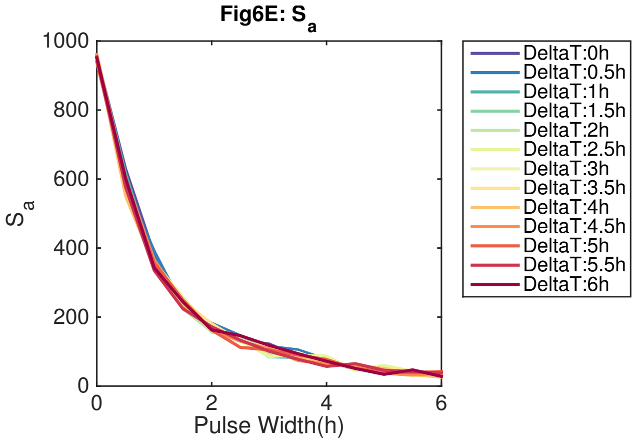

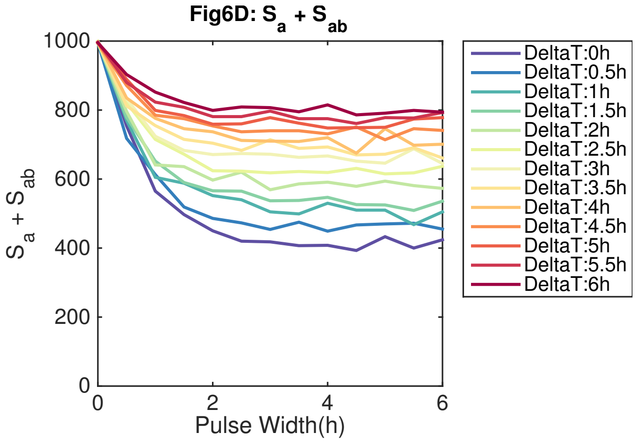

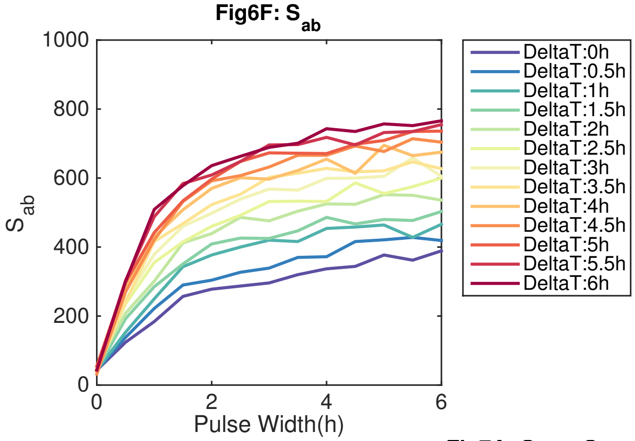

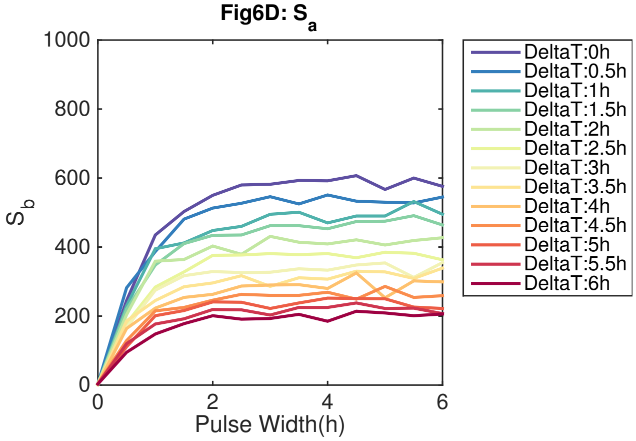

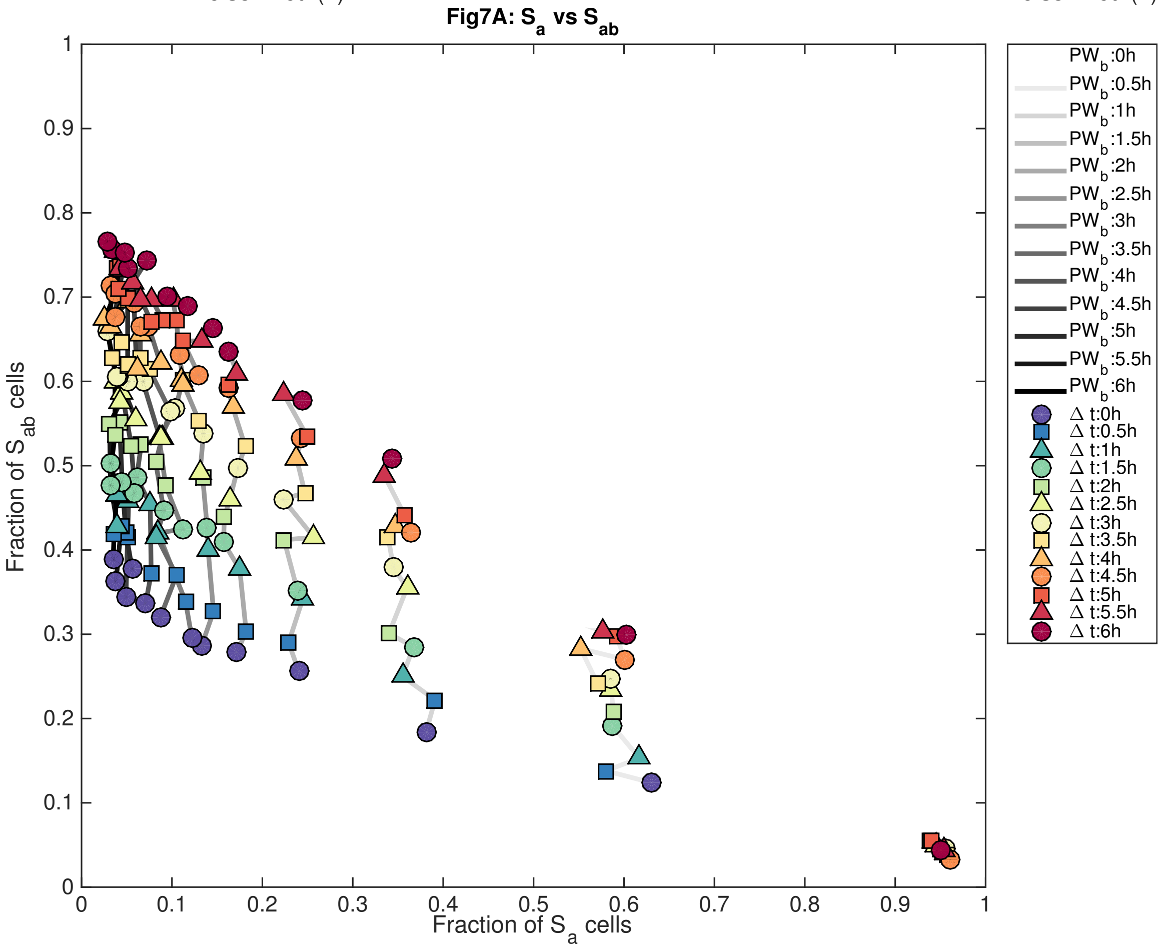

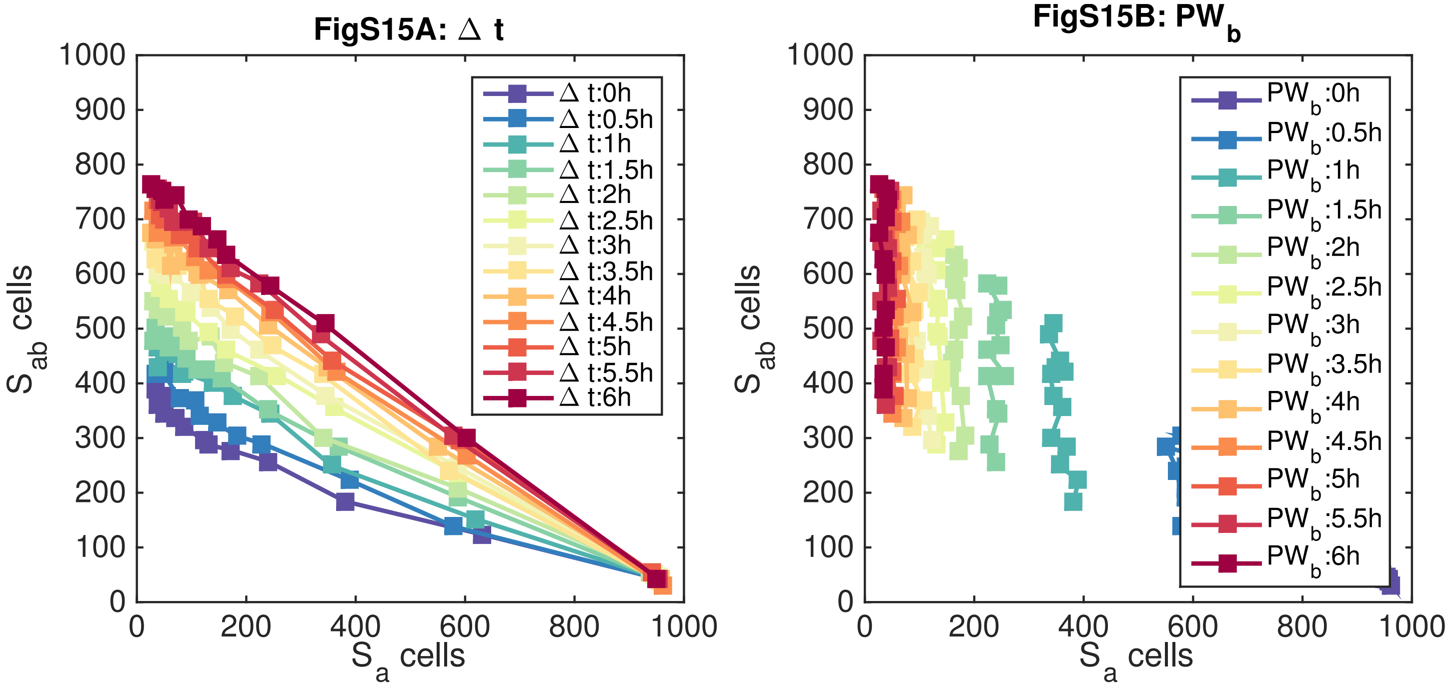
FigS15A: Δ t
Δ t:0h
Δ t:0.5h
Δ t:1h
Δ t:1.5h
Δ t:2h
Δ t:2.5h
Δ t:3h
Δ t:3.5h
Δ t:4h
Δ t:4.5h
Δ t:5h
Δ t:5.5h
Δ t:6h
S_{ab} cells
S_a cells
FigS15B: PW_b
PW_b:0h
PW_b:0.5h
PW_b:1h
PW_b:1.5h
PW_b:2h
PW_b:2.5h
PW_b:3h
PW_b:3.5h
PW_b:4h
PW_b:4.5h
PW_b:5h
PW_b:5.5h
PW_b:6h
S_{ab} cells
S_a cells
