# Supplementary material for: A population‐based temporal logic gate for timing and recording chemical events
Source: Mol Syst Biol. 2016 May 18;12(5):869. doi: 10.15252/msb.20156663 (PMC5289221; doi:10.15252/msb.20156663)
Supplement: Supplementary file 7 — Source Data for Figure 8 [file MSB-12-869-s006.zip › Hsiao2016_Fig8.pdf]

Contents

- MATLAB script for curve fitting, Fig.8
- Load data from Excel file
- Generate fits to find fitted parameters (Appendix Fig.S24)
- Final fitted parameters used in Fig. 8
- Plot of Estimated vs actual PW and DT (Fig. 8)
- Resolution for determining PWb and Delta t from pop. distribution (Fig.S25)
- Generate table (Appendix Table S4)

MATLAB script for curve fitting, Fig.8

Calibration of experimental data by fitting to RFP vs GFP pop. fraction data

```
% Hsiao, VH., Hori, Y., Rothemund, WK., Murray, RM. 2016
% "A population-based temporal logic gate for timing and recording chemical events"
% Updated: March 2016

% Document created by publishing
% publish('Hsiao2016_Fig8.m','html')
```

Load data from Excel file

```
clear all; clc; close all;
name = 'Hsiao2016_Fig8';
date = 'Hsiao2016_Fig8';

GFP = xlsread(strcat(name,'.xlsx'),'GFP24');
RFP = xlsread(strcat(name,'.xlsx'),'RFP24');

dtname = {'Ctrl';'0h';'1h';'2h';'3h';'4h';'5h';'6h'};
pwname = {'0h';'1h';'2h';'3h';'4h';'5h';'6h'};

pw = 0:1:6;
dt = 0:1:6;
```

Generate fits to find fitted parameters (Appendix Fig.S24)

Fit a function for PW_b as a function of RFP population % Fit a function for DT as a function of both GFP pop. % and PW_b

```
figure('Position', [100, 100, 1000, 750]);

cmap1 = lines(8); % Color scheme
range = 1:7; % Row 8 (excluded in the fit) is a 30 second pulse

% Subplot 1 is RFP vs PW_b experimental data
subplot(2,3,1); hold all;

% Control with no inducers
h0 = plot(RFP(range,1),pw,'-');
set(h0,'color','k','linewidth',2)

% Plot varying delta T curves
for i = 2:8
    h0 = plot(RFP(range,i),pw,'-o','markerfacecolor',cmap1(i-1,:));
    set(h0,'color',cmap1(i-1,:),'linewidth',2)
end
legend(strcat('dt:',dtname),'location','northeast');
box on;
xlabel('RFP fraction(%)');
set(gca,'fontsize',12);
ylabel('PW_b'),axis([0 70 -0.1 6.5])
axis square; box on;
title('Fig.S24A: Original Data')

% Subplot 2 is the same data but with a curve fit
subplot(2,3,2); hold all;
colormap(cmap1)
x = reshape(RFP(1:7,2:8),[49 1]);
y = reshape([pw' pw' pw' pw' pw' pw' pw'],[49 1]);

% original data
for i = 2:8
```

```
    h0 = plot(RFP(range,i),pw,'o');
    set(h0,'color',cmap1(i-1,:),'markersize',5,'linewidth',1,'markerfacecolor',cmap1(i-1,:))
end

% plot fit
[f1,gof1] = fit(x,y,'power2');
h = plot(f1,'-'); set(h, 'linewidth',2,'color','r')

ylabel('PW_b'),axis([0 70 -0.1 6.5])
axis square; box on;
xlabel('RFP fraction(%)');
set(gca,'fontsize',12)
title({'Fig.24B: Curve fit to RFP','f1(x) = ax^b+c',strcat(strcat('a = ',num2str(f1.a)),',', strcat('b = ',num2str(f1.b)),',',strcat('c
= ',num2str(f1.c)))})

% Delta t fits as a function of GFP and PW_b
% Subplot 3 is plotting GFP (%) versus Delta t

subplot(2,3,4); hold all;
plot(GFP(1,2:8),dt,'o-','markerface','k','color','k','linewidth',2);

for i = 1:7
    plot(GFP(i,2:8),dt,'o-','markerface',cmap1(i,:),'color',cmap1(i,:),'linewidth',2);
end
legend(strcat('dt:',dtname),'location','northwest');
xlabel('GFP fraction(%)');
ylabel('\Delta t'), axis([0 70 0 6.5])
set(gca,'fontsize',12);
axis square; box on;
title('Fig.S24C: Original Data')

% Subplot 4 is fitting curves for Delta t as a function
subplot(2,3,5); hold all;
clear a2 c2

% Automated f2b optimization
% This is to the find the best power coefficient for the PWb function
% If you include it in the curve fitting then the results are weird

f2b_range = 0:0.5:3;
f2b_best = 0;
rsq_old = 0;
for i = 1:length(f2b_range)
    myFun = strcat('a*x^',num2str(f2b_range(i)),' + c');
    for j = 1:7
        [f2,gof2] = fit(GFP(j,2:8)',dt',myFun,'StartPoint',[1 1]);
        rsq(j) = gof2.rsquare;
    end
    rsq_new = mean(rsq(2:end));
    if rsq_new > rsq_old
        f2b_best = f2b_range(i);
        rsq_old = rsq_new;
    end
end
disp(strcat('best f2b: ',num2str(f2b_best)));
disp(strcat('best rsq: ',num2str(rsq_old)));

f2b = f2b_best;

myFun = strcat('a*x^',num2str(f2b),' + c');
i = 1;
for j = 1:7

    [f2,gof2] = fit(GFP(j,2:8)',dt',myFun,'StartPoint',[0 0]);
    rsq(i) = gof2.rsquare;
    a2(i) = f2.a;
    c2(i) = f2.c;

    h = plot(f2,GFP(j,2:8),dt,'o');
    set(h,'markerfacecolor',cmap1(i,:),'color',cmap1(i,:),'linewidth',2)
    i = i+1;
end

xlabel('GFP fraction(%)');
ylabel('\Delta t'); set(gca,'fontsize',12)
axis square; box on;
legend off
title({'Fig24D:Fitting GFP to \Delta t',strcat('f2(x) = a*x^{',num2str(f2b),'}+ c')});

[f2a,gof2a] = fit(pw(1:7)',a2','exp2');
[f2c,gof2c] = fit(pw(1:7)',c2','exp2');
```

```
axis([0 70 -1 7])

% Subplot 5 is fitting curves for a2 and c2 coefficients as functions of PW_b

subplot(2,3,6); hold all;
h4 = plot(f2a,pw(1:7),a2,'or'); set(h4,'markerfacecolor','r','color','r');
h6 = plot(f2c,pw(1:7),c2,'ob'); set(h6,'markerfacecolor','b','color','b');

legend('a2','exp2 fit','c2','exp2 fit','location','southwest')
axis square, box on;
title('f2c(x) = a*exp(b*x) + c*exp(d*x)')
xlabel('PW_b'); ylabel('fitted coefficient value');
set(gca,'fontsize',12)
title('Fig.S24E: Fitting \Delta t coefficients as f(PW_b)')
```

```
Warning: Power functions require x to be positive. Non-positive data are treated
as NaN.
best f2b:1.5
best rsq:0.97649
```

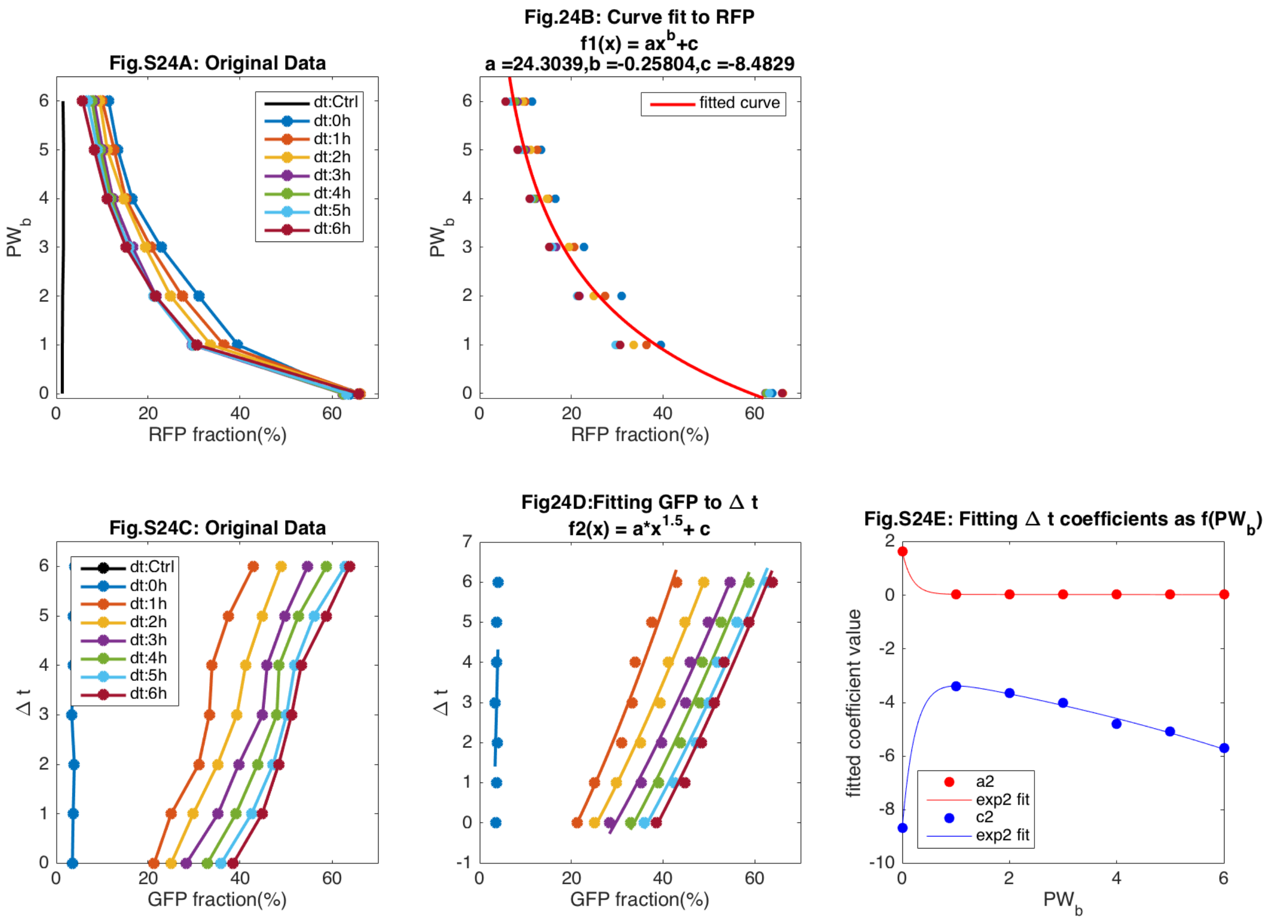

Final fitted parameters used in Fig. 8

```
%%%% Fits for PW as a function of RFP only %%%%
% f1 =
%      General model Power2:
%      f1(x) = a*x^b+c
%      Coefficients (with 95% confidence bounds):
%        a =       24.3  (18.75, 29.86)
%        b =     -0.258  (-0.4956, -0.02047)
%        c =      -8.483  (-18.75, 1.784)
% PW(RFP) = a*RFP^b + c

%%%% Find DT as a function of GFP %%%%
% f2 =
%      General model:
```

```
%      f2(x) = a*x^1 + c

% Find coefficients a and c as functions of PW
%
% f2a(x) = a*exp(b*x) + c*exp(d*x)
%      Coefficients (with 95% confidence bounds):
%        a =       1.608  (1.602, 1.615)
%        b =      -5.587  (-6.513, -4.661)
%        c =     0.02982  (0.02412, 0.03552)
%        d =    -0.04646  (-0.09342, 0.0004906)
%
%      General model Exp2:
% f2c(x) = a*exp(b*x) + c*exp(d*x)
%      Coefficients (with 95% confidence bounds):
%        a =      -5.742  (-6.332, -5.153)
%        b =      -4.115  (-10.11, 1.877)
%        c =      -2.944  (-3.353, -2.534)
%        d =      0.1113  (0.08146, 0.1412)
```

Plot of Estimated vs actual PW and DT (Fig. 8)

Using the fitted parameters, we can generate predicted PWb and Delta t values based on real data

```
% Subplot 1: Plot mesh of fitted lines with experimental data
figure('Position', [100, 100, 1000, 750]);

subplot(1,3,1);  hold all;

dt = 0:1:6;
p = 0:1:6;
R = zeros(length(dt),length(p));
G = zeros(length(dt),length(p));

PW_a = f1.a;
PW_b = f1.b;
PW_c = f1.c;

DT_aa = f2a.a;
DT_ab = f2a.b;
DT_ac = f2a.c;
DT_ad = f2a.d;

DT_b = f2b;

DT_ca = f2c.a;
DT_cb = f2c.b;
DT_cc = f2c.c;
DT_cd = f2c.d;


for i = 1:length(p)
    for j = 1:length(dt)
    R(i,j) = ((p(i)- PW_c)/PW_a)^(1/PW_b);
    DT_a = DT_aa*exp(DT_ab*p(i)) + DT_ac*exp(DT_ad*p(i));
    DT_c = DT_ca*exp(DT_cb*p(i)) + DT_cc*exp(DT_cd*p(i));

    G(i,j) = ((dt(j)- DT_c)/DT_a)^(1/DT_b);
    end
end

cmap = gray(length(R)+1);
for i = 1:length(cmap)
    cmap1(i,:) = cmap(end-i+1);
end
cmap2 = jet(length(R));

for i =1:length(R)
    plot(R(:,i),G(:,i),'-','linewidth',1.5,'color',cmap2(i,:));
end
for i =1:length(R)
    plot(R(i,:),G(i,:),'-','linewidth',1.5,'color',cmap1(i+1,:));
end

% Plot experimental data

% Zero PW control
plot(RFP(1,2:8),GFP(1,2:8),'o','color',cmap1(2,:),'markerfacecolor',cmap1(2,:),'linewidth',1,'markersize',6,'markeredgecolor','k')

% Plot with lines showing same PW
```

```
i = 2;
for j = 2:7;
    plot(RFP(j,2:8),GFP(j,2:8),'--','color',cmap1(i+1,:),'markerfacecolor',cmap1(i+1,:),'linewidth',1)
    i = i + 1 ;
end

% Plot with colored markers showing same DT
i = 1;
for j = 2:8;
    plot(RFP(2:7,j),GFP(2:7,j),'o','color',cmap2(i,:),'markerfacecolor',cmap2(i,:),'linewidth',1, 'markersize',6,'markeredgecolor','k')
    i = i +1 ;
end

axis square, box on;

xlabel('RFP fraction(%)'); ylabel('GFP fraction(%)');
set(gca,'fontsize',12)
axis([0 70 0 70])
legend('pw0','pw1','pw2','pw3','pw4','pw5','pw6')
title('Fig.8A: Fitted mesh over data')

% Subplot 2 is Actual versus Estimated PWb

% calculate PW_b based on experimental RFP fit
r = RFP(1:7,2:8);
y = [pw' pw' pw' pw' pw' pw' pw'];

pw_est = zeros(size(r));
pw_err = zeros(size(r));

for i = 1: size(r,1)
    for j = 1: size(r,2)
        pw_est(i,j) = PW_a*r(i,j)^(PW_b) + PW_c ;
        pw_err(i,j) = pw_est(i,j) - y(i,j);
    end
end

pw_sd = zeros(7,1); pw_avg = zeros(7,1);

for i = 1:7
    pw_sd(i,:) = std(pw_est(i,:));
    pw_avg(i,:) = mean(pw_est(i,:));
end

subplot(1,3,2); hold all;

% Plot standard deviations as a scale bar for prediction resolution
errorbar(-0.3:1:5.9, pw_avg,pw_sd,'ko','markerfacecolor','k');

for i = 1:size(r,2);
    plot(y(i,:), pw_est(i,:),'o','markerfacecolor',cmap1(i,:),'color','k')
end
plot(0:0.1:6, 0:0.1:6,'color','r')

axis square; box on;
xlabel('Actual PW_b'); ylabel('Estimated PW_b')
axis([-1 7 -1 7])
set(gca,'fontsize',12)
set(gca,'ygrid','on','xgrid','on')
title('Fig.8B: Actual vs Est. PWb')


% Subplot 3: Plot actual Delta t versus Estimated Delta t

% Exclude PW_b = 0 case since there is no second inducer
% If there is no second inducer, Delta t does not exist

g = GFP(2:7,2:8);
z = [dt; dt; dt; dt; dt; dt];

pw_est2 = pw_est(2:7,:) ;

dt_est = zeros(size(g));
for i = 1:size(g,1)
    for j = 1:size(g,2)
        DT_a = DT_aa*exp(DT_ab*pw_est2(i,j)) + DT_ac*exp(DT_ad*pw_est2(i,j));
        DT_c = DT_ca*exp(DT_cb*pw_est2(i,j)) + DT_cc*exp(DT_cd*pw_est2(i,j));
        dt_est(i,j) = DT_a*g(i,j)^DT_b + DT_c;
    end
end
```

```
subplot(1,3,3); hold all;

for i = 1:7
    dt_sd(i) = std(dt_est(:,i));
    dt_avg(i) = mean(dt_est(:,i));
end

% Plot standard deviations as a scale bar for prediction resolution
errorbar(-0.3:1:5.9, dt_avg, dt_sd,'ko','markerfacecolor','k');

for i = 1:7
    plot(z(:,i), dt_est(:,i),'o','markerfacecolor',cmap2(i,:),'color','k')
end
plot(0:0.1:6, 0:0.1:6,'color','r')
axis square; box on;
xlabel('Actual \Delta t'); ylabel('Estimated \Delta t')
axis([-1 7 -1 7])
set(gca,'fontsize',12)
set(gca,'ygrid','on','xgrid','on')
title('Fig.8C: Actual vs Est. \Delta t')
```

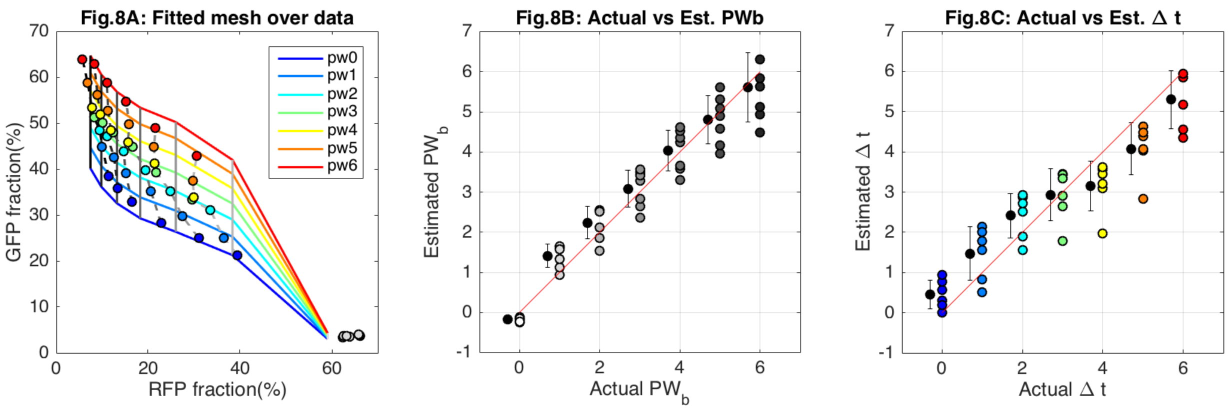

Resolution for determining PWb and Delta t from pop. distribution (Fig.S25)

Make sure you run the Fig 8 script first (above) It generates all the variables needed for subplots 4 and 5

```
figure('Position', [100, 100, 1000, 750]);

cmap2 = jet(8);
cmap = gray(9);
for i = 1:8
    cmap1(i,:) = cmap(9-i,:);
end

subplot(2,3,1); hold all;
j = 1; i = 1;
plot(RFP(1,2:8),GFP(1,2:8),'-o','color',cmap1(i,:),'markerfacecolor',cmap1(i,:),'linewidth',2)
```

```
i = 2;
for j = 2:7;
    plot(RFP(j,2:8),GFP(j,2:8),'-o','color',cmap1(i,:),'markerfacecolor',cmap1(i,:),'linewidth',2)
    i = i + 1 ;
end
i = 1;
for j = 2:8;
    plot(RFP(2:7,j),GFP(2:7,j),'-o','color',cmap2(i,:),'markerfacecolor',cmap2(i,:),'linewidth',2)
    i = i +1 ;
end
axis square; box on;
xlabel('RFP fraction(%)'); ylabel('GFP fraction(%)');
set(gca,'fontsize',12)
axis([0 70 0 70])
title({'Fig.S25A: Experimental data:','Squares indicate resolution of PW_b/\Delta t'})

dt = 0:1:6;
p = 0:1:6;
R = zeros(length(dt),length(p));
G = zeros(length(dt),length(p));

for i = 1:length(p)
    for j = 1:length(dt)
    R(i,j) = ((p(i)- PW_c)/PW_a)^(1/PW_b);
    DT_a = DT_aa*exp(DT_ab*p(i)) + DT_ac*exp(DT_ad*p(i));
    DT_c = DT_ca*exp(DT_cb*p(i)) + DT_cc*exp(DT_cd*p(i));
    G(i,j) = ((dt(j)- DT_c)/DT_a)^(1/DT_b);
    end
end

cmap = gray(length(R)+1);
for i = 1:length(cmap)
    cmap1(i,:) = cmap(end-i+1);
end
cmap2 = jet(length(R));

subplot(2,3,2);  hold all;

for i =1:length(R)
    plot(R(:,i),G(:,i),'-','linewidth',2,'color',cmap2(i,:));
end
for i =1:length(R)
    plot(R(i,:),G(i,:),'-','linewidth',2,'color',cmap1(i+1,:));
end

% Plot experimental data

% Plot with lines showing same PW
i = 2;
for j = 2:7;
    plot(RFP(j,2:8),GFP(j,2:8),'--','color',cmap1(i+1,:),'markerfacecolor',cmap1(i+1,:),'linewidth',1)
    i = i + 1 ;
end

axis square, box on;

xlabel('RFP fraction(%)'); ylabel('GFP fraction(%)');
set(gca,'fontsize',12)
axis([0 70 0 70])
title({'Fig25B:', 'Fitted PW_b curves vs data'})

subplot(2,3,3);  hold all;

for i =1:length(R)
    plot(R(:,i),G(:,i),'-','linewidth',2,'color',cmap2(i,:));
end
for i =1:length(R)
    plot(R(i,:),G(i,:),'-','linewidth',2,'color',cmap1(i+1,:));
end

% Plot experimental data

% Zero PW control
plot(RFP(1,2:8),GFP(1,2:8),'o','color',cmap1(2,:),'markerfacecolor',cmap1(2,:),'linewidth',1,'markersize',6)

% Plot with colored markers showing same DT
i = 1;
for j = 2:8;
    plot(RFP(2:7,j),GFP(2:7,j),'o','color',cmap2(i,:),'markerfacecolor',cmap2(i,:),'linewidth',1, 'markersize',6)
    i = i +1 ;
end
```

```
axis square, box on;

xlabel('RFP fraction(%)'); ylabel('GFP fraction(%)');
set(gca,'fontsize',12)
axis([0 70 0 70])
title({'Fig25C:', 'Fitted \Delta t curves vs data'})

% Add resolution fits data
% Make sure you run the Fig 8 script first (generates all the variables)

subplot(2,3,4); hold all;
% Plot standard deviations as a scale bar for prediction resolution
errorbar(-0.3:1:5.9, pw_avg,pw_sd,'ko','markerfacecolor','k');

for i = 1:size(r,2);
    plot(y(i,:), pw_est(i,:),'o','markerfacecolor',cmap1(i,:),'color','k')
end
plot(0:0.1:6, 0:0.1:6,'color','r')

axis square; box on;
xlabel('Actual PW_b'); ylabel('Estimated PW_b')
axis([-1 7 -1 7])
set(gca,'fontsize',12)
set(gca,'ygrid','on','xgrid','on')
title('Fig25D: Est. vs Actual PWb')

subplot(2,3,5); hold all;
% Plot standard deviations as a scale bar for prediction resolution
errorbar(-0.3:1:5.9, dt_avg, dt_sd,'ko','markerfacecolor','k');

for i = 1:7
    plot(z(:,i), dt_est(:,i),'o','markerfacecolor',cmap2(i,:),'color','k')
end
plot(0:0.1:6, 0:0.1:6,'color','r')
axis square; box on;
xlabel('Actual \Delta t'); ylabel('Estimated \Delta t')
axis([-1 7 -1 7])
set(gca,'fontsize',12)
set(gca,'ygrid','on','xgrid','on')
title('Fig.S25E, Est vs Actual \Delta t')
```

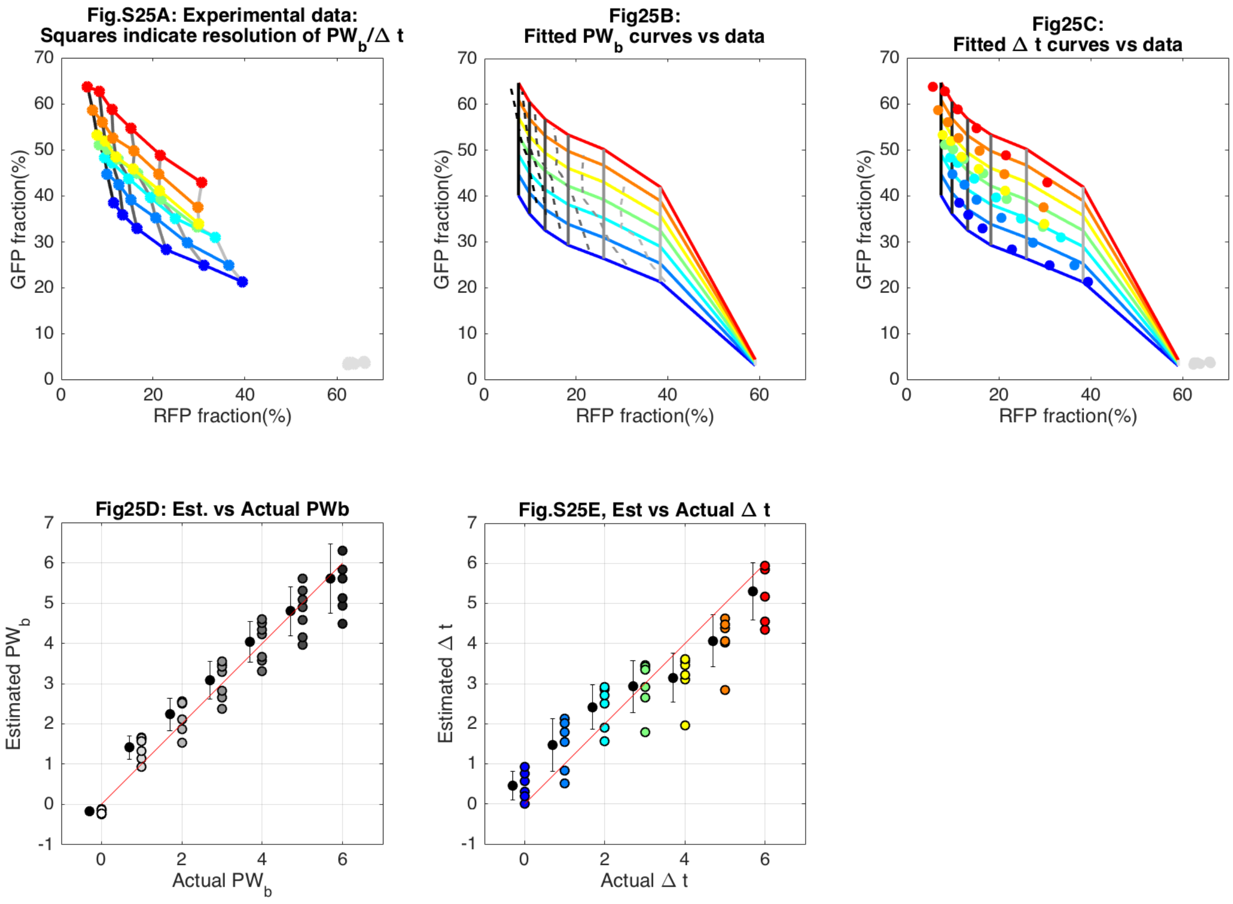

Generate table (Appendix Table S4)

```
clear Rfit Gfit PWfit DTfit

Rfit = [1,10,20,30,40,50,60];
Gfit = [1,10,20,30,40,50,60];

PWfit = zeros(length(Rfit),length(Rfit));
DTfit = zeros(length(Rfit),length(Rfit));

PW_a = 24.3;
PW_b = -0.258;
PW_c = -8.483;

DT_aa = 1.608;
DT_ab = -5.587;
DT_ac = 0.029;
DT_ad = -0.046;

DT_b = 1.5;

DT_ca = -5.742;
DT_cb = -4.115;
DT_cc = -2.944;
DT_cd = 0.113;

for i = 1:length(Rfit)
    for j = 1:length(Gfit)
    PWfit(i, j) = PW_a*Rfit(i)^PW_b + PW_c;
    DT_a = DT_aa*exp(DT_ab*PWfit(i,j)) + DT_ac*exp(DT_ad*PWfit(i,j));
    DT_c = DT_ca*exp(DT_cb*PWfit(i,j)) + DT_cc*exp(DT_cd*PWfit(i,j));
    DTfit(i, j) = DT_a*Gfit(j)^DT_b + DT_c;
    end
end

DTfit = round(DTfit,1);
PWfit = round(PWfit,1);
```

```
% Replace negative values with zero
DTfit(DTfit<0) = 0;
PWfit(PWfit<0) = 0;

% Show tables in command window
DTfit, PWfit
```

```
DTfit =

         0         0         0         0         0         0         0
         0         0         0         0    0.7000    3.0000    5.6000
         0         0         0    0.2000    2.5000    5.0000    7.9000
         0         0         0    0.9000    3.3000    6.0000    9.1000
         0         0         0    2.9000    6.3000   10.2000   14.5000
         0    2.9000   16.1000   33.1000   53.3000   76.2000  101.6000
         0   52.6000  166.3000  313.4000  487.7000  685.4000  903.9000


PWfit =

   15.8000   15.8000   15.8000   15.8000   15.8000   15.8000   15.8000
    4.9000    4.9000    4.9000    4.9000    4.9000    4.9000    4.9000
    2.7000    2.7000    2.7000    2.7000    2.7000    2.7000    2.7000
    1.6000    1.6000    1.6000    1.6000    1.6000    1.6000    1.6000
    0.9000    0.9000    0.9000    0.9000    0.9000    0.9000    0.9000
    0.4000    0.4000    0.4000    0.4000    0.4000    0.4000    0.4000
         0         0         0         0         0         0         0
```

Published with MATLAB® R2015b
